# Concerted expansion and contraction of immune receptor gene repertoires in plant genomes

**DOI:** 10.1038/s41477-022-01260-5

**Published:** 2022-10-14

**Authors:** Bruno Pok Man Ngou, Robert Heal, Michele Wyler, Marc W. Schmid, Jonathan D. G. Jones

**Affiliations:** 1 The Sainsbury Laboratory, University of East Anglia, Norwich Research Park, Norwich, UK; 2 RIKEN Center for Sustainable Resource Science, Yokohama, Japan; 3 MWSchmid GmbH, Glarus, Switzerland

**Keywords:** Pattern recognition receptors in plants, Effectors in plant pathology, Plant evolution, Natural variation in plants

## Abstract

Recent reports suggest that cell-surface and intracellular immune receptors function synergistically to activate robust defence against pathogens, but whether they co-evolve is unclear. Here we determined the numbers of cell-surface and intracellular immune receptors in 350 species. Surprisingly, the number of receptor genes that are predicted to encode cell-surface and intracellular immune receptors is strongly correlated. We suggest this is consistent with mutual potentiation of immunity initiated by cell-surface and intracellular receptors being reflected in the concerted co-evolution of the size of their repertoires across plant species.

## Abstract (web summary)

Comparative genomic analysis of 350 plant species reveals that cell-surface and intracellular immune receptor gene families co-expand or co-contract. This suggests an evolutionary relationship between the two branches of the plant immune system.

## Main

Plants have evolved a two-tier immune system that recognizes and activates defence against pathogens^1,2^. Cell-surface pattern-recognition receptors (PRRs) recognize apoplastic and usually conserved pathogen-associated molecular patterns (PAMPs) and activate pattern-triggered immunity (PTI). Virulent pathogens secrete effector molecules into plant cells that suppress PTI and promote infection. Intracellular nucleotide-binding leucine-rich repeat (NLR) receptors recognize effectors and activate effector-triggered immunity (ETI). Although PTI and ETI were envisaged as two independent immune systems^1^, emerging evidence suggests they are inter-dependent and share multiple signalling components^3–6^. Thus, PTI and ETI function synergistically to provide robust immunity against pathogens. As PRRs and NLRs are functionally inter-dependent, in this Brief Communication, we investigated whether the sizes of these two receptor gene families are correlated.

**Extended Data Fig. 1:**
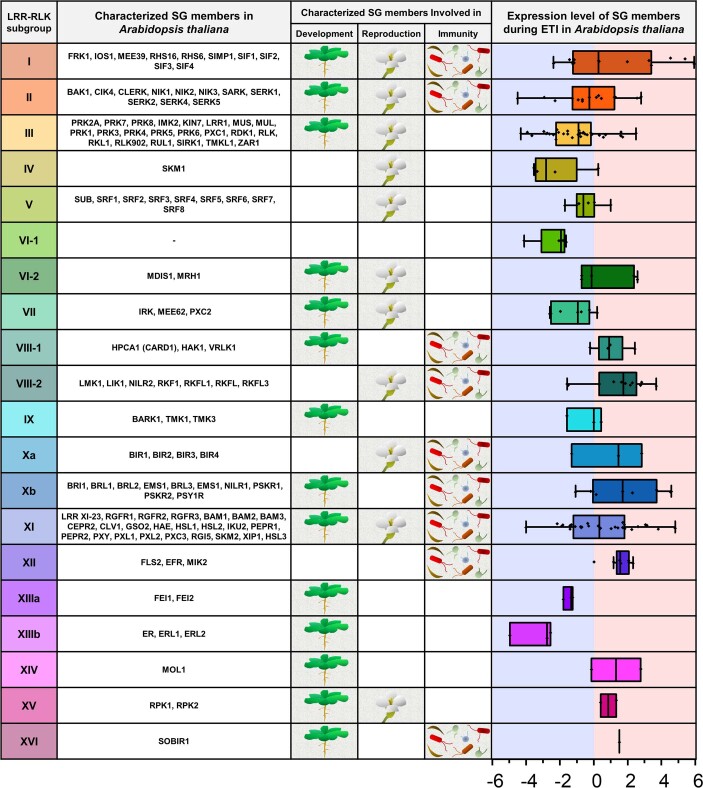
LRR-RLK subgroups in plants. Table representing the characterized subgroup members in *Arabidopsis thaliana*; the biological processes of which the characterized members are involved in, and expression of subgroup members during effector-triggered immunity (ETI). Red shade represents increased expression and blue shade represents decreased expression during ETI. X-axis values represents log_2_(fold change during ETI relative to untreated samples). Box-plot elements: centre line, median; bounds of box, 25th and 75th percentiles; whiskers, 1.5 × IQR from 25th and 75th percentiles. RNA-seq data analysed here were reported previously^3^. ETI is activated by estradiol-induced expression of AvrRps4 in Arabidopsis thaliana for 4 hours. Number of genes (n) from each LRR-RLK subgroup: I, n = 15; II, n = 15; III, n = 33; IV, n = 4; V, n = 6; VI-1, n = 4; V1-2, n = 5; VII, n = 7; VIII-1, n = 4; VIII-2, n = 12; IX, n = 3; Xa, n = 3; Xb, n = 9; XI, n = 28; XII, n = 8; XIIIa, n = 3; XIIIb, n = 3; XIV, n = 2; XV, n = 2; XVI, n = 1.

Plant PRR proteins are structurally diverse but are usually receptor-like kinases (RLKs) or receptor-like proteins (RLPs). RLKs carry extracellular ectodomains and cytosolic kinase domains, while RLPs lack cytosolic kinase domains. RLKs carry multiple types of extracellular domains, such as leucine-rich repeats (LRRs), lectins and lysM motifs (LysMs)^7^. LRR-domain-containing RLKs (LRR-RLKs) and RLPs (LRR-RLPs) are the largest RLK- and RLP-gene families in plants^8,9^. LRR-RLKs can be further classified into 20 subgroups, with each subgroup involved in different biological processes^10^ (Extended Data Fig. 1). For example, BAK1 (BRI1-ASSOCIATED RECEPTOR KINASE) family proteins function as PRR co-receptors and belong to LRR-RLK-II (ref. ^11^). Members of LRR-RLK-XI are involved in recognition of self-peptides^12,13^. Members of LRR-RLK-XII, such as FLAGELLIN-SENSITIVE 2 (FLS2), EF-TU RECEPTOR (EFR) and Xa21 (refs. ^2,7^), are involved in detecting pathogen-derived molecules (Extended Data Fig. 1). NLRs are intracellular receptors that carry NB-ARC domains with C-terminal LRR domains and N-terminal domains, usually comprising either coiled-coil (CC), Toll/interleukin-1 receptor/resistance protein (TIR) or RPW8-like coiled-coil (RPW8) domains (hence, CC-NLRs (or CNLs), TIR-NLRs (TNLs) and RPW8-NLRs (RNLs))^14,15^.

## Identification of immune receptors from plant genomes

**Extended Data Fig. 2:**
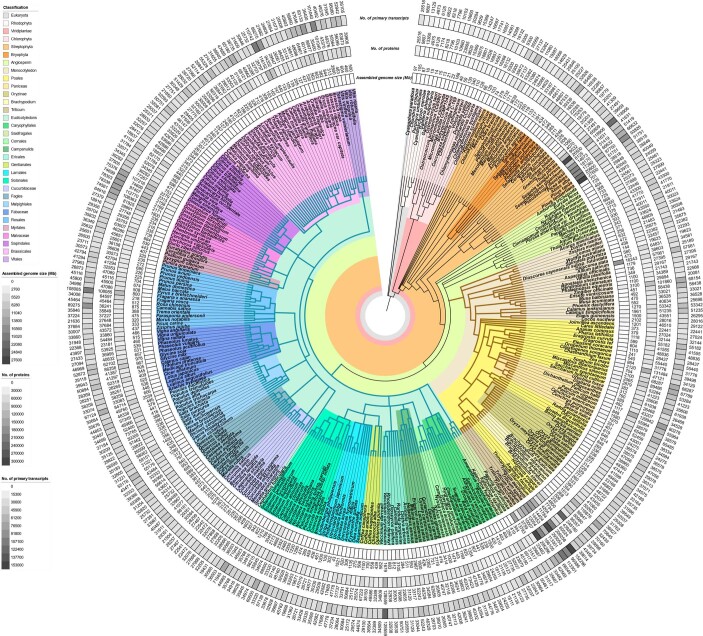
350 genomes used in this study. Phylogenetic tree of 350 plant species, with heatmaps representing the assembled genome size, number (no.) of annotated proteins and number (no.) of primary transcripts. Brown branches indicates monocots and teal branches represent eudicots.

**Extended Data Fig. 3:**
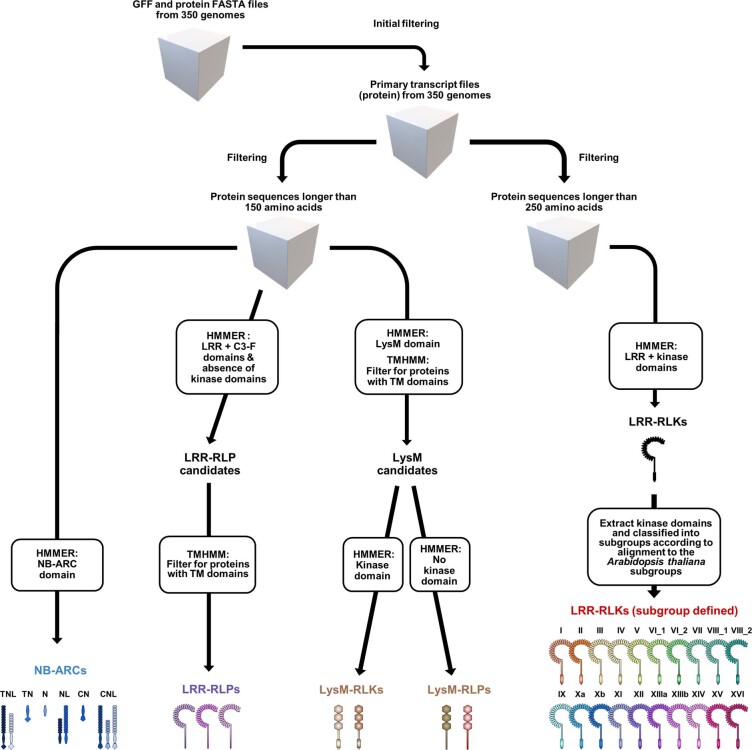
Identification of receptor gene families in 350 plant genomes. Protein sequences from all 350 proteomes were first filtered for the primary gene models. Primary-transcript proteomes were then filtered and NB-ARC, LRR-RLP, LysM-RLK, LysM-RLP and LRR-RLK proteins were identified. LRR-RLK genes were further classified into 20 subgroups according to their alignment to the *Arabidopsis thaliana* subgroups. Details of the pipeline are described in the methods section.

To investigate expansion or contraction of genes that encode PRR and NLR proteins, we identified these gene families in annotated proteomes from 350 publicly available genomes. These genomes include 26 algal species, 5 bryophyte species, 10 gymnosperms and 300 angiosperms (13 basal angiosperms, 79 monocots and 208 eudicots) (Extended Data Fig. 2 and Supplementary Table 1). Assembled genome sizes of these organisms range from 13 Mb to 27.6 Gb, with annotated protein counts ranging from ~5,000 to ~300,000 (Extended Data Fig. 2). To ensure consistency, we used the same pipeline to obtain primary transcripts and identify LRR-RLKs, LRR-RLPs, LysM-RLK, LysM-RLP and NB-ARCs from each of these genomes (Extended Data Fig. 3 and Methods).

**Fig. 1:**
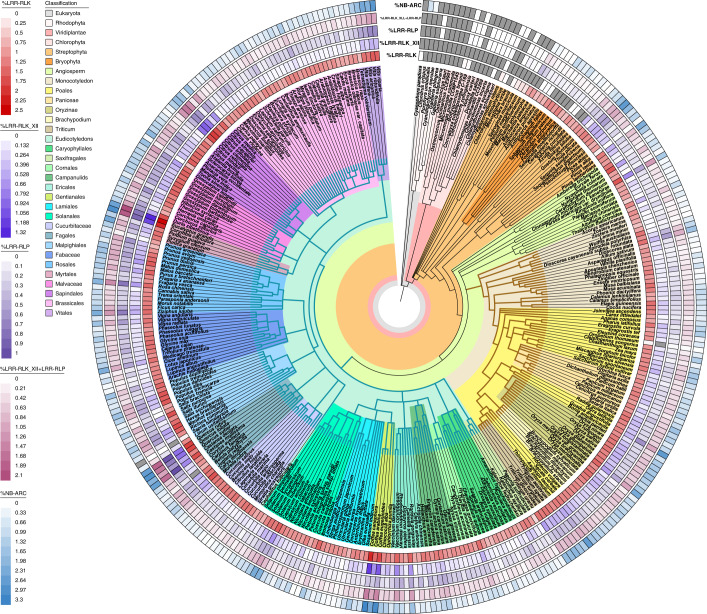
Immune receptor gene families in 350 genomes. Phylogenetic tree of 350 plant species, including 300 angiosperms, 79 monocot species and 208 eudicot species. Heat maps represent the percentages (%) of LRR-RLKs, LRR-RLK_XIIs (red), LRR-RLPs (purple), LRR-RLK-XIIs + LRR-RLPs (magenta) and NB-ARCs (blue) in their corresponding annotated proteomes. Grey boxes in heat maps indicate null values where no receptors were identified. Brown branches indicate monocots, and teal branches represent eudicots.

**Extended Data Fig. 4:**
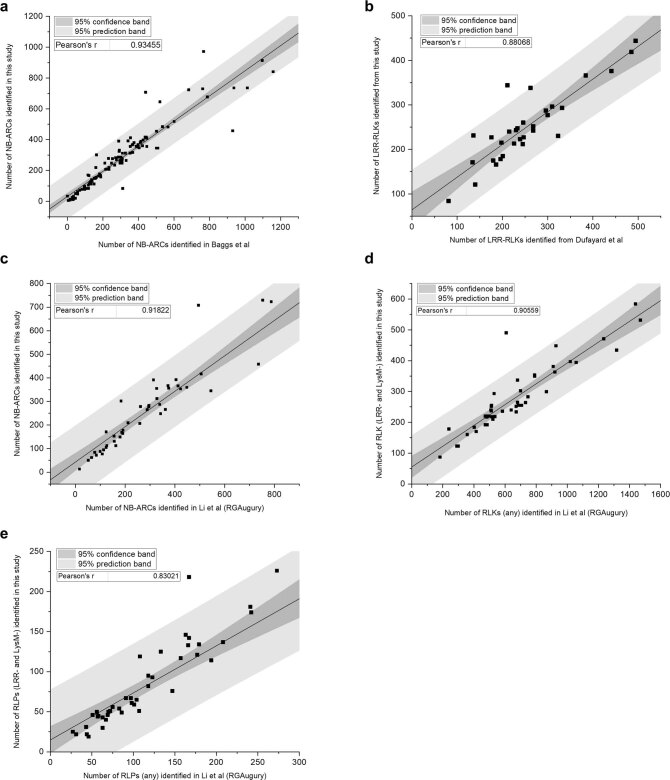
Validation of the methods used to identify receptor genes in this study. **a**, Scatter plot of number of NB-ARC genes identified in this study against number of NB-ARC genes identified in Baggs et al for the corresponding plant species^16^. **b**, Scatter plot of number of LRR-RLKs identified in this study against number of LRR-RLKs identified in Dufayard et al for the corresponding plant species^17^. **c**, Scatter plot of number of NB-ARC genes identified in this study against number of NB-ARC genes identified in Li et al (RGAugury database) for the corresponding plant species (ref). **d**, Scatter plot of number of RLK genes (LRR- and LysM-RLKs only) identified in this study against number of RLK genes (any RLKs, including LRR- and LysM-RLKs) identified in Li et al (RGAugury database) for the corresponding plant species^18^. **e**, Scatter plot of number of RLP genes (LRR- and LysM-RLPs only) identified in this study against number of RLP genes (any RLPs, including LRR- and LysM-RLPs) identified in Li et al (RGAugury database) for the corresponding plant species^18^. For each plot, the black line represents the linear trend, with dark grey shade represents the 95% confidence interval and light grey shade represents the 95% prediction interval.

In total, we identified 88,020 LRR-RLKs, 28,018 LRR-RLPs, 3,500 LysM-RLKs, 1,238 LysM-RLPs and 95,127 NB-ARCs from 350 species (Supplementary Fig. 1 and Supplementary Table 2). To validate our results, we compared the number of NB-ARCs, LRR-RLKs and LRR-RLPs identified in our study with previous publications, finding they are highly similar (Extended Data Fig. 4)^16–18^. As expected, the number of receptors varies enormously across 300 angiosperms, with LRR-RLKs ranging from 16 to 1,129, LRR-RLPs ranging from 2 to 585, LysM-RLKs ranging from 0 to 42, LysM-RLPs ranging from 0 to 19 and NB-ARCs ranging from 0 to 3,128. To account for the effect of genome duplication and variable proteome sizes, we normalized these data using percentages (%) of LRR-RLKs, LRR-RLPs, LysM-RLK, LysM-RLP and NB-ARCs from each genome (number of identified genes/number of searched genes × 100) (Supplementary Fig. 1 and Supplementary Table 3). After adjustment, LRR-RLKs range from 0.114% to 2.464%, LRR-RLPs range from 0.00652% to 1.010%, LysM-RLKs range from 0% to 0.120%, LysM-RLPs range from 0% to 0.0559% and NB-ARCs range from 0% to 3.266% in 300 angiosperms (Fig. 1, Supplementary Fig. 1 and Supplementary Table 3).

## Correlation between the sizes of immune receptor families

**Fig. 2:**
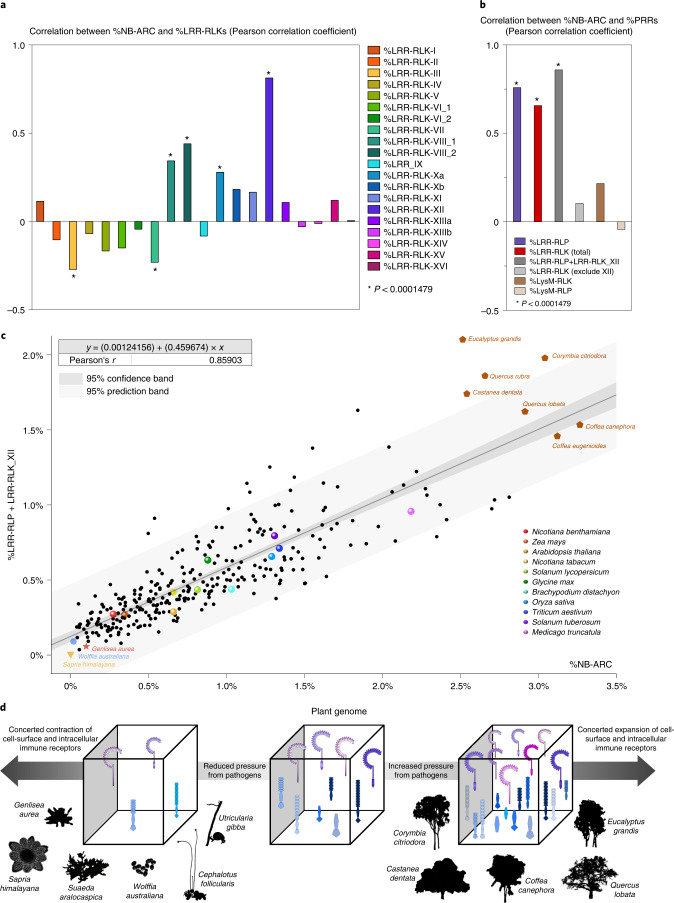
Concerted expansion and contraction of cell-surface and intracellular immune receptor genes in plant genomes. **a**,**b**, Correlation between %NB-ARC and %LRR-RLKs and %PRRs in 300 angiosperms. Bar chart represents the Pearson correlation coefficient, with significant values indicated with asterisks. Two-sided test of significance was performed, and Bonferroni correction was performed to adjust the *P* value for the multiple independent tests performed (Extended Data Fig. 6). Summary of statistical analyses and individual *P* values are provided in Supplementary Table 4. **c**, Scatter plot of %LRR-RLP + LRR-RLK-XII against %NB-ARC. Black line represents the linear trend, with dark-grey shade representing the 95% confidence interval and light-grey shade representing the 95% prediction interval. Several parasitic species, carnivorous species, aquatic species and trees are indicated as yellow inverted triangles, orange stars, blue circles and brown pentagons, respectively. Model organisms are also indicated as spheres of different colours. **d**, Schematic illustration of the co-expansion and co-contraction of immune receptors in plant genomes.

**Extended Data Fig. 5:**
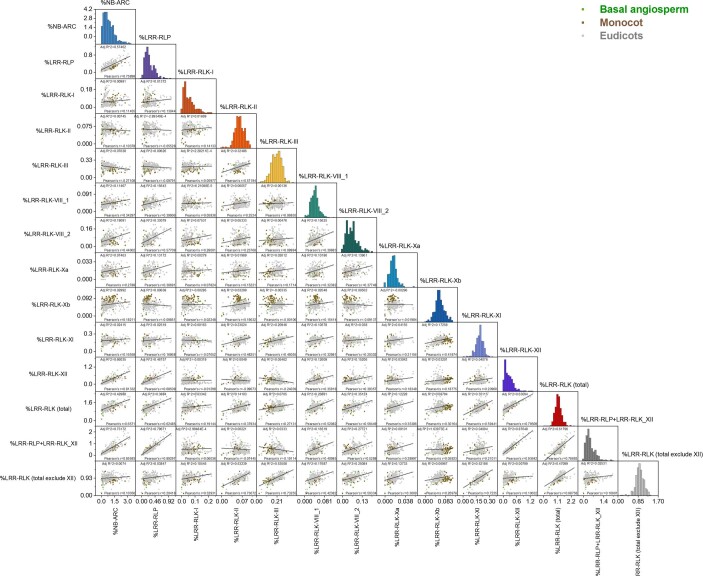
Scatter plot of Pearson correlation analysis between %NB-ARCs, %LRR-RLPs and %LRR-RLKs. Bottom left boxes include scatter plot between the corresponding % receptor-gene families in 300 angiosperms. Black line represents the linear trend. Grey dots represent basal angiosperms (n = 13), brown dots represent monocots (n = 79) and green dots represent eudiots (n = 208). The Pearson correlation coefficient (Pearson’s r) is indicated below each scatter plot. The diagonal boxes include the distribution of % receptor-gene families in 300 angiosperms.

**Extended Data Fig. 6:**
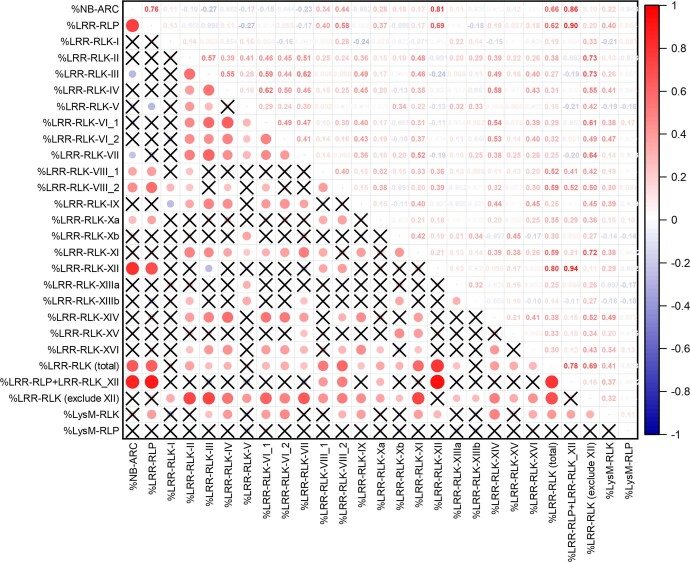
Pearson correlation plot of %NB-ARCs, %LRR-RLPs, %LRR-RLKs, %LysM-RLKs and %LysM-RLPs. Bottom left squares indicate the strength of correlation between the corresponding % receptor-gene families in 300 angiosperms. Two-sided test of significance was performed, and Bonferroni correction was performed to adjust the p-value for all the independent tests. The Bonferroni-corrected p-value at α = 0.05 would be 0.0001479. Thus, P-values < 0.0001479 are considered as significant after correction. Red circles indicate significant and positive linear correlations, blue circles indicate significant and negative linear correlations and crosses (X) indicate insignificant correlations. The sizes of circles represent the strength of the correlations. Top right squares indicate the values of Pearson correlation coefficient between the corresponding %receptor-gene families in 300 angiosperms. Red values represent positive correlations and blue values represent negative correlations. Exact p-values and summary of statistical analyses are provided in Supplementary Table 4.

Next, we determined the correlation between the percentages of PRRs (%LRR-RLKs, %LRR-RLPs, %LysM-RLK and %LysM-RLP) and NB-ARCs in angiosperms. Surprisingly, %NB-ARC and %LRR-RLPs show a strong positive linear correlation (Pearson’s *r* = 0.759), suggesting that NB-ARC and LRR-RLP gene families expand together (Fig. 2, Extended Data Figs. 5 and 6, Supplementary Fig. 7 and Supplementary Table 4). Similarly, %NB-ARC and %LRR-RLKs show a positive but weaker linear correlation (Pearson’s *r* = 0.657). On the other hand, %LysM-RLKs and %LysM-RLPs show weak or no correlation with %NB-ARC (Pearson’s *r* = 0.216 and −0.0430, respectively). We propose that PRRs involved in pathogen recognition are more likely to co-expand with NB-ARC gene families. This is consistent with the observation that characterized LRR-RLPs are usually involved in pathogen recognition, while LRR-RLKs and LysM receptors can be involved not only in immunity but also in development, reproduction or establishing symbiosis^12^.

To test if the NB-ARC gene family co-expands with PRRs specifically involved in pathogen recognition, we further classified LRR-RLKs into subgroups according to their kinase domains. As mentioned, LRR-RLKs can thus be classified into 20 subgroups, with each subgroup involved in various biological processes^10^ (Extended Data Fig. 1). Across 350 species, LRR-RLK-XII forms the largest LRR-RLK subgroup, followed by LRR-RLK-III and LRR-RLK-XI (Supplementary Figs. 2 and 3). We determined the correlation between %LRR-RLK from different subgroups and %NB-ARC in angiosperms (Extended Data Figs. 5 and 6, and Supplementary Table 4). Strikingly, only 4 out of 20 LRR-RLK subgroups show significant and positive linear correlation with %NB-ARCs (LRR-RLK-VIII_1, LRR-RLK-VIII_2, LRR-RLK-Xa and LRR-RLK-XII). Furthermore, LRR-RLK-XII forms much stronger positive correlation with %NB-ARCs (Pearson’s *r* = 0.813) compared with LRR-RLK-VIII_1, LRR-RLK-VIII_2 and LRR-RLK-Xa (Pearson’s *r* = 0.343, 0.440 and 0.279, respectively) (Fig. 2a, Supplementary Fig. 7 and Supplementary Table 4). While LRR-RLKs involved in pathogen recognition are predominantly in subgroup XII, some members from LRR-RLK-VIII and LRR-RLK-Xa are also involved in immunity and pathogen recognition, such as CANNOT RESPOND TO DMBQ 1 (CARD1/HPCA1), CELLOOLIGOMER-RECEPTOR KINASE 1 (CORK1) and BAK1-INTERACTING RECEPTOR-LIKE KINASE 1 (BIR1) (refs. ^19–22^) (Extended Data Fig. 1). As LRR-RLK-XII forms the largest LRR-RLK subgroup, we tested if the positive correlation between %LRR-RLK (total) and %NB-ARC is predominantly caused by subgroup XII. Indeed, for all subgroups excluding XII, %LRR-RLK does not show any significant correlation with %NB-ARC (Pearson’s *r* = 0.103). On the other hand, %LRR-RLP combined with %LRR-RLK-XII show strong positive correlation with %NB-ARC (Pearson’s *r* = 0.859) (Fig. 2a,b).

**Extended Data Fig. 7:**
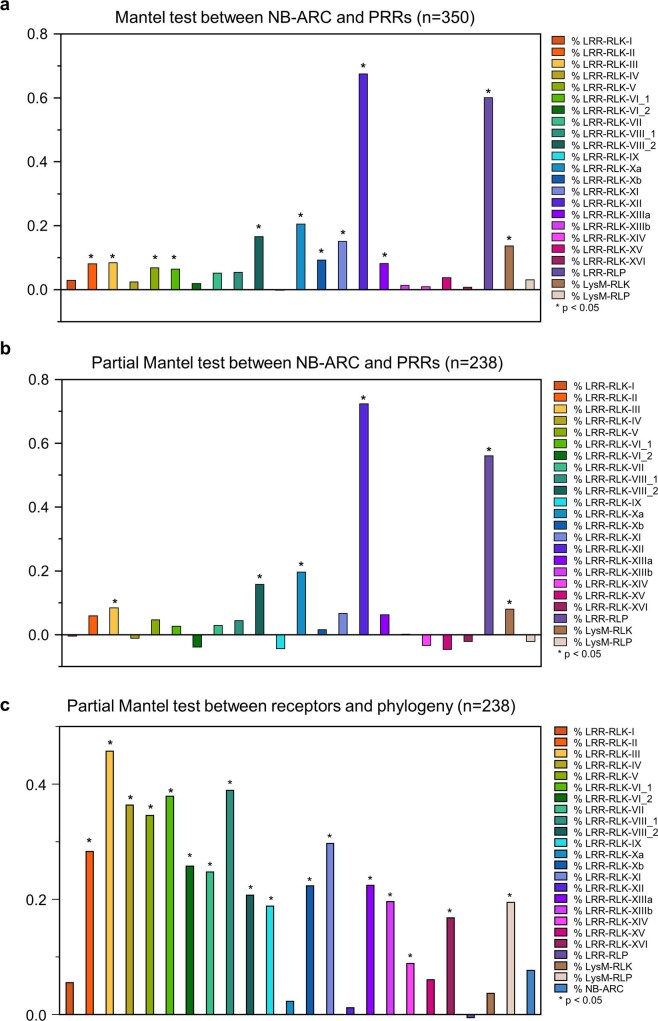
Test for similarities in fraction of receptor proteins and phylogenetic relationships. To test whether the receptor percentage (%) found per species correlate with phylogenetic relationship, %receptor and the species phylogenetic tree were converted to distance matrices and tested for correlation with the Mantel tests. **a**, Mantel test for the correlation between distance matrices obtained for % receptor family proteins in 350 species. Histogram shows the correlation coefficient between % PRRs and % NB-ARCs. **b**, Partial Mantel test for the correlation between distance matrices obtained for % receptor in 238 species, taking phylogenetic distances into account. Histogram shows the correlation coefficient between % PRRs and % NB-ARCs. **c**, Mantel test for the correlation between distance matrices obtained for %receptors and phylogenic distances in 238 species. Histogram shows the correlation coefficient between %receptor and phylogeny. One-sided test of significance was performed. P-values were corrected for multiple testing to reflect false discovery rates (FDRs, for details please refer to methods). Summary of statistical analyses are provided in Supplementary Table 4.

We further tested the hypothesis taking into account the phylogeny of the plant species. First, we converted receptor percentages from 350 genomes into distance matrices and tested for correlation between receptor pairs with Mantel tests. %LRR-RLK-XII and %LRR-RLP show strong positive correlation with %NB-ARC (Extended Data Fig. 7a). Second, we obtained a phylogenetic tree from a previous publication^23^ for 238 species and tested whether percentage receptor distances correlate with each other while taking into account the phylogenetic distances with a partial Mantel test. Again, %LRR-RLK-XII and %LRR-RLP show strong positive correlation with %NB-ARC (Extended Data Fig. 7b). Third, we tested for correlation between receptor percentages and phylogenetic distances directly. Whereas almost all %LRR-RLKs significantly correlate with the phylogeny, %LRR-RLK-XII, %LRR-RLPs and %NB-ARC do not (Extended Data Fig. 7c). Taken together, we conclude that PRR gene families specifically involved in pathogen recognition co-expand or co-contract with NB-ARC gene families.

## Expansion and contraction of immune receptor families

**Extended Data Fig. 8:**
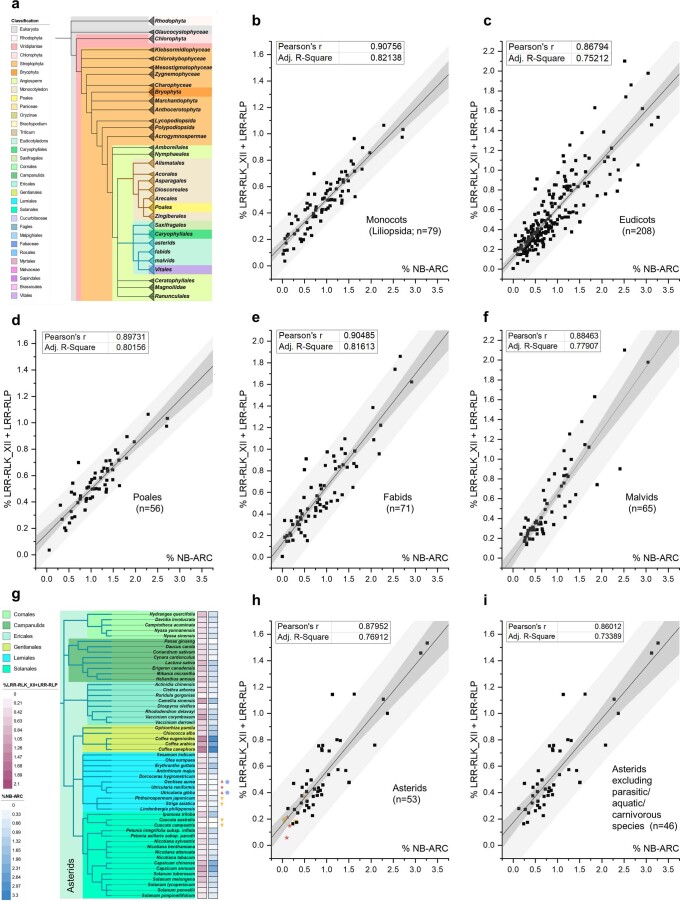
Correlation between %PRRs and %NLRs in plant clades. **a**, Phylogenetic tree of the species used in the analysis. Pearson correlation between %LRR-RLK_XII+LRR-RLP and %NB-ARC in **b**, Monocots, **c**, Eudicots, **d**, Poales, **e**, Fabids, **f**, Malvids and **h**, Asterids. **g**, Phylogenetic tree of the Asterids clade used in the analysis. Carnivorous plants are marked with orange stars; parasitic plants are marked with yellow triangles and aquatic plants are marked with blue circles. **i**, Pearson correlation between %LRR-RLK_XII+LRR-RLP and %NB-ARC in the Asterids clade, excluding carnivorous, parasitic, and aquatic plants.

**Extended Data Fig. 9:**
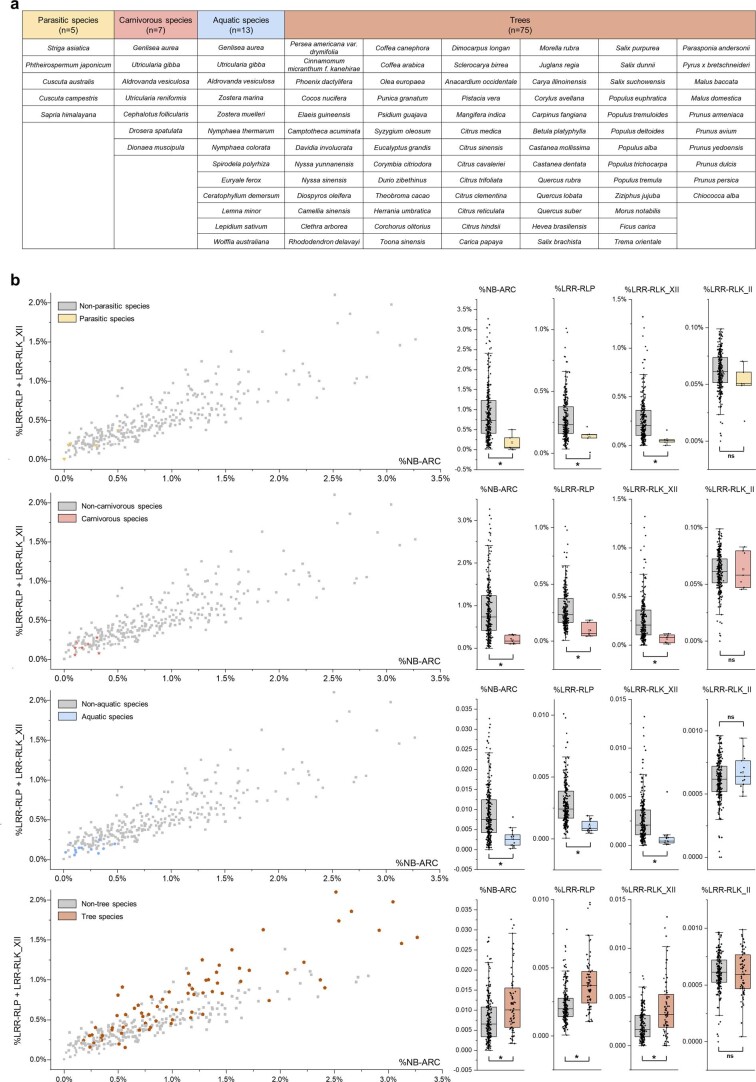
Expansion and contraction of PRR- and NLR-gene families in plants adapted to particular lifestyles and ecological niches. **a**, List of parasitic species, carnivorous species, aquatic species and trees from this study. **b**, (left) Scatter plot of % LRR-RLP+LRR-RLK-XII against % NB-ARC in 300 angiosperms. Parasitic, carnivorous, aquatic and trees species are highlighted as yellow inverted triangles, orange stars, blue circles and brown pentagons, respectively. (right) Box-plot of % NB-ARC, LRR-RLP, LRR-RLK-XII and LRR-RLK-II in parasitic (yellow), carnivorous (orange), aquatic (blue) and trees species (brown) compared to other plant species (gray). Box-plot elements: centre line, median; bounds of box, 25th and 75th percentiles; whiskers, 1.5 × IQR from 25th and 75th percentiles. A two-sided Welch’s t-test was used to analyze significant differences between the groups (*P ≤ 0.05; ns, not significant). Exact P-values are provided in Supplementary Table 4. Number of species (n) in each category: non-parasitic species, n = 295; parasitic species, n = 5; non-carnivorous species, n = 293; carnivorous species, n = 7; non-aquatic species, n = 287; aquatic species, n = 13; tree species, n = 225; non-tree species, n = 75.

We observed a strong linear correlation between %NB-ARC and %LRR-RLK-XII and %LRR-RLP in angiosperms, monocots, eudicots and multiple plant clades (Extended Data Fig. 8). Next, we checked if NB-ARC gene family contraction coincides with PRR gene family contraction in organisms adapted to specific lifestyles, such as parasitism and carnivorism. The Alismatales and Lentibulariaceae lineages show a reduction in the size of NB-ARC gene repertoires^16^, and species from these lineages also have low %PRRs (%LRR-RLP, %LRR-RLK-XII and %LysM-RLK). These include *Genislea aurea*, *Utricularia gibba*, *Utricularia reniformis*, *Zostera marina*, *Zostera muelleri*, *Lemna minor*, *Wolffia australiana* and *Spirodela polyrhiza* (Fig. 2c, Extended Data Fig. 9, Supplementary Table 3 and Supplementary Fig. 6). We infer that the %NB-ARC and %LRR-RLK correlation is not due just to co-expansion, but also co-contraction. Carnivorous, aquatic and parasitic plant genomes carry few NLRs^16,24^. We tested if the number of cell-surface immune receptors is also reduced in these plants. Compared with species that are not adapted to these lifestyles, carnivorous, aquatic and parasitic plant genomes have lower %NB-ARC, %LRR-RLK-XII and %LRR-RLP (Extended Data Fig. 9 and Supplementary Fig. 6). These include *Sapria himalayana*, *Cephalotus follicularis*, *Drosera spatulata*, *Dionaea muscipula* and *Aldrovanda vesiculosa*. Notably, %LRR-RLK (total) in these groups is similar to other plant species, as are most other LRR-RLK subgroups (Supplementary Fig. 6).

Some other species and genera also show lower %NB-ARC, %LRR-RLK and %LRR-RLP. For example, the Cucurbitaceae show far fewer immune receptors than the phylogenetically close Malpighiales or Fagales clades (Fig. 1, Supplementary Fig. 4 and Supplementary Table 3). Remarkably, in the monocot species *Oropetium thomaceum*, we observed only 0.0558% NB-ARC containing proteins and no LRR-RLK-XII. This contrasts with the other members of the Poales, where high %PRRs and %NB-ARCs are more frequent (Fig. 1 and Supplementary Table 3). *O. thomaeum* is an atypical member in the Poales. This drought-tolerant resurrection grass has the smallest known grass genome (245 Mb) and can survive losing 95% of cellular water^25^. Despite its small genome, *O. thomaeum* has a similar number of predicted proteins as other Poales species such as *Ananas comosus*, *Oryza longistaminata* and *Triticum urartu*, suggesting that the contraction of immune receptor families could be independent of the reduced genome size.

On the other hand, some plant groups show much larger immune receptor families. Many species of the order Poales show high %LRR-RLK, %LRR-RLP and %NB-ARC, most notably in the *Oryza* and *Triticum* genera (Fig. 1 and Supplementary Fig. 4). In addition, many tree species also show a high proportion of PRR and NB-ARC proteins in their proteomes. These include *Eucalyptus grandis*, *Castanea dentata*, *Corymbia citriodora*, *Quercus rubra*, *Quercus lobata*, *Coffea canephora*, *Prunus avium*, *Malus domestica*, *Theobroma cacao* and *Citrus* species (Fig. 2c, Extended Data Fig. 9 and Supplementary Table 3). Thus, some plant lifestyles might also correlate with expansion of immune receptor gene families.

**Extended Data Fig. 10:**
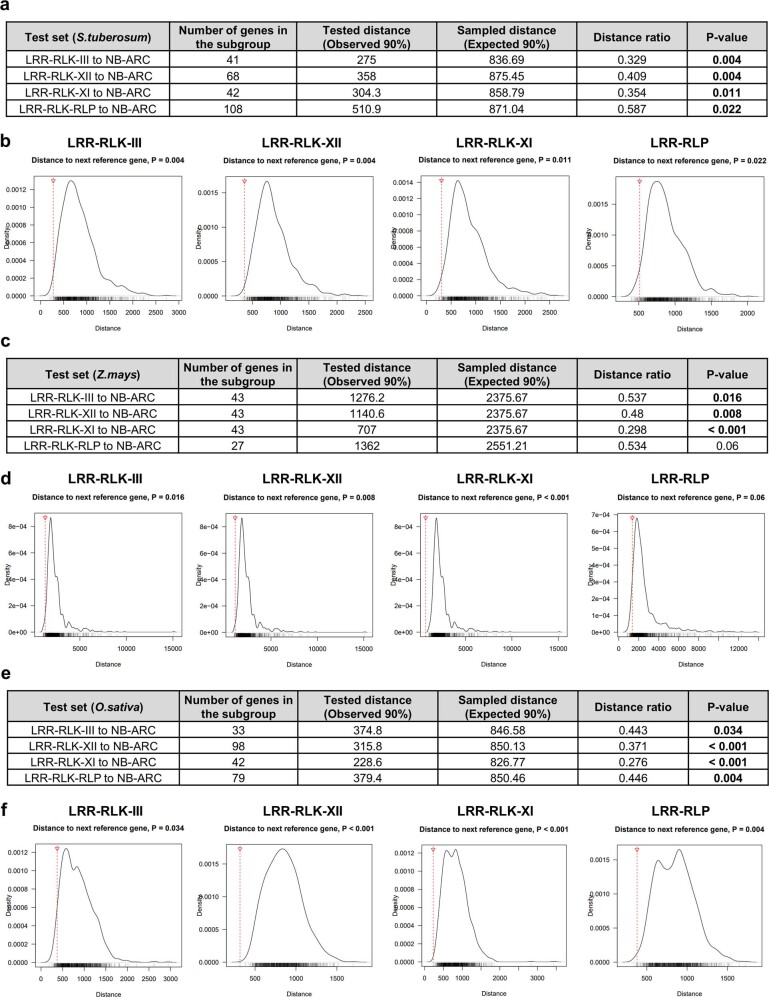
Genomic clustering of LRR-RLKs, LRR-RLPs and NB-ARCs in *Solanum tuberosum*, *Zea mays* and *Oryza sativa*. **a, c, e**. Table summarizing the statistical analysis of genomic clustering between PRRs (LRR-RLKs and LRR-RLPs) and NB-ARCs in *Solanum tuberosum* (a), *Zea mays* (c) and *Oryza sativa* (e). The 90-percentile distance (bp) between PRR gene family members and the next closest NB-ARC genes were calculated. This is then compared to a distribution (n = 1000) of 90-percentile distances between randomly-sampled genes and the next closest NB-ARC genes. One-sided test was performed to test the differences between tested distance (PRRs) and sampled distance (randomly-sampled). P-values are calculated based on the comparison to 1000 cases of randomly-sampled genes. Significant values are indicated in bold (p-value < 0.05 is considered as significant). **b, d, f**. Distribution (n = 1000) of 90-percentile distances (bp) between randomly-sampled genes and the next closest NB-ARC genes in *Solanum tuberosum* (b), *Zea mays* (d) and *Oryza sativa* (f). Red lines indicate the 90-percentile distance between the corresponding PRR gene family members and the next closest NB-ARC genes.

Previously, analysis of the *Solanum lycopersicum* genome has suggested that NLRs, RLPs and RLKs might form genomic clusters^26^. Genomic clustering could mean that expansion/contraction of a gene family could result in genes in close proximity indirectly expanding in tandem. To determine if concerted expansion/contraction of immune receptor families is due to genomic clustering, we investigated the *Solanum tuberosum*, *Zea mays* and *Oryza sativa* genomes. In all three genomes, many LRR-RLK-XII and LRR-RLP loci overlap with NB-ARC-encoding loci (Supplementary Fig. 7). To quantify this, we calculated the average distance of LRR-RLKs and LRR-RLPs to the closest NB-ARC encoding genes and compared with a distribution of randomly selected genes (for details, see Methods). Both LRR-RLK-XIIs and LRR-RLPs are located closer to NB-ARC genes than randomly selected genes. However, LRR-RLK-III and LRR-RLK-XI genes are also located nearby to NB-ARC genes (Extended Data Fig. 10). As %LRR-RLK-III and %LRR-RLK-XI do not show positive correlation with %NB-ARC, we conclude that, while NB-ARC-encoding genes can form genomic clusters adjacent to LRR-RLK-XIIs, the co-expansion/contraction of these immune receptors is likely to be caused by mechanisms other than genomic clustering.

## Discussion

Previously it was shown that cell-surface and intracellular immune systems exhibit mutual potentiation and inter-dependency^3–6^. Here we show that, in addition to their functional relationship, there is also an evolutionary correlation between the numbers of cell-surface and intracellular immune receptors. Expansion and/or contraction of intracellular NLRs coincides with expansion and/or contraction of cell-surface PRRs involved in pathogen recognition (Fig. 2d). These observations are consistent with previous reports^16,24,27,28^.

We propose that pathogen pressure shapes the immune receptor diversity and repertoire, which, as a result, is determined by plant lifestyles and their ecological niches. We observed high %PRR and %NB-ARC in many *Oryza* and *Triticum* species. Grasses typically grow in high densities and are frequently challenged by rust and blast species that produce numerous, wind-dispersed spores with high genetic diversity. Genetic exchange by sexual reproduction and somatic hybridization drives the emergence of new virulent strains^29^, such as the *Ug99* strain of the wheat stem rust pathogen *Puccinia graminis* f. sp. *tritici*^30^. An expanded repertoire of immune receptors and increased heterogeneity in populations could be a result of high pressure from these pathogens. Conversely, it has been proposed that the reduced root system in parasitic and carnivorous plants results in fewer interactions or entry routes for pathogens^24^. Similarly, partial or complete submersion of aquatic species results in reduced exposure to airborne pathogenic spores, removing an interface for interaction with pathogens. Lifespan may also drive changes in the immune receptor repertoire. We found that trees generally show higher %PRR and %NB-ARC than other species. While annual plants are subject to shorter periods of pathogen pressure before reproduction, biennial or perennial plants, especially trees, must survive for much longer. Conceivably, this long-term pathogen pressure could drive the expansion of immune receptor gene families.

As parasite pressure drives the retention of sexual reproduction that reshuffles immune receptor alleles each generation^31^, inbreeding species may require an increased number of immune receptors compared with their outbreeding ancestors, an outcome that can also result from polyploidy. As the concerted expansion and contraction of immune receptors in plant genomes is not due to genomic clustering, further study is needed to understand the mechanism(s) underpinning these observations. As functionally inter-dependent genes often co-expand/contract together, it is likely that the functional relationship between cell-surface and intracellular immune receptors is conserved across plant species.

## Methods

### LRR-RLK identification

Protein sequences from all 350 plant proteomes were first filtered for the primary gene model. Sequences shorter than 250 amino acids (AA) were removed as they are unlikely LRR-RLKs. The remaining proteins were searched for the presence of a protein kinase domain (PFAM PF00069.26) and an LRR domain (PFAM PF18805.2, PF18831.2, PF18837.2, PF00560.34, PF07723.14, PF07725.13, PF12799.8, PF13306.7, PF13516.7, PF13855.7, PF14580.7, PF01463.25, PF08263.13 and PF01462.19) with hmmer (version 3.1b2, options -E 1e-10 for the kinase domain and -E 10e-3 for the LRR domains^32^). The *Arabidopsis* sequences that were previously classified into 20 LRR-RLK subgroups^10^ were filtered likewise for the presence of LRR and kinase domains. Eleven sequences were removed because they did not pass the threshold filter for the kinase (two sequences) and LRR (nine sequences) domain searches. To classify all candidate sequences according to the *Arabidopsis* subgroups, the highest-scoring kinase domain region of each candidate was extracted and aligned to the *Arabidopsis* reference sequences using diamond^33^ (version 0.9.26, options -e 1e-10 -k 300).

### Phylogeny

The phylogeny of each subgroup was inferred using the kinase domains. Sequences were aligned with FAMSA^34^. Alignments were not trimmed^35^ and phylogenetic trees were inferred with FastTree^36^ (version 2.1.11 SSE3, option -lg). Trees were rooted with gotree^37^ (v0.4.2) using the sequences belonging to the most basal species as outgroup (according to the taxonomic tree from National Center for Biotechnology Information (NCBI)).

### LRR-RLP identification

LRR-RLPs were identified similarly but filtering for proteins of a minimal length of 150 AA first. Proteins were then searched for the presence of LRR domains and the absence of a kinase domain (hmmer options as above), as well as the presence of a C3F domain (hmmer option -E 1e-10 and a minimal alignment length of 140). The hmmer profile for the C3F domain was obtained from a multiple alignment of *Arabidopsis* LRR-RLPs^38^. The domain was trimmed manually, starting from the conserved Y in the C2 domain (Fig. 6b in ref. ^39^). Candidates were finally filtered for the presence of a transmembrane domain using tmhmm^40^ with default settings (version 2.0).

### NB-ARC identification

NB-ARCs were identified using the set of proteins with a minimal length of 150 AA. Proteins were then searched for the presence of NB-ARC (PF00931.23) domains (hmmer option -E 1e-10 for NB-ARC).

### LysM identification

LysM-RLKs and LysM-RLPs were identified using the set of proteins with a minimal length of 150 AA. Proteins were filtered for the presence of a LysM domain (PF01476.21, hmmer option –max -E 1000–incE 1000–incdomE 1000) and a transmembrane domain^40^ (tmhmm, version 2.0). Candidates were split into LysM-RLKs and LysM-RLPs by searching for presence/absence of a kinase domain (PF00069.26, as above).

### Test for co-occurrence of NB-ARC, LRR-RLKs and LRR-RLPs

To test whether two gene groups are closer to each other than expected by chance, we used a test based on random sampling, for example, group A (LRR-RLK-XII) with *n* and group B (NB-ARCs) with *m* genes. The observed distance was calculated as the average closest distance between genes in group A and genes in group B. A distribution for the expected distance was obtained by randomly sampling *m* genes and calculating the average closest distance of genes in group A to the genes in the random set (1,000 times). Genes were sampled from the list of genes that was used to search for the genes in group B (Supplementary Fig. 1).

### Taxonomic tree

The taxonomic tree was obtained from NCBI (https://www.ncbi.nlm.nih.gov/Taxonomy/CommonTree/wwwcmt.cgi). Phylogenetic tree of the 350 species is generated by phyloT (https://phylot.biobyte.de/) based on NCBI taxonomy database. Phylogenetics trees were visualized and figures were generated by iTOL^41^. The tree used for testing the relationship between the fraction of candidates found and the phylogenetic distances were obtained from ref. ^23^. The latter contained 238 out of the 350 genomes analysed.

### Test for similarities in fraction of proteins and phylogenetic relationship

To test whether the fraction of certain proteins (for example, NB-ARCs) found per species correlated with the phylogenetic relationships, we converted the fractions and the phylogenetic tree to distance matrices and tested for correlation with mantel tests (R package vegan, version 2.5-7 with 10,000 permutations). Analogously, we also tested for correlation between distance matrices obtained for two different sets of proteins (for example, LRR-RLK-XII and NB-ARCs). *P* values were corrected for multiple testing to reflect false discovery rates^42^.

### Statistical analyses

Statistical analyses were performed with OriginPro (version 2022; https://www.originlab.com/) and R (version 3.4.4).

### Reporting summary

Further information on research design is available in the Nature Research Reporting Summary linked to this article.

## Supplementary information

Supplementary Information Supplementary Figs. 1–7.

Reporting Summary

Supplementary Tables Supplementary Table 1. List of species included in this study. Supplementary 2. Number of receptor genes in each species. Supplementary Table 3. Percentage of receptor genes in each species. Supplementary 4. Statistical analyses in this study.

## Data Availability

All data generated or analysed during this study are included in the article or supplementary information files. Proteomes of 350 species used in this study are downloaded from either NCBI, Phyozome13, ensemblplants, JGI, Fernbase, Penium Genome Database or the publications directly. A complete list of the proteomes and associated data used in this study are provided in Supplementary Table 1. Sequences of the identified receptors and phylogenetic analyses are available on Zenodo^43^.

## Code availability

All the analyses were done as described in Methods with publicly available tools (hmmer, tmhmm, diamond, FastTree, FAMSA, gotree and R). Scripts are available on github.com/MWSchmid/Ngou-et-al.-2022.
