# Supplementary material for: Concerted expansion and contraction of immune receptor gene repertoires in plant genomes
Source: Nat Plants. 2022 Oct 14;8(10):1146–52. doi: 10.1038/s41477-022-01260-5 (PMC9579050; doi:10.1038/s41477-022-01260-5)
Supplement: Supplementary file 1 — Supplementary Figs. 1–7. [file 41477_2022_1260_MOESM1_ESM.pdf]

nature plants

Brief Communication https://doi.org/10.1038/s41477-022-01260-5

Concerted expansion and contraction of immune receptor gene repertoires in plant genomes

In the format provided by the authors and unedited

a

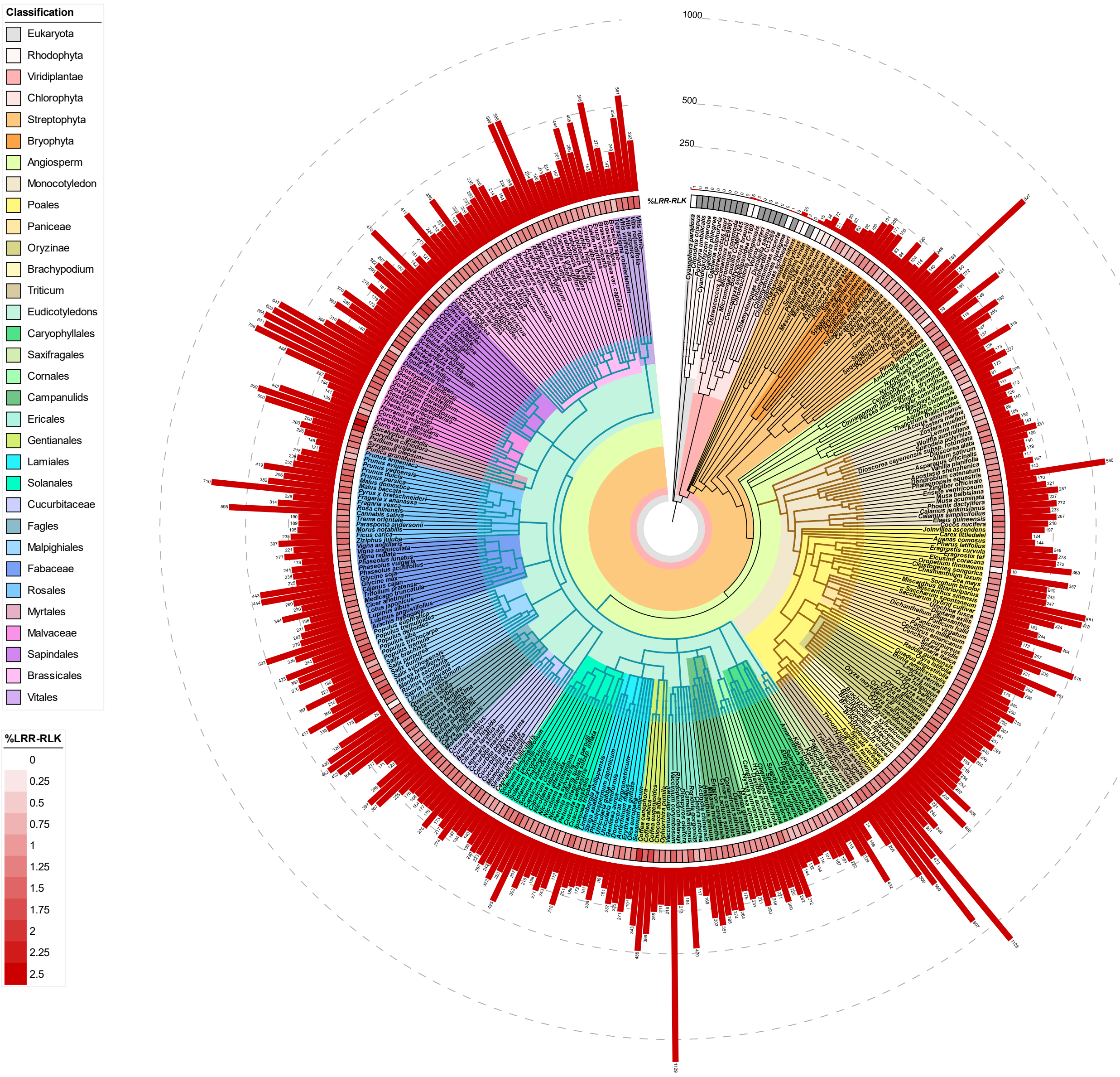
Classification
Eukaryota
Rhodophyta
Viridiplantae
Chlorophyta
Streptophyta
Bryophyta
Angiosperm
Monocotyledon
Poales
Paniceae
Oryzinae
Brachypodium
Triticum
Eudicotyledons
Caryophyllales
Saxifragales
Cornales
Campanulids
Ericales
Gentianales
Lamiales
Solanales
Cucurbitaceae
Fagles
Malpighiales
Fabaceae
Rosales
Myrtales
Malvaceae
Sapindales
Brassicales
Vitales
%LRR-RLK
0
0.25
0.5
0.75
1
1.25
1.5
1.75
2
2.25
2.5
1000
500
250
%LRR-RLK

b

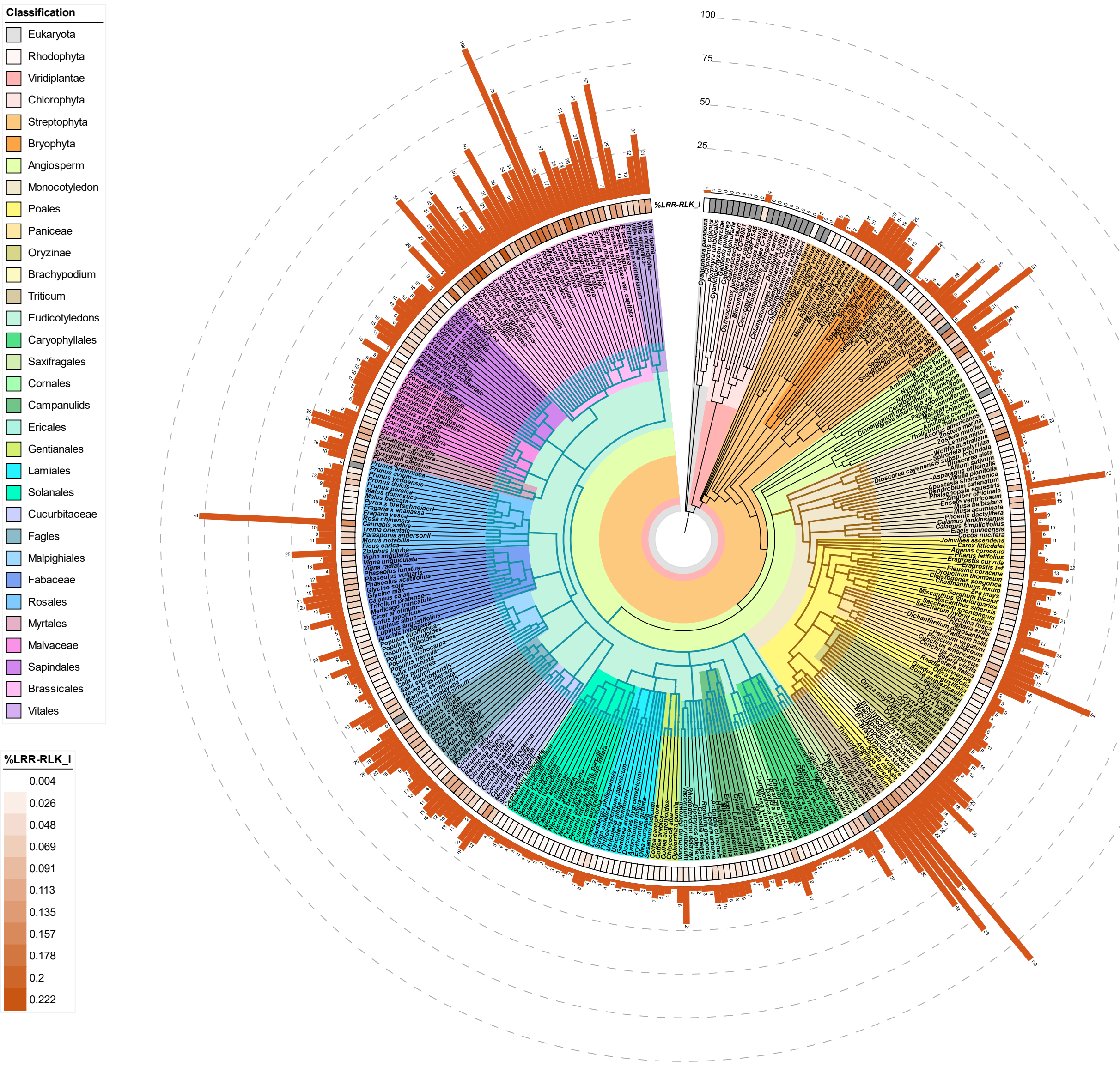
Classification
Eukaryota
Rhodophyta
Viridiplantae
Chlorophyta
Streptophyta
Bryophyta
Angiosperm
Monocotyledon
Poales
Paniceae
Oryzinae
Brachypodium
Triticum
Eudicotyledons
Caryophyllales
Saxifragales
Cornales
Campanulids
Ericales
Gentianales
Lamiales
Solanales
Cucurbitaceae
Fagles
Malpighiales
Fabaceae
Rosales
Myrtales
Malvaceae
Sapindales
Brassicales
Vitales
%LRR-RLK_I
0.004
0.026
0.048
0.069
0.091
0.113
0.135
0.157
0.178
0.2
0.222
100
75
50
25
%LRR-RLK_I

c

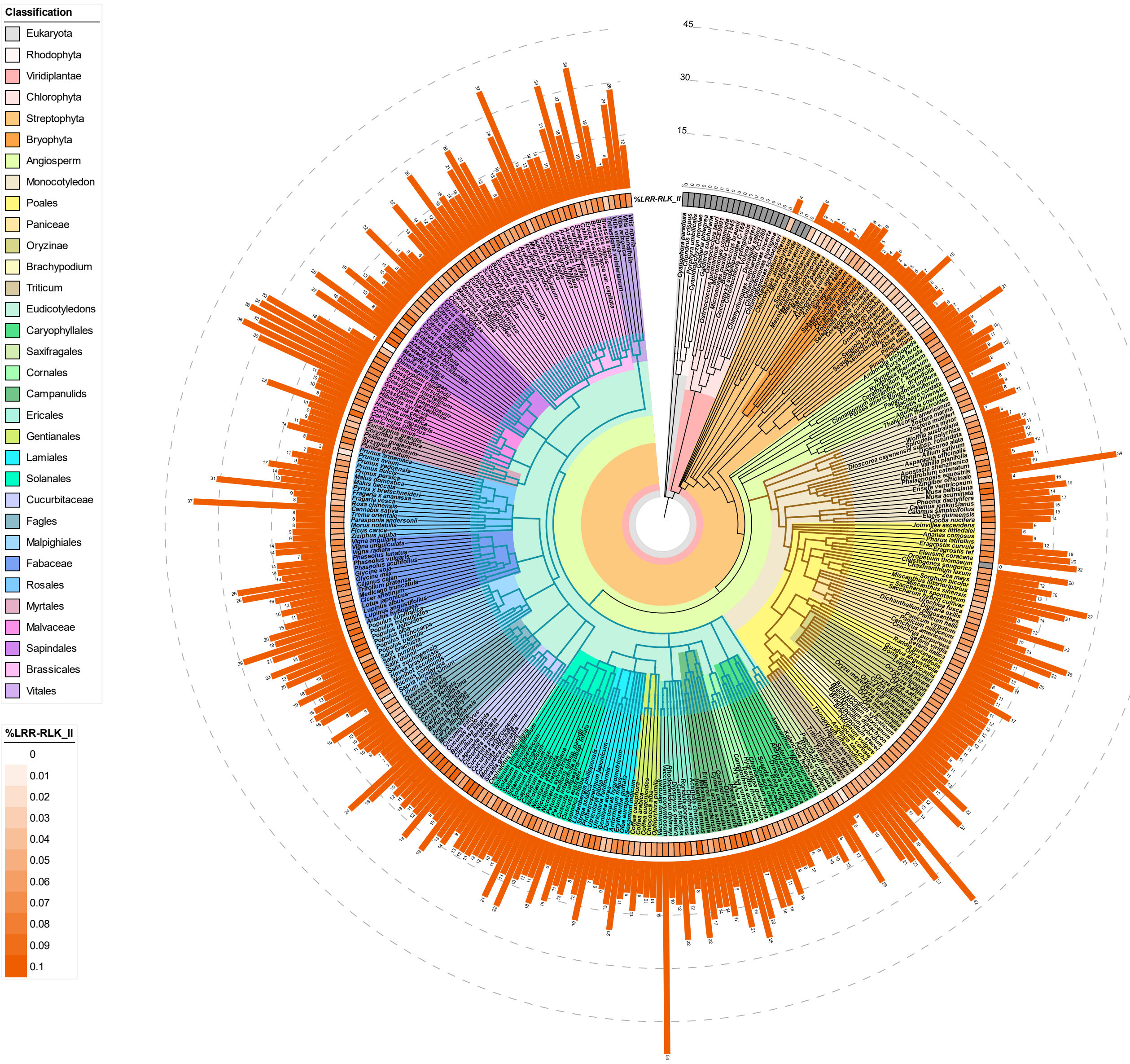
Classification
Eukaryota
Rhodophyta
Viridiplantae
Chlorophyta
Streptophyta
Bryophyta
Angiosperm
Monocotyledon
Poales
Paniceae
Oryzinae
Brachypodium
Triticum
Eudicotyledons
Caryophyllales
Saxifragales
Cornales
Campanulids
Ericales
Gentianales
Lamiales
Solanales
Cucurbitaceae
Fagles
Malpighiales
Fabaceae
Rosales
Myrtales
Malvaceae
Sapindales
Brassicales
Vitales
%LRR-RLK_II
0
0.01
0.02
0.03
0.04
0.05
0.06
0.07
0.08
0.09
0.1
45
30
15
%LRR-RLK_II

d

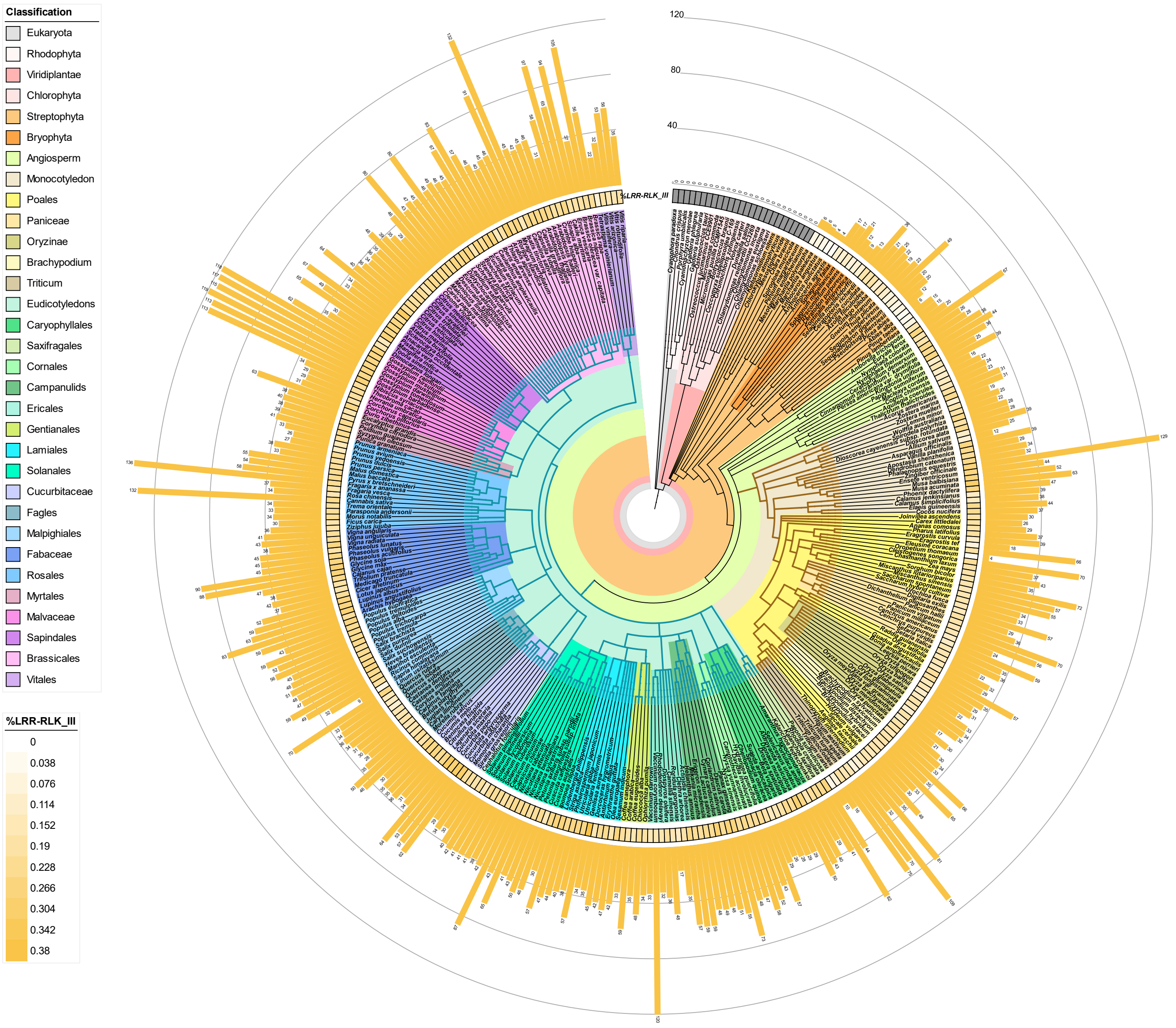
Classification
Eukaryota
Rhodophyta
Viridiplantae
Chlorophyta
Streptophyta
Bryophyta
Angiosperm
Monocotyledon
Poales
Paniceae
Oryzinae
Brachypodium
Triticum
Eudicotyledons
Caryophyllales
Saxifragales
Cornales
Campanulids
Ericales
Gentianales
Lamiales
Solanales
Cucurbitaceae
Fagles
Malpighiales
Fabaceae
Rosales
Myrtales
Malvaceae
Sapindales
Brassicales
Vitales
%LRR-RLK_III
0
0.038
0.076
0.114
0.152
0.19
0.228
0.266
0.304
0.342
0.38
120
80
40
%LRR-RLK_III

e

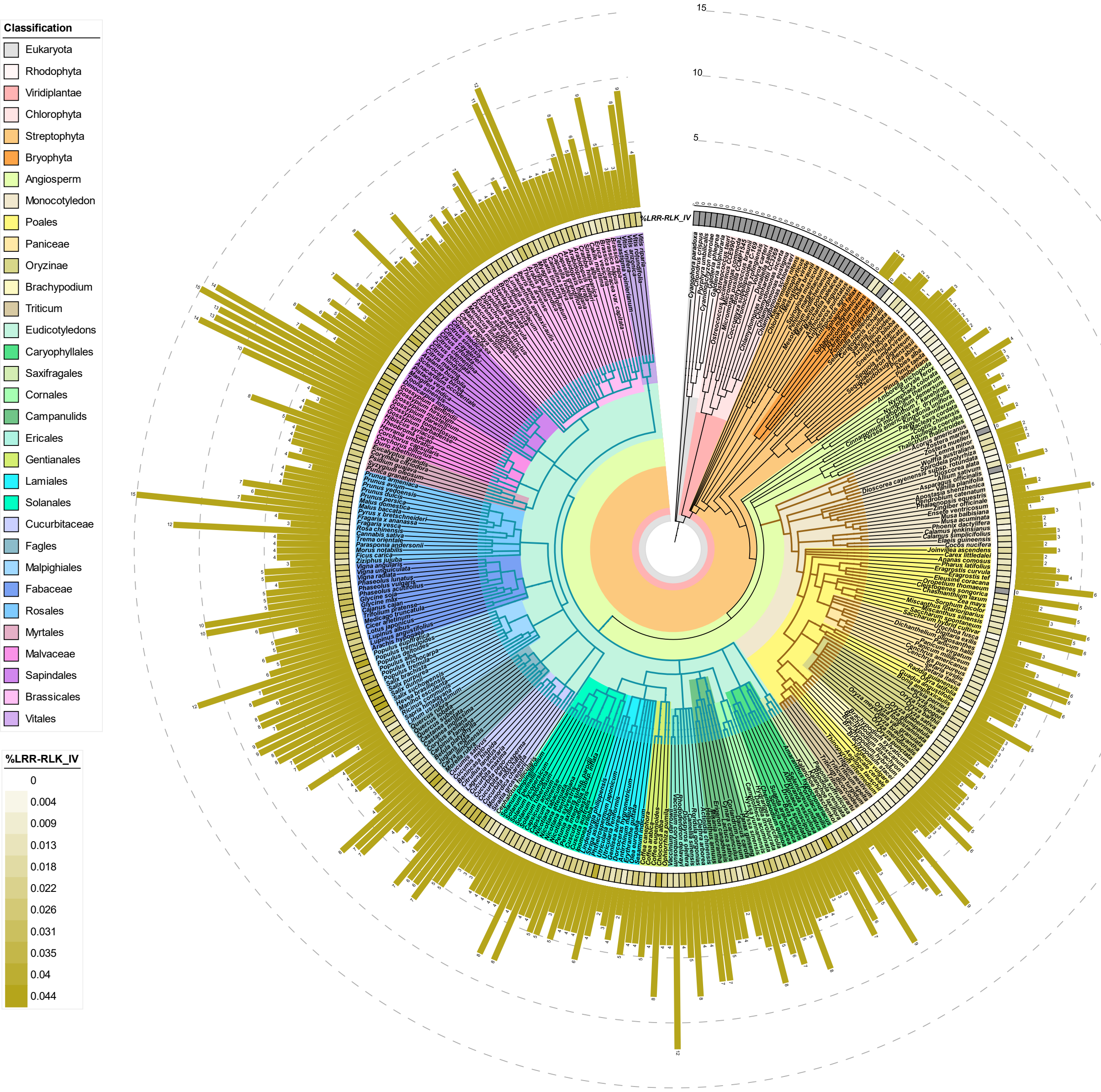
Classification
Eukaryota
Rhodophyta
Viridiplantae
Chlorophyta
Streptophyta
Bryophyta
Angiosperm
Monocotyledon
Poales
Paniceae
Oryzinae
Brachypodium
Triticum
Eudicotyledons
Caryophyllales
Saxifragales
Cornales
Campanulids
Ericales
Gentianales
Lamiales
Solanales
Cucurbitaceae
Fagles
Malpighiales
Fabaceae
Rosales
Myrtales
Malvaceae
Sapindales
Brassicales
Vitales
%LRR-RLK_IV
0
0.004
0.009
0.013
0.018
0.022
0.026
0.031
0.035
0.04
0.044
15
10
5
%LRR-RLK_IV

f

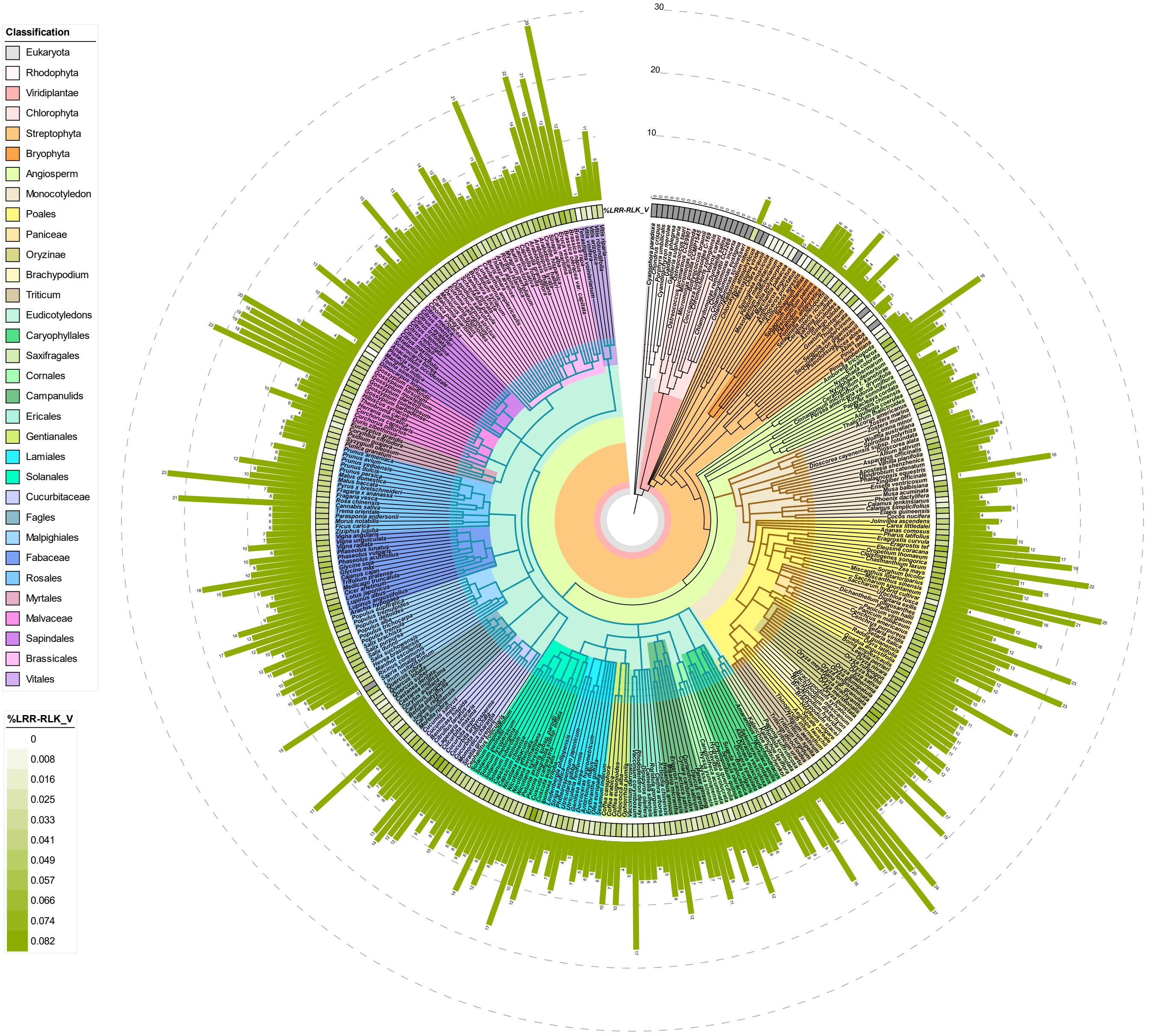
Classification
Eukaryota
Rhodophyta
Viridiplantae
Chlorophyta
Streptophyta
Bryophyta
Angiosperm
Monocotyledon
Poales
Paniceae
Oryzinae
Brachypodium
Triticum
Eudicotyledons
Caryophyllales
Saxifragales
Cornales
Campanulids
Ericales
Gentianales
Lamiales
Solanales
Cucurbitaceae
Fagles
Malpighiales
Fabaceae
Rosales
Myrtales
Malvaceae
Sapindales
Brassicales
Vitales
%LRR-RLK_V
0
0.008
0.016
0.025
0.033
0.041
0.049
0.057
0.066
0.074
0.082
30
20
10
%LRR-RLK_V

g

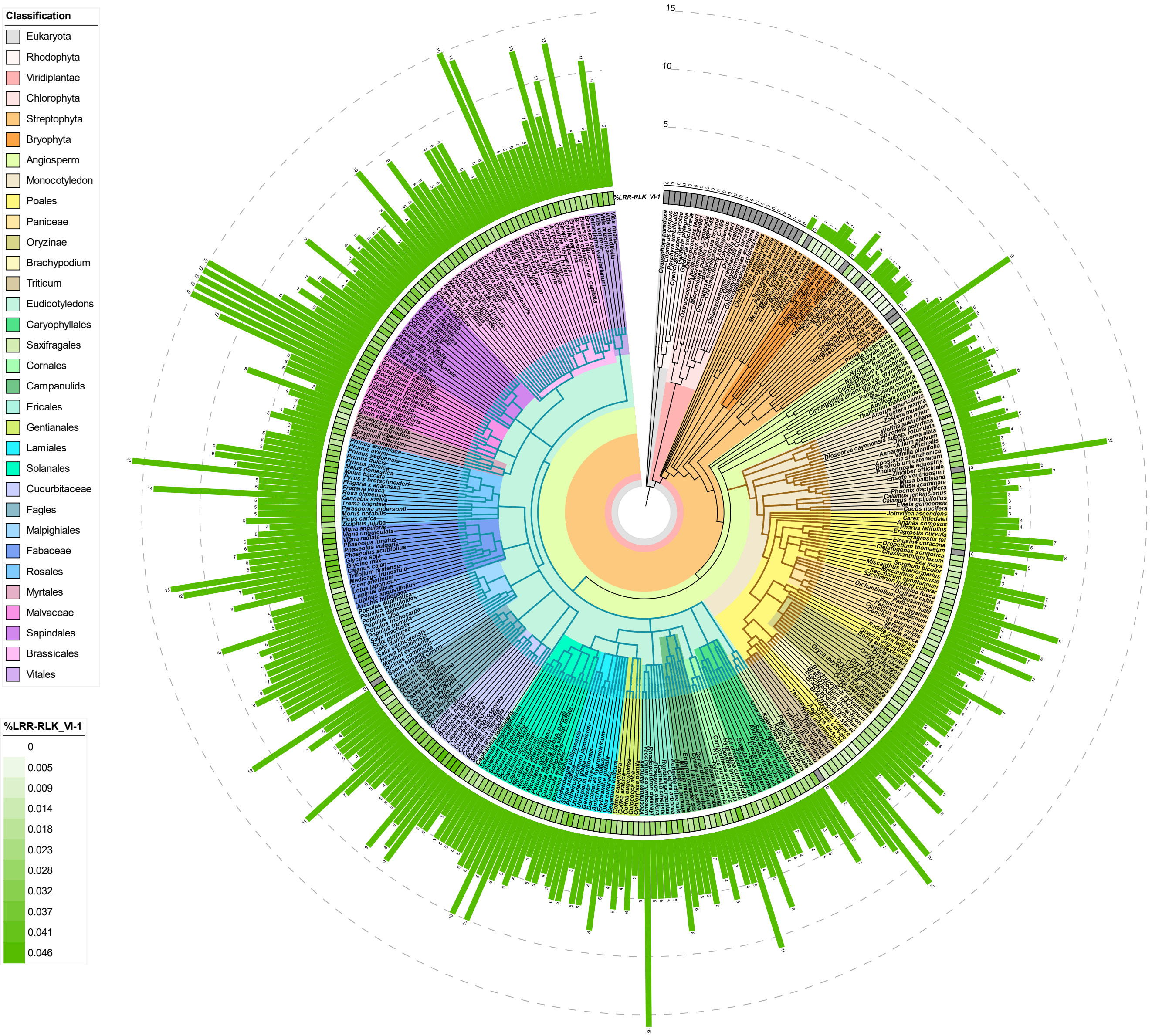
Classification
Eukaryota
Rhodophyta
Viridiplantae
Chlorophyta
Streptophyta
Bryophyta
Angiosperm
Monocotyledon
Poales
Paniceae
Oryzinae
Brachypodium
Triticum
Eudicotyledons
Caryophyllales
Saxifragales
Cornales
Campanulids
Ericales
Gentianales
Lamiales
Solanales
Cucurbitaceae
Fagles
Malpighiales
Fabaceae
Rosales
Myrtales
Malvaceae
Sapindales
Brassicales
Vitales
%LRR-RLK_VI-1
0
0.005
0.009
0.014
0.018
0.023
0.028
0.032
0.037
0.041
0.046
15
10
5

h

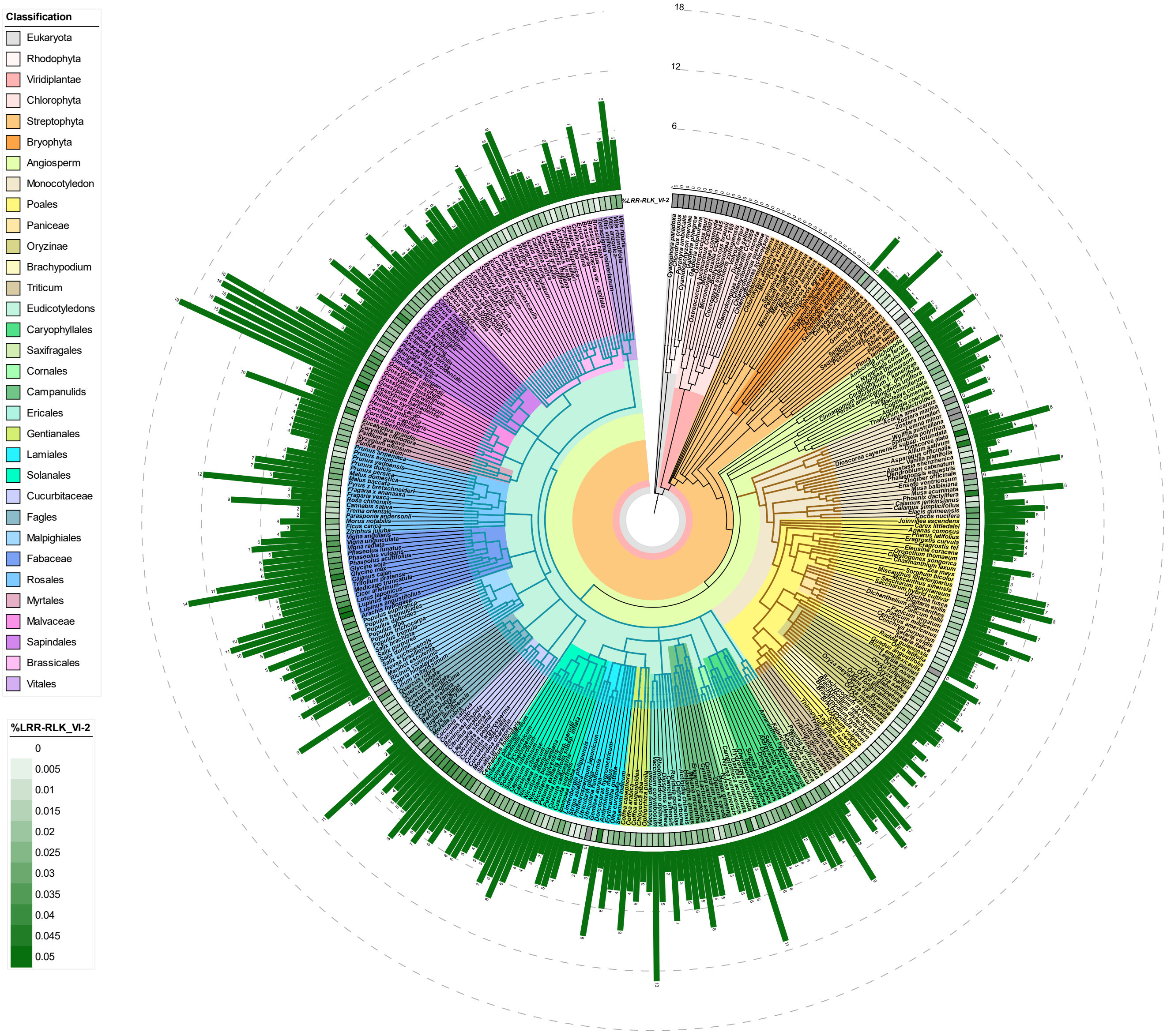
Classification
Eukaryota
Rhodophyta
Viridiplantae
Chlorophyta
Streptophyta
Bryophyta
Angiosperm
Monocotyledon
Poales
Paniceae
Oryzinae
Brachypodium
Triticum
Eudicotyledons
Caryophyllales
Saxifragales
Cornales
Campanulids
Ericales
Gentianales
Lamiales
Solanales
Cucurbitaceae
Fagles
Malpighiales
Fabaceae
Rosales
Myrtales
Malvaceae
Sapindales
Brassicales
Vitales
%LRR-RLK_VI-2
0
0.005
0.01
0.015
0.02
0.025
0.03
0.035
0.04
0.045
0.05
18
12
6
%LRR-RLK_VI-2

i

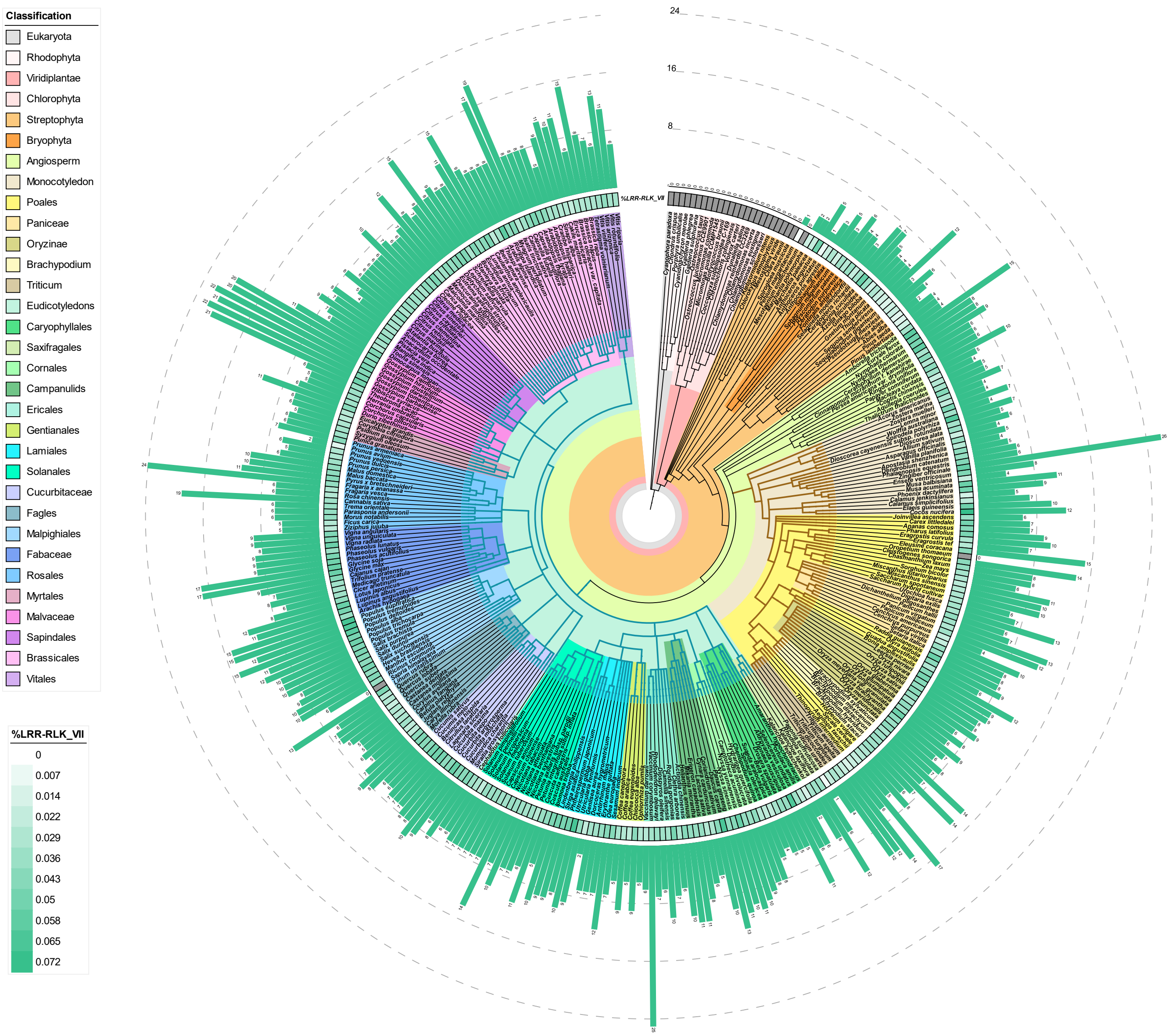
Classification
Eukaryota
Rhodophyta
Viridiplantae
Chlorophyta
Streptophyta
Bryophyta
Angiosperm
Monocotyledon
Poales
Paniceae
Oryzinae
Brachypodium
Triticum
Eudicotyledons
Caryophyllales
Saxifragales
Cornales
Campanulids
Ericales
Gentianales
Lamiales
Solanales
Cucurbitaceae
Fagles
Malpighiales
Fabaceae
Rosales
Myrtales
Malvaceae
Sapindales
Brassicales
Vitales
%LRR-RLK_VII
0
0.007
0.014
0.022
0.029
0.036
0.043
0.05
0.058
0.065
0.072
24
16
8
%LRR-RLK_VII

j

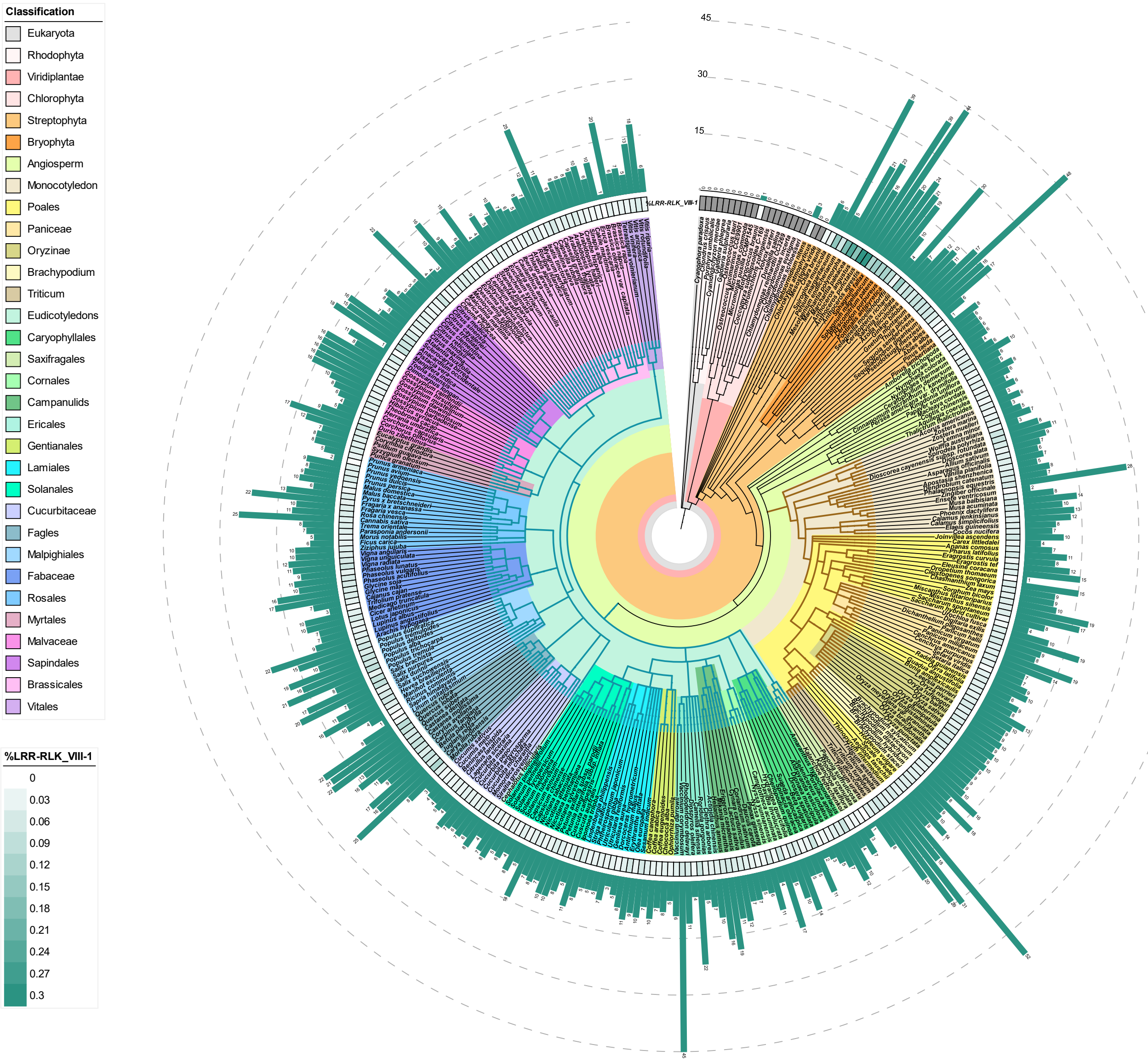
Classification
Eukaryota
Rhodophyta
Viridiplantae
Chlorophyta
Streptophyta
Bryophyta
Angiosperm
Monocotyledon
Poales
Paniceae
Oryzinae
Brachypodium
Triticum
Eudicotyledons
Caryophyllales
Saxifragales
Cornales
Campanulids
Ericales
Gentianales
Lamiales
Solanales
Cucurbitaceae
Fagles
Malpighiales
Fabaceae
Rosales
Myrtales
Malvaceae
Sapindales
Brassicales
Vitales
%LRR-RLK_VIII-1
0
0.03
0.06
0.09
0.12
0.15
0.18
0.21
0.24
0.27
0.3
45
30
15

k

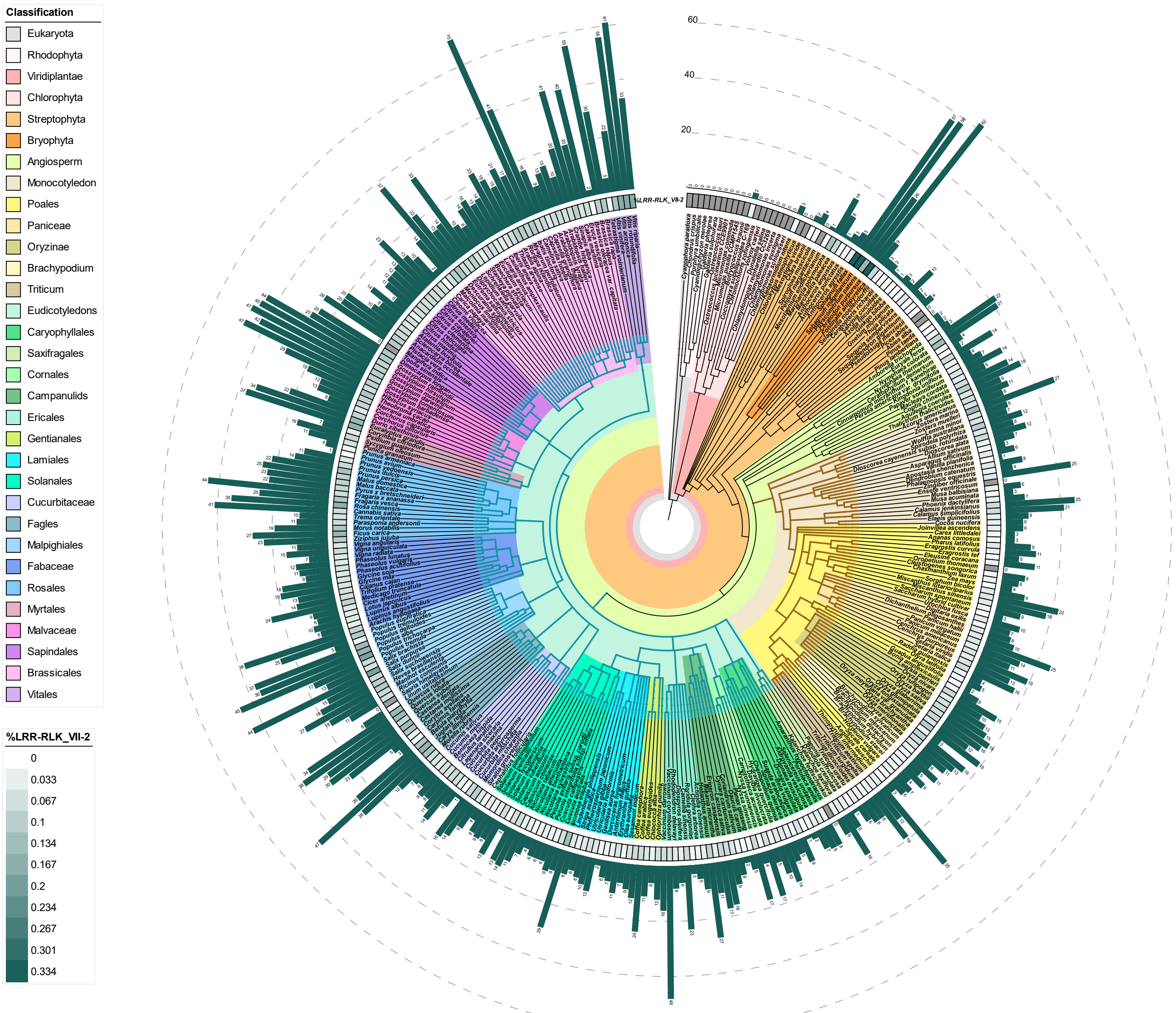
Classification
Eukaryota
Rhodophyta
Viridiplantae
Chlorophyta
Streptophyta
Bryophyta
Angiosperm
Monocotyledon
Poales
Paniceae
Oryzinae
Brachypodium
Triticum
Eudicotyledons
Caryophyllales
Saxifragales
Cornales
Campanulids
Ericales
Gentianales
Lamiales
Solanales
Cucurbitaceae
Fagles
Malpighiales
Fabaceae
Rosales
Myrtales
Malvaceae
Sapindales
Brassicales
Vitales
%LRR-RLK_VII-2
0
0.033
0.067
0.1
0.134
0.167
0.2
0.234
0.267
0.301
0.334
60
40
20

I

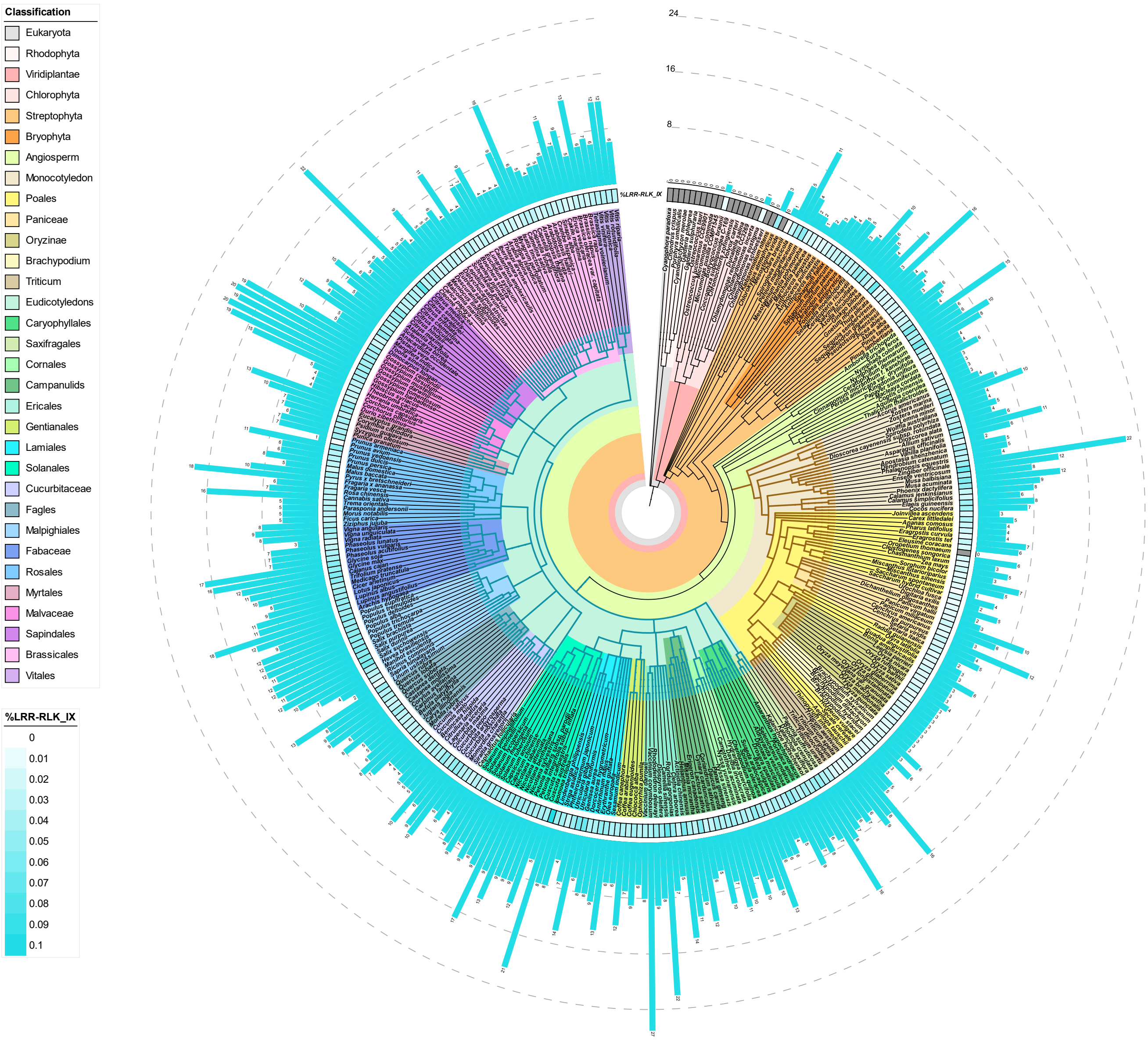
Classification
Eukaryota
Rhodophyta
Viridiplantae
Chlorophyta
Streptophyta
Bryophyta
Angiosperm
Monocotyledon
Poales
Paniceae
Oryzinae
Brachypodium
Triticum
Eudicotyledons
Caryophyllales
Saxifragales
Cornales
Campanulids
Ericales
Gentianales
Lamiales
Solanales
Cucurbitaceae
Fagles
Malpighiales
Fabaceae
Rosales
Myrtales
Malvaceae
Sapindales
Brassicales
Vitales
%LRR-RLK_IX
0
0.01
0.02
0.03
0.04
0.05
0.06
0.07
0.08
0.09
0.1
24
16
8
%LRR-RLK_IX

m

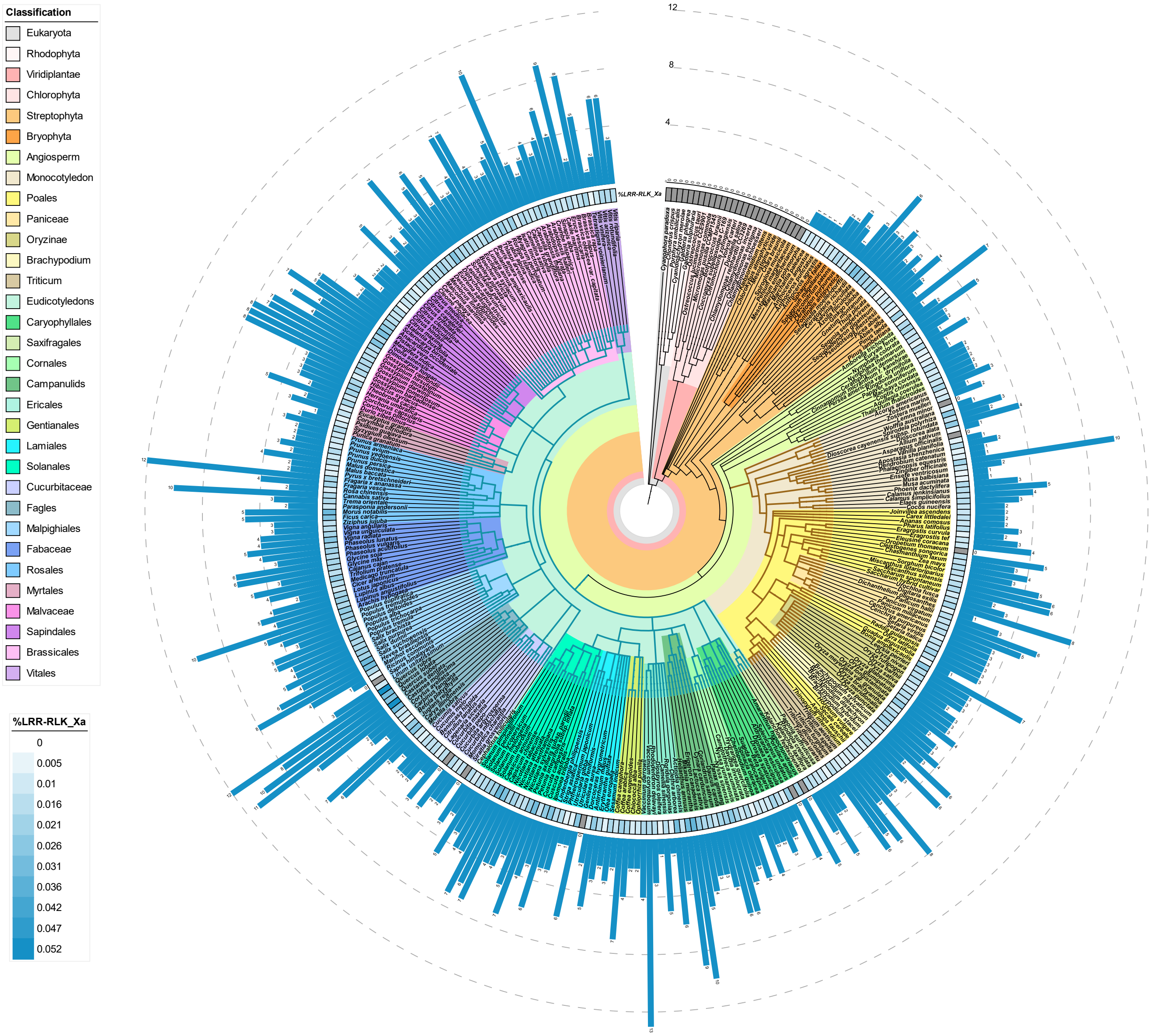
Classification
Eukaryota
Rhodophyta
Viridiplantae
Chlorophyta
Streptophyta
Bryophyta
Angiosperm
Monocotyledon
Poales
Paniceae
Oryzinae
Brachypodium
Triticum
Eudicotyledons
Caryophyllales
Saxifragales
Cornales
Campanulids
Ericales
Gentianales
Lamiales
Solanales
Cucurbitaceae
Fagles
Malpighiales
Fabaceae
Rosales
Myrtales
Malvaceae
Sapindales
Brassicales
Vitales
%LRR-RLK_Xa
0
0.005
0.01
0.016
0.021
0.026
0.031
0.036
0.042
0.047
0.052
12
8
4

n

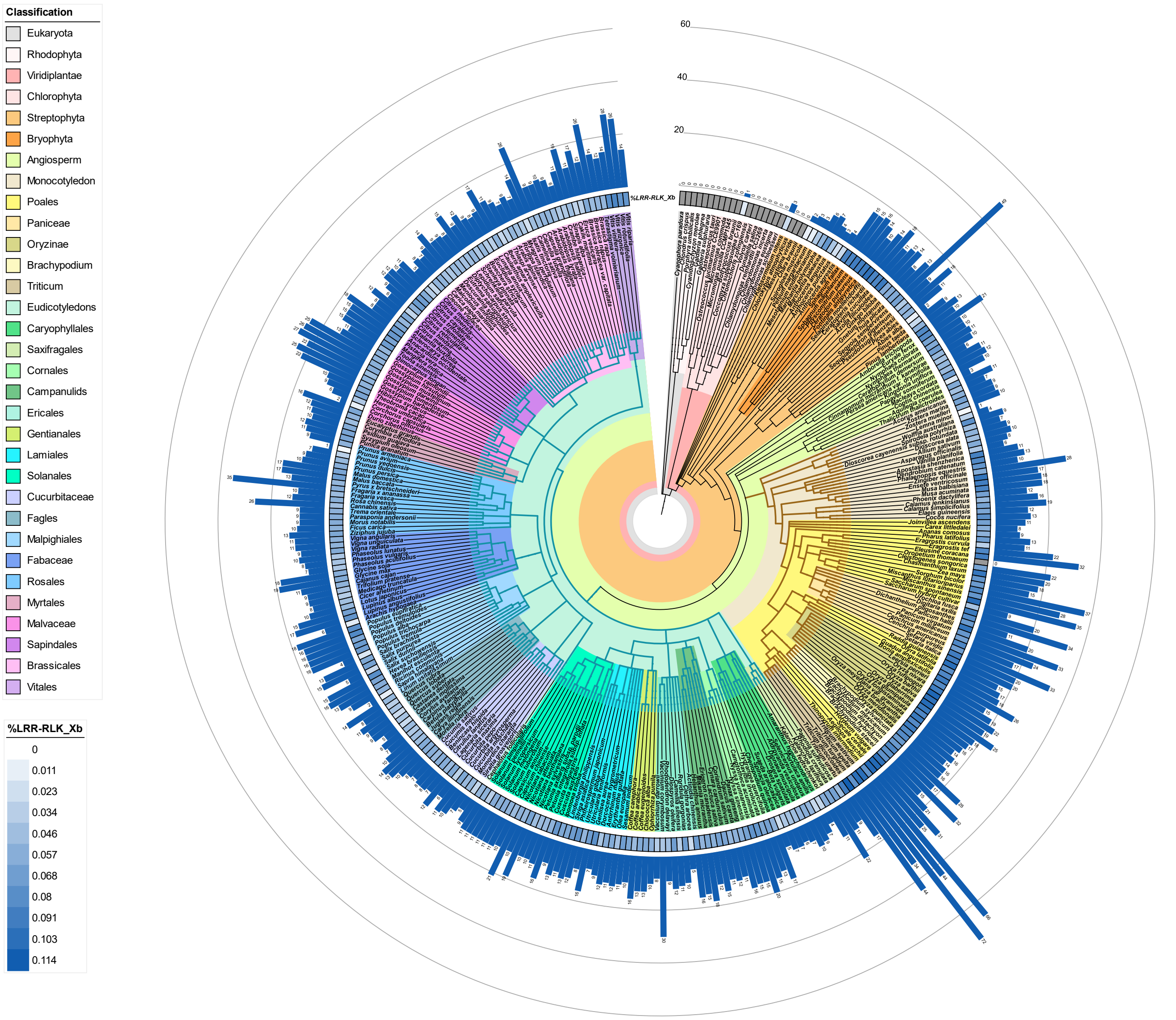
Classification
Eukaryota
Rhodophyta
Viridiplantae
Chlorophyta
Streptophyta
Bryophyta
Angiosperm
Monocotyledon
Poales
Paniceae
Oryzinae
Brachypodium
Triticum
Eudicotyledons
Caryophyllales
Saxifragales
Cornales
Campanulids
Ericales
Gentianales
Lamiales
Solanales
Cucurbitaceae
Fagles
Malpighiales
Fabaceae
Rosales
Myrtales
Malvaceae
Sapindales
Brassicales
Vitales
%LRR-RLK_Xb
0
0.011
0.023
0.034
0.046
0.057
0.068
0.08
0.091
0.103
0.114
60
40
20
%LRR-RLK_Xb

o

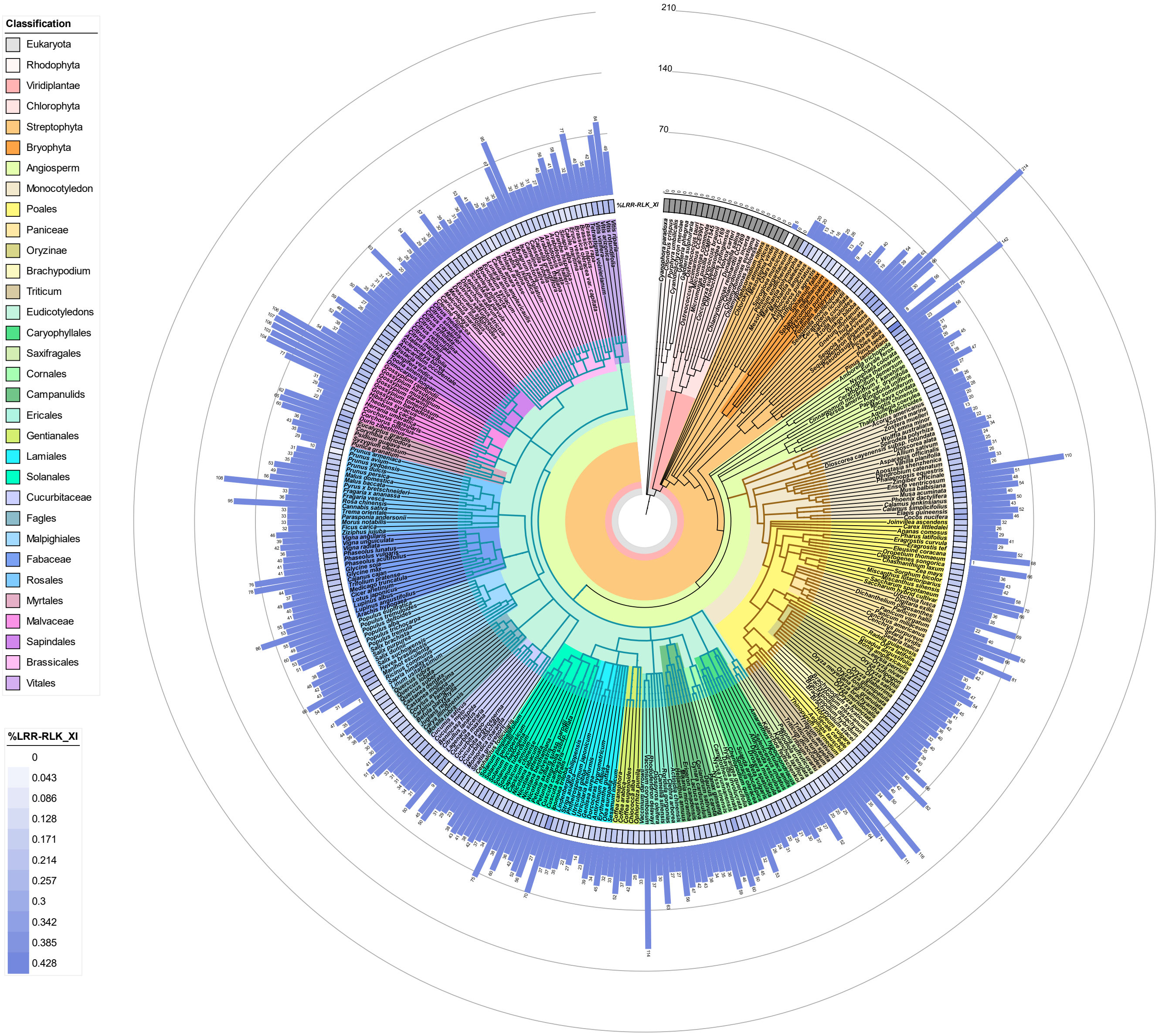
Classification
Eukaryota
Rhodophyta
Viridiplantae
Chlorophyta
Streptophyta
Bryophyta
Angiosperm
Monocotyledon
Poales
Paniceae
Oryzinae
Brachypodium
Triticum
Eudicotyledons
Caryophyllales
Saxifragales
Cornales
Campanulids
Ericales
Gentianales
Lamiales
Solanales
Cucurbitaceae
Fagles
Malpighiales
Fabaceae
Rosales
Myrtales
Malvaceae
Sapindales
Brassicales
Vitales
%LRR-RLK_XI
0
0.043
0.086
0.128
0.171
0.214
0.257
0.3
0.342
0.385
0.428
210
140
70
%LRR-RLK_XI

p

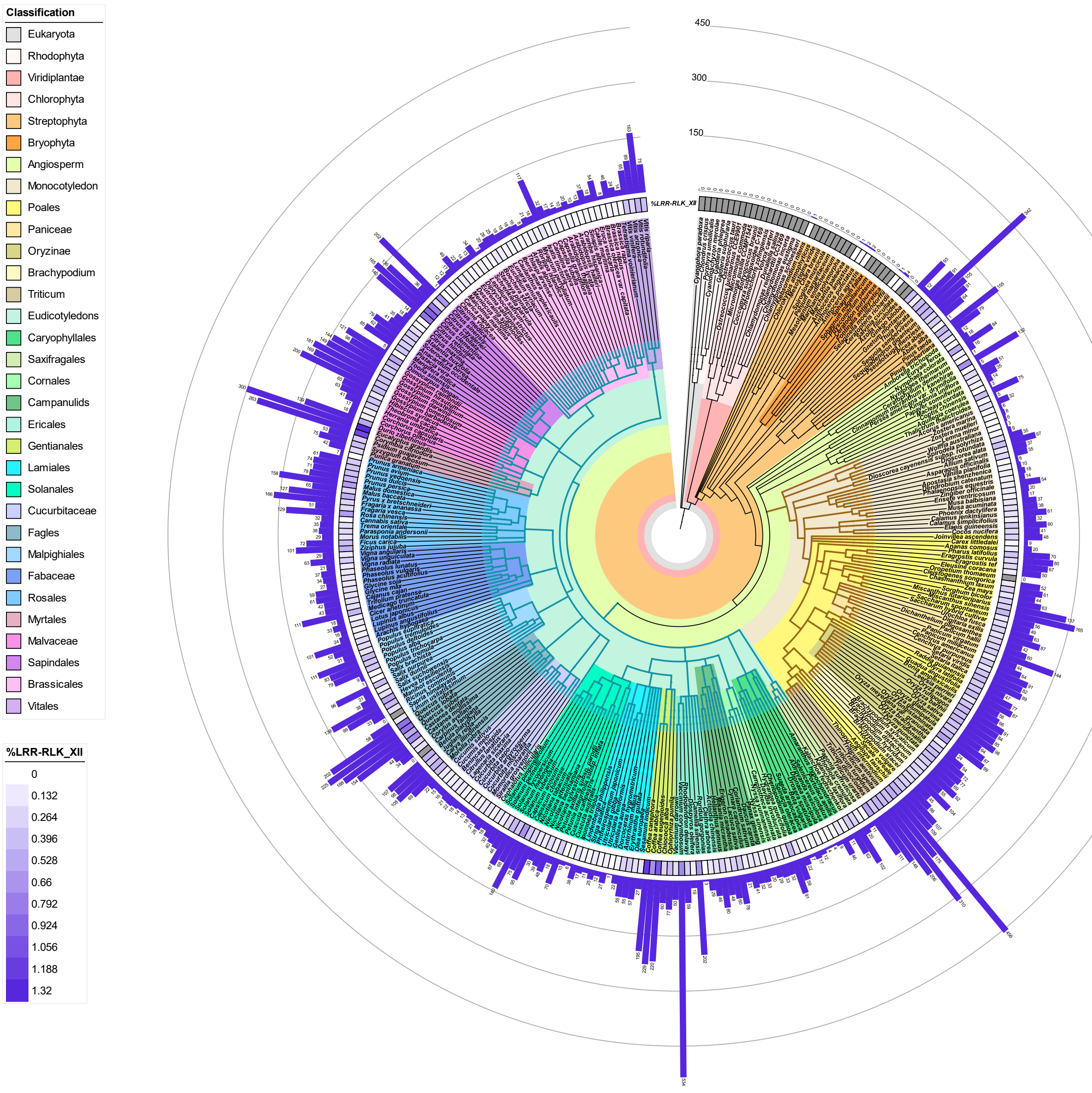

Classification
Eukaryota
Rhodophyta
Viridiplantae
Chlorophyta
Streptophyta
Bryophyta
Angiosperm
Monocotyledon
Poales
Paniceae
Oryzinae
Brachypodium
Triticum
Eudicotyledons
Caryophyllales
Saxifragales
Cornales
Campanulids
Ericales
Gentianales
Lamiales
Solanales
Cucurbitaceae
Fagles
Malpighiales
Fabaceae
Rosales
Myrtales
Malvaceae
Sapindales
Brassicales
Vitales
%LRR-RLK_XII
0
0.132
0.264
0.396
0.528
0.66
0.792
0.924
1.056
1.188
1.32
450
300
150
%LRR-RLK_XII

q

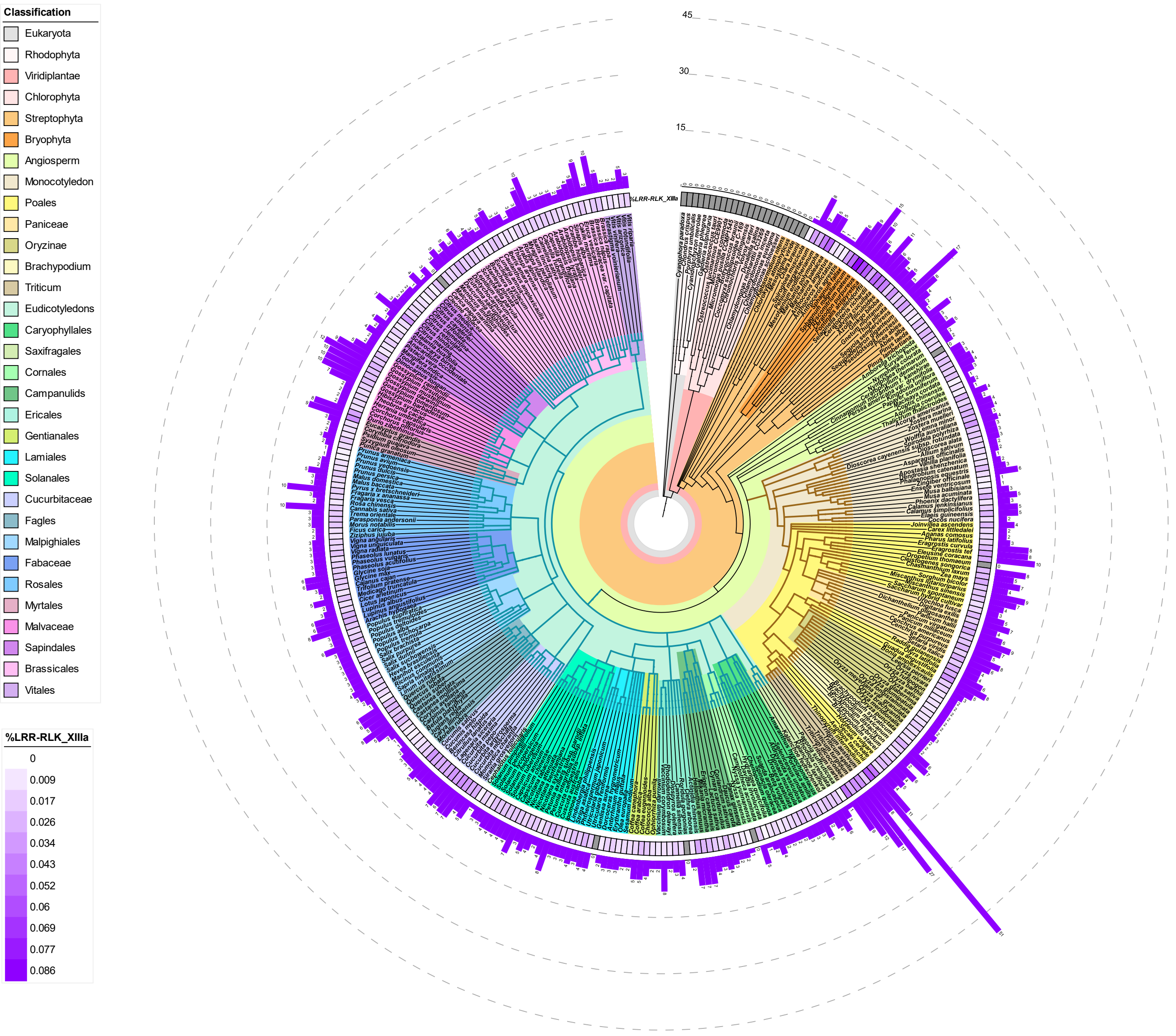
Classification
Eukaryota
Rhodophyta
Viridiplantae
Chlorophyta
Streptophyta
Bryophyta
Angiosperm
Monocotyledon
Poales
Paniceae
Oryzinae
Brachypodium
Triticum
Eudicotyledons
Caryophyllales
Saxifragales
Cornales
Campanulids
Ericales
Gentianales
Lamiales
Solanales
Cucurbitaceae
Fagles
Malpighiales
Fabaceae
Rosales
Myrtales
Malvaceae
Sapindales
Brassicales
Vitales
%LRR-RLK_XIIIa
0
0.009
0.017
0.026
0.034
0.043
0.052
0.06
0.069
0.077
0.086
45
30
15

r

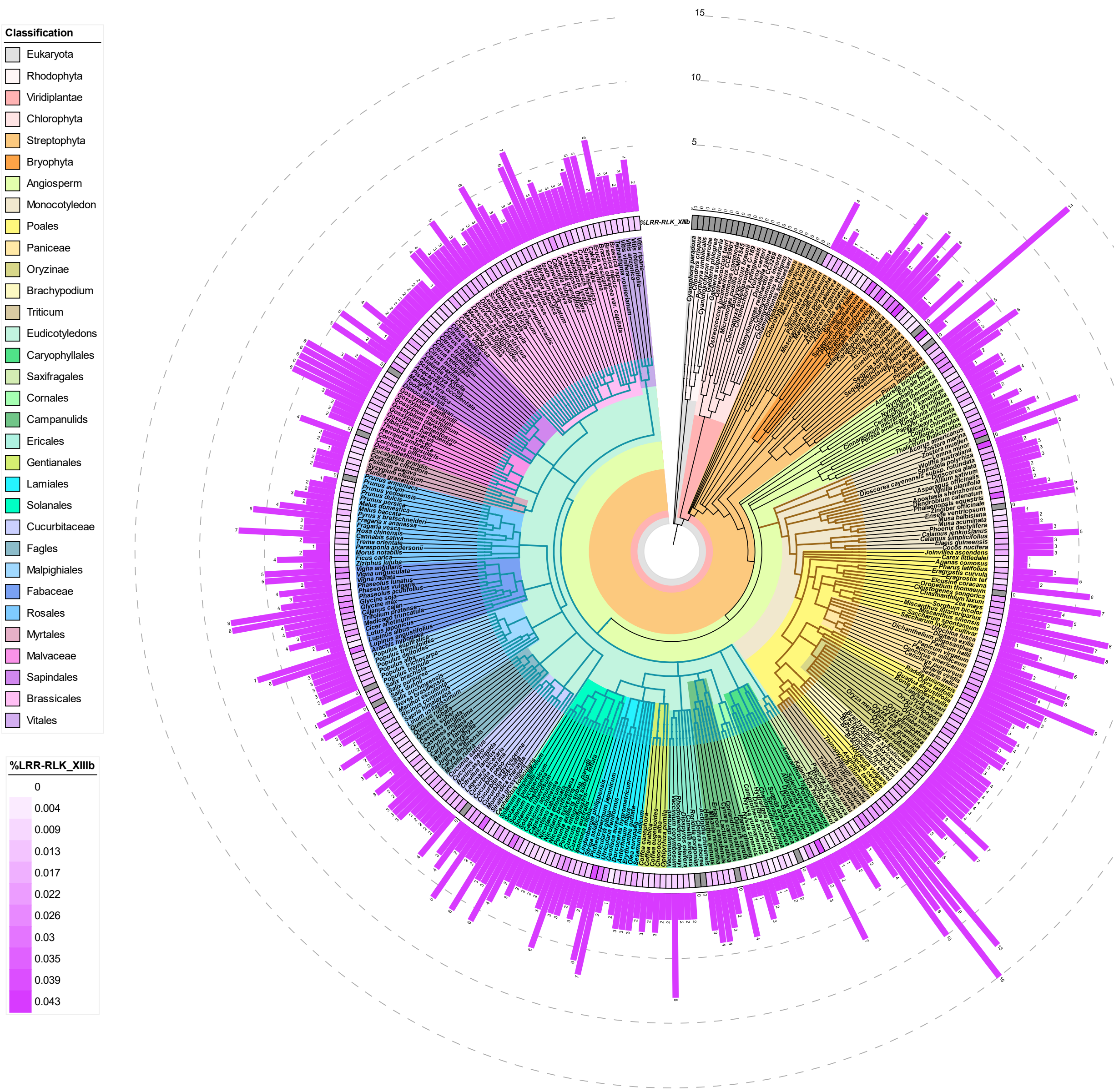
Classification
Eukaryota
Rhodophyta
Viridiplantae
Chlorophyta
Streptophyta
Bryophyta
Angiosperm
Monocotyledon
Poales
Paniceae
Oryzinae
Brachypodium
Triticum
Eudicotyledons
Caryophyllales
Saxifragales
Cornales
Campanulids
Ericales
Gentianales
Lamiales
Solanales
Cucurbitaceae
Fagles
Malpighiales
Fabaceae
Rosales
Myrtales
Malvaceae
Sapindales
Brassicales
Vitales
%LRR-RLK_XIIIb
0
0.004
0.009
0.013
0.017
0.022
0.026
0.03
0.035
0.039
0.043
15
10
5
%LRR-RLK_XIIIb

s

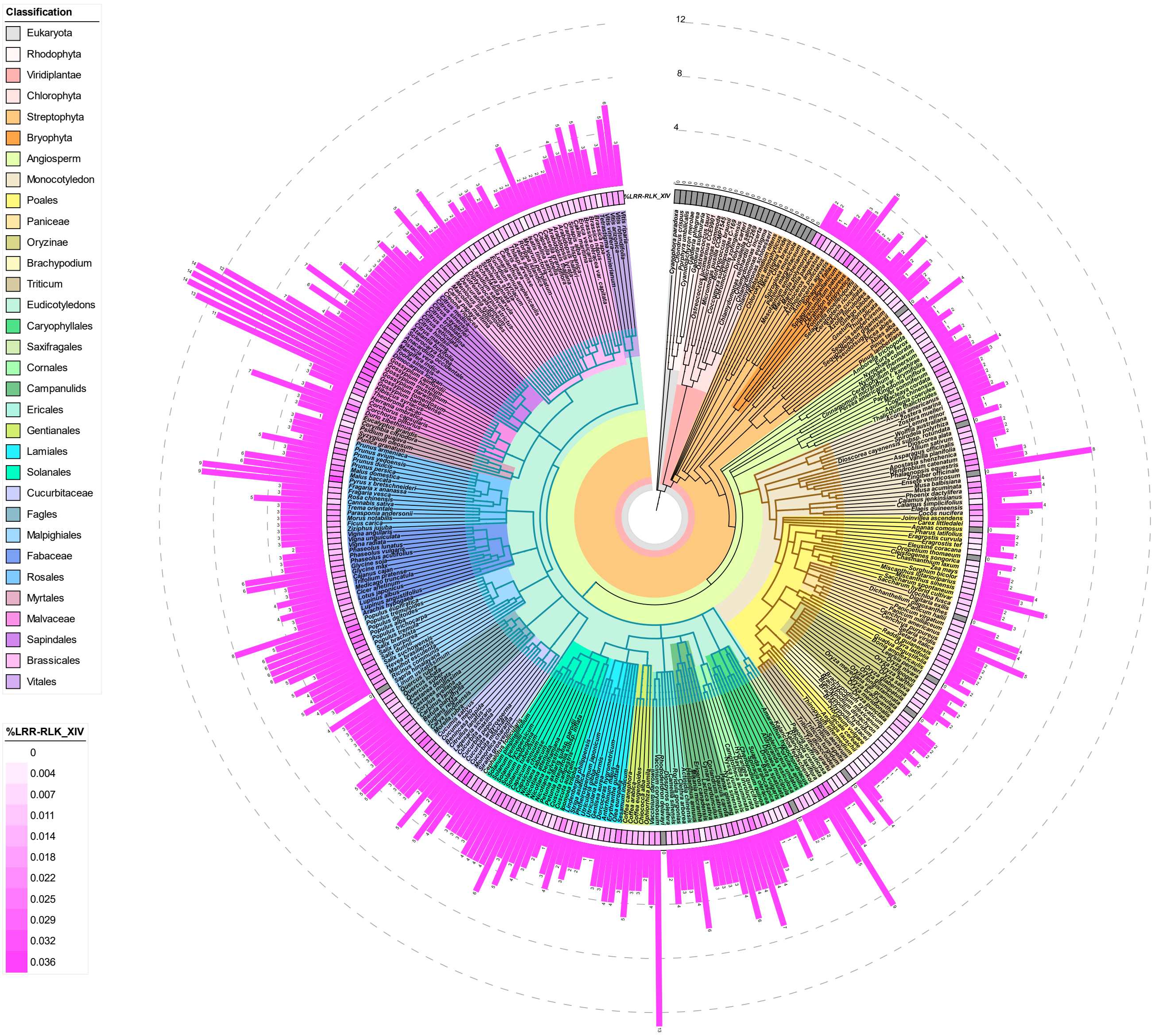
Classification
Eukaryota
Rhodophyta
Viridiplantae
Chlorophyta
Streptophyta
Bryophyta
Angiosperm
Monocotyledon
Poales
Paniceae
Oryzinae
Brachypodium
Triticum
Eudicotyledons
Caryophyllales
Saxifragales
Cornales
Campanulids
Ericales
Gentianales
Lamiales
Solanales
Cucurbitaceae
Fagles
Malpighiales
Fabaceae
Rosales
Myrtales
Malvaceae
Sapindales
Brassicales
Vitales
%LRR-RLK_XIV
0
0.004
0.007
0.011
0.014
0.018
0.022
0.025
0.029
0.032
0.036
12
8
4
%LRR-RLK_XIV

t

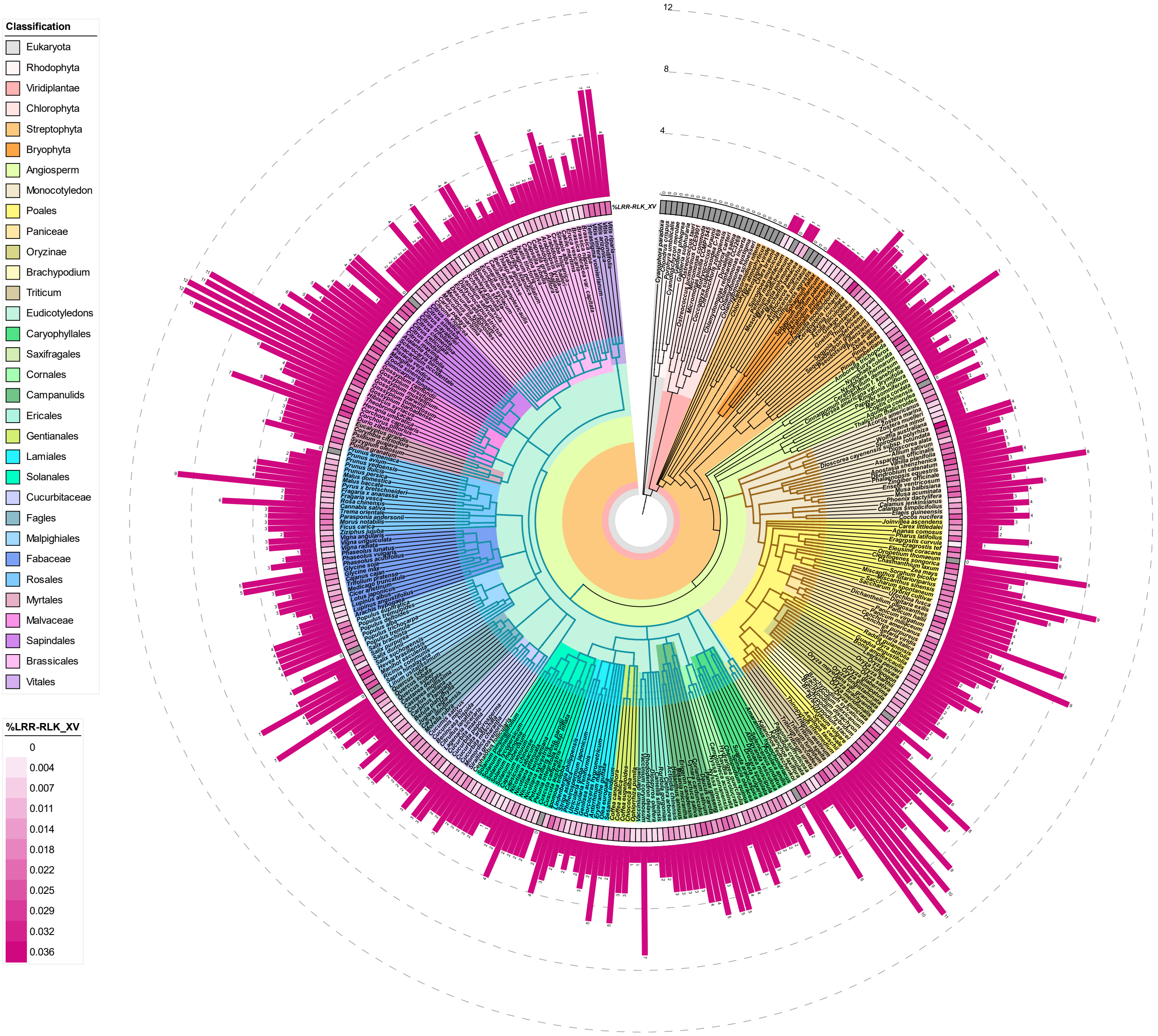
Classification
Eukaryota
Rhodophyta
Viridiplantae
Chlorophyta
Streptophyta
Bryophyta
Angiosperm
Monocotyledon
Poales
Paniceae
Oryzinae
Brachypodium
Triticum
Eudicotyledons
Caryophyllales
Saxifragales
Cornales
Campanulids
Ericales
Gentianales
Lamiales
Solanales
Cucurbitaceae
Fagles
Malpighiales
Fabaceae
Rosales
Myrtales
Malvaceae
Sapindales
Brassicales
Vitales
%LRR-RLK_XV
0
0.004
0.007
0.011
0.014
0.018
0.022
0.025
0.029
0.032
0.036
12
8
4
%LRR-RLK_XV

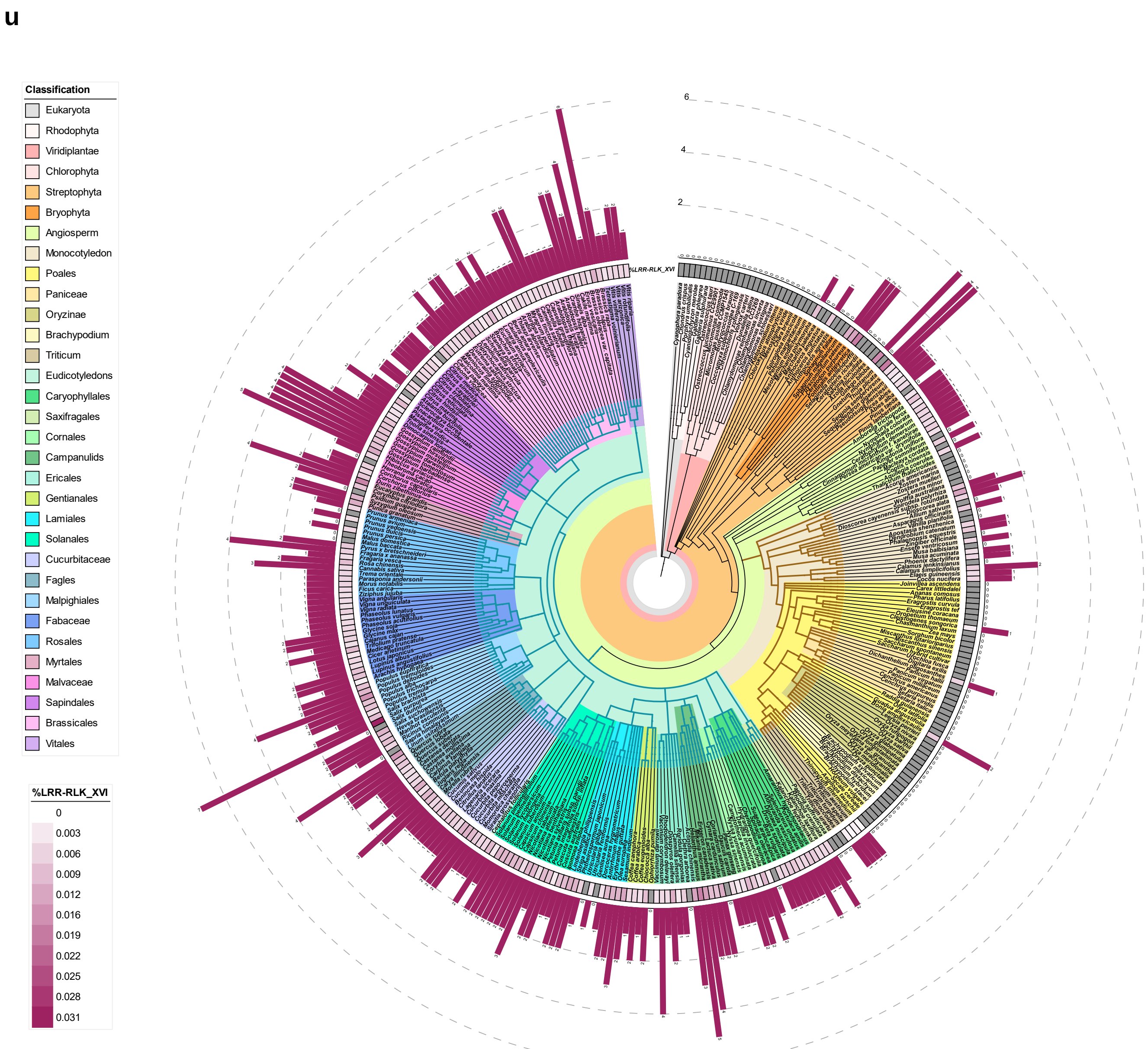
u
Classification
Eukaryota
Rhodophyta
Viridiplantae
Chlorophyta
Streptophyta
Bryophyta
Angiosperm
Monocotyledon
Poales
Paniceae
Oryzinae
Brachypodium
Triticum
Eudicotyledons
Caryophyllales
Saxifragales
Cornales
Campanulids
Ericales
Gentianales
Lamiales
Solanales
Cucurbitaceae
Fagles
Malpighiales
Fabaceae
Rosales
Myrtales
Malvaceae
Sapindales
Brassicales
Vitales
%LRR-RLK_XVI
0
0.003
0.006
0.009
0.012
0.016
0.019
0.022
0.025
0.028
0.031
6
4
2
%LRR-RLK_XVI

v

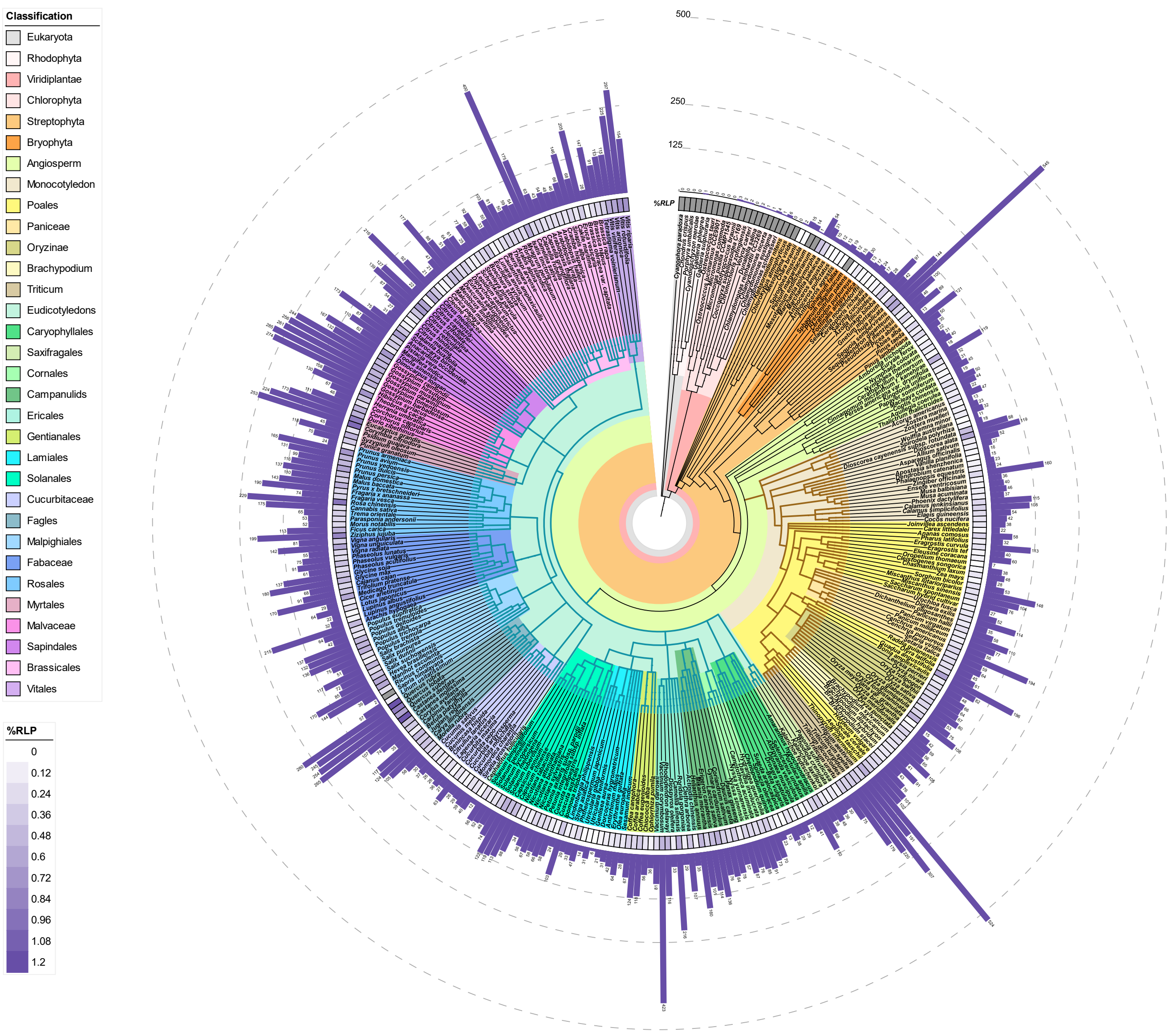
Classification
Eukaryota
Rhodophyta
Viridiplantae
Chlorophyta
Streptophyta
Bryophyta
Angiosperm
Monocotyledon
Poales
Paniceae
Oryzinae
Brachypodium
Triticum
Eudicotyledons
Caryophyllales
Saxifragales
Cornales
Campanulids
Ericales
Gentianales
Lamiales
Solanales
Cucurbitaceae
Fagles
Malpighiales
Fabaceae
Rosales
Myrtales
Malvaceae
Sapindales
Brassicales
Vitales
%RLP
0
0.12
0.24
0.36
0.48
0.6
0.72
0.84
0.96
1.08
1.2
500
250
125
%RLP

w

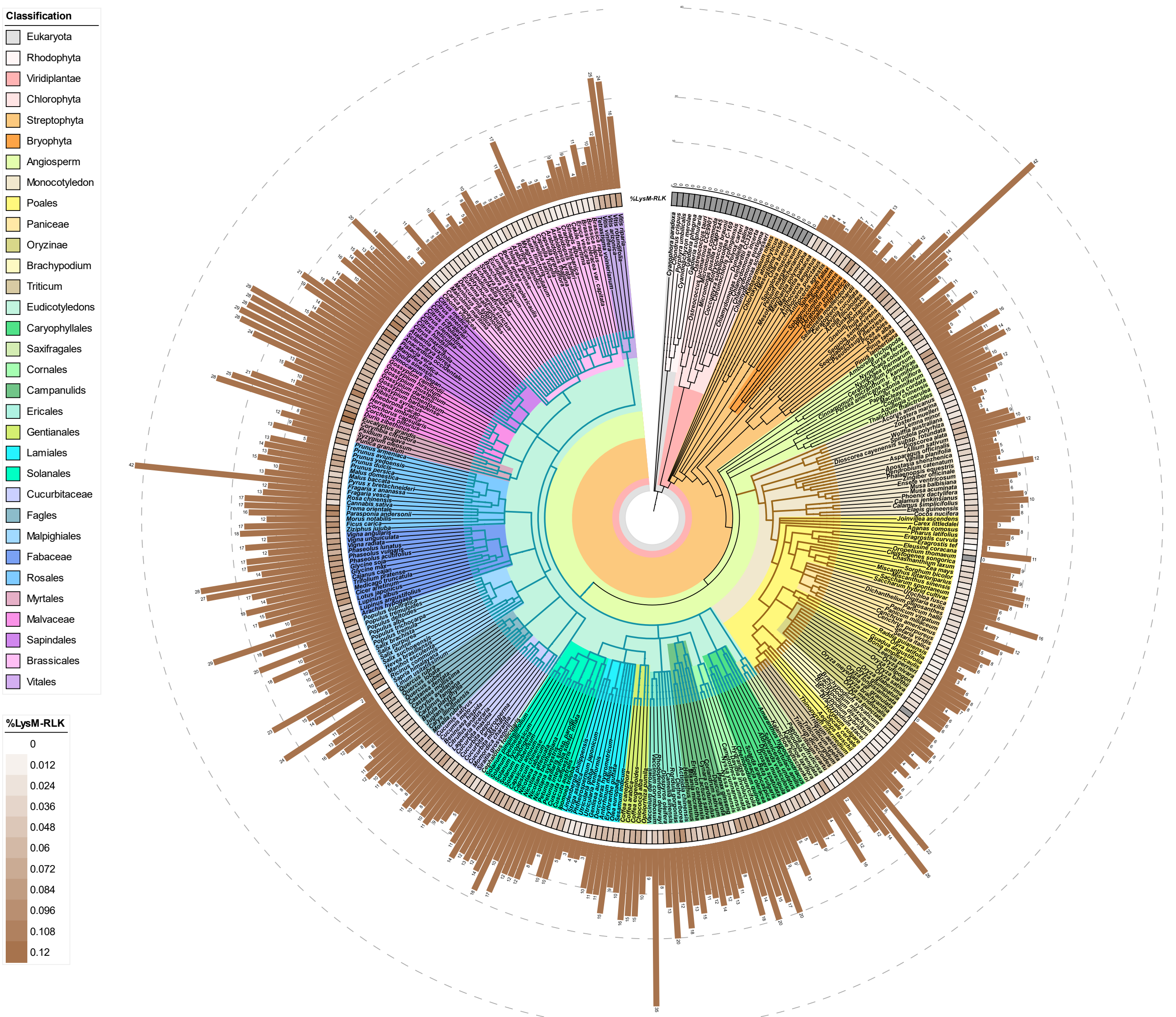
Classification
Eukaryota
Rhodophyta
Viridiplantae
Chlorophyta
Streptophyta
Bryophyta
Angiosperm
Monocotyledon
Poales
Paniceae
Oryzinae
Brachypodium
Triticum
Eudicotyledons
Caryophyllales
Saxifragales
Cornales
Campanulids
Ericales
Gentianales
Lamiales
Solanales
Cucurbitaceae
Fagles
Malpighiales
Fabaceae
Rosales
Myrtales
Malvaceae
Sapindales
Brassicales
Vitales
%LysM-RLK
0
0.012
0.024
0.036
0.048
0.06
0.072
0.084
0.096
0.108
0.12
%LysM-RLK

Supplementary figure 1

x

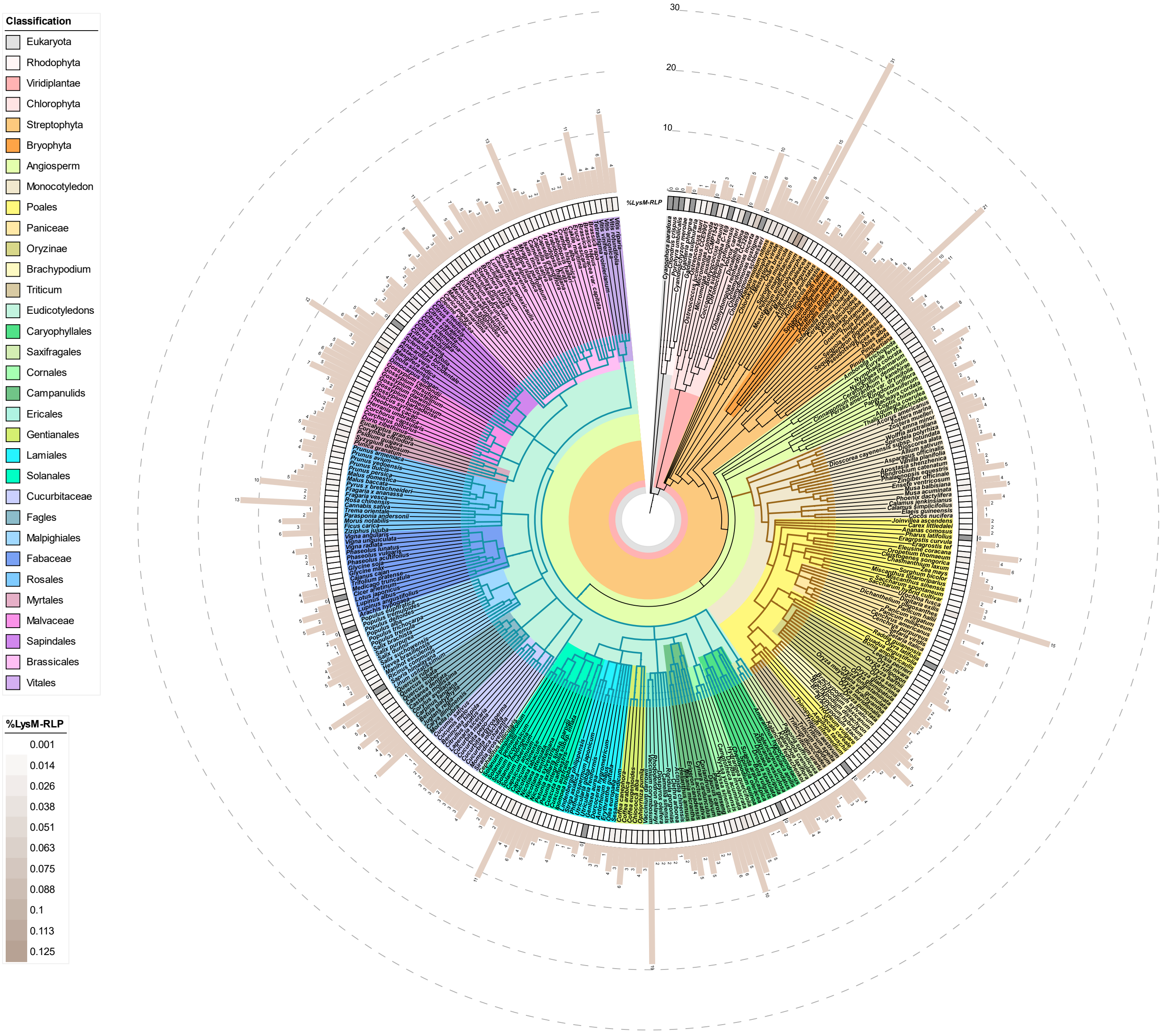

y

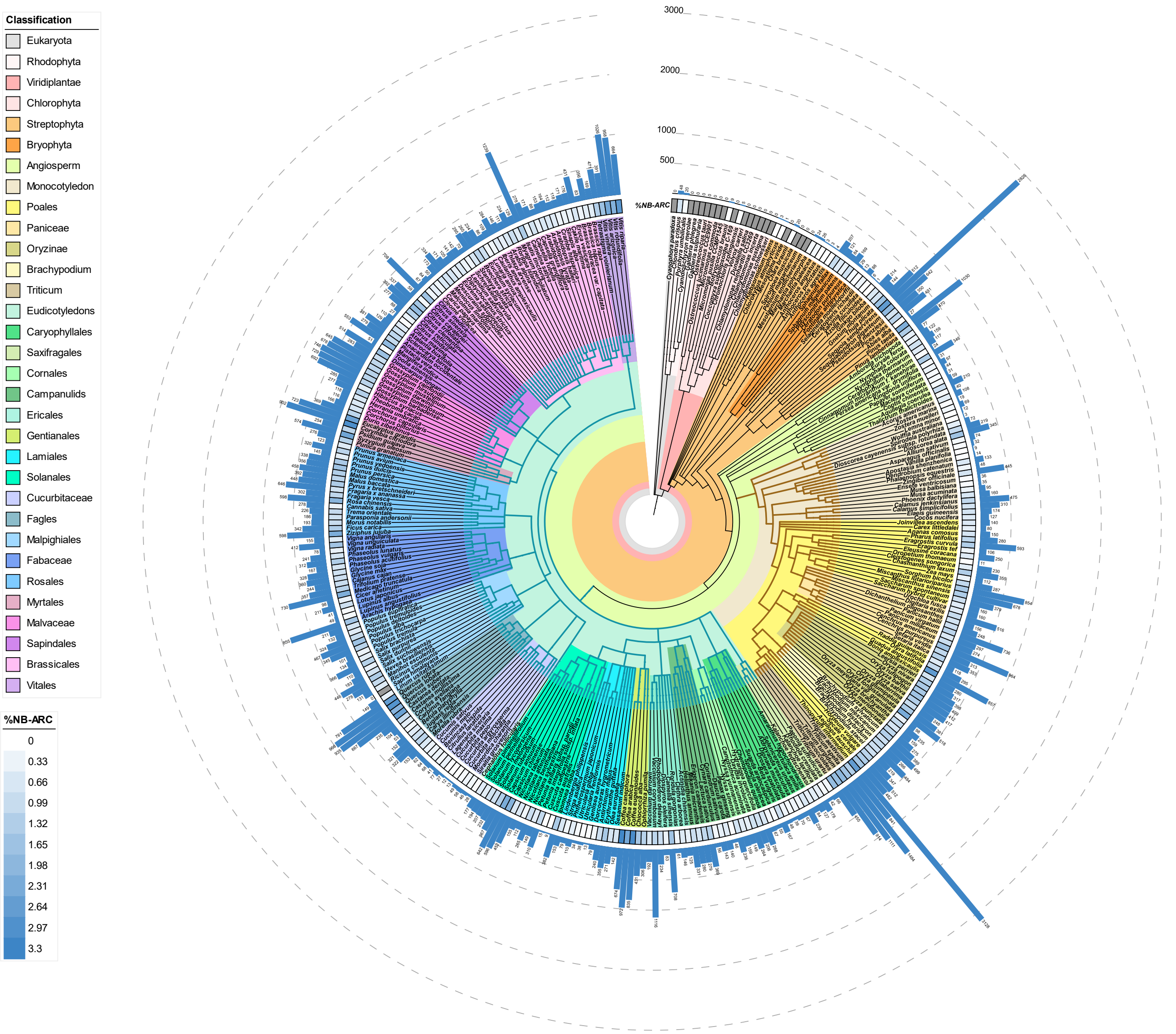

Supplementary figure 1. Number of genes within each receptor family from 350 genomes. Bar charts represent the number (count) of genes from a, LRR-RLK (total); b, LRR-RLK-I; c, LRR-RLK-II; d, LRR-RLK-III; e, LRR-RLK-IV; f, LRR-RLK-V; g, LRR-RLK-VI_1; h, LRR-RLK-VI_2; i, LRR-RLK-VII; j, LRR-RLK-VIII_1; k, LRR-RLK-VIII_2; l, LRR-RLK-IX; m, LRR-RLK-Xa; n, LRR-RLK-Xb; o, LRR-RLK-XI; p, LRR-RLK-XII; q, LRR-RLK-XIIIa; r, LRR-RLK-XIIIb; s, LRR-RLK-XIV; t, LRR-RLK-XV; u, LRR-RLK-XVI; v, LRR-RLP; w, LysM-RLK; x, LysM-RLP; y, NB-ARC in each species. Heatmaps represent the percentage (%) of genes from each receptor family in the proteome of each species.

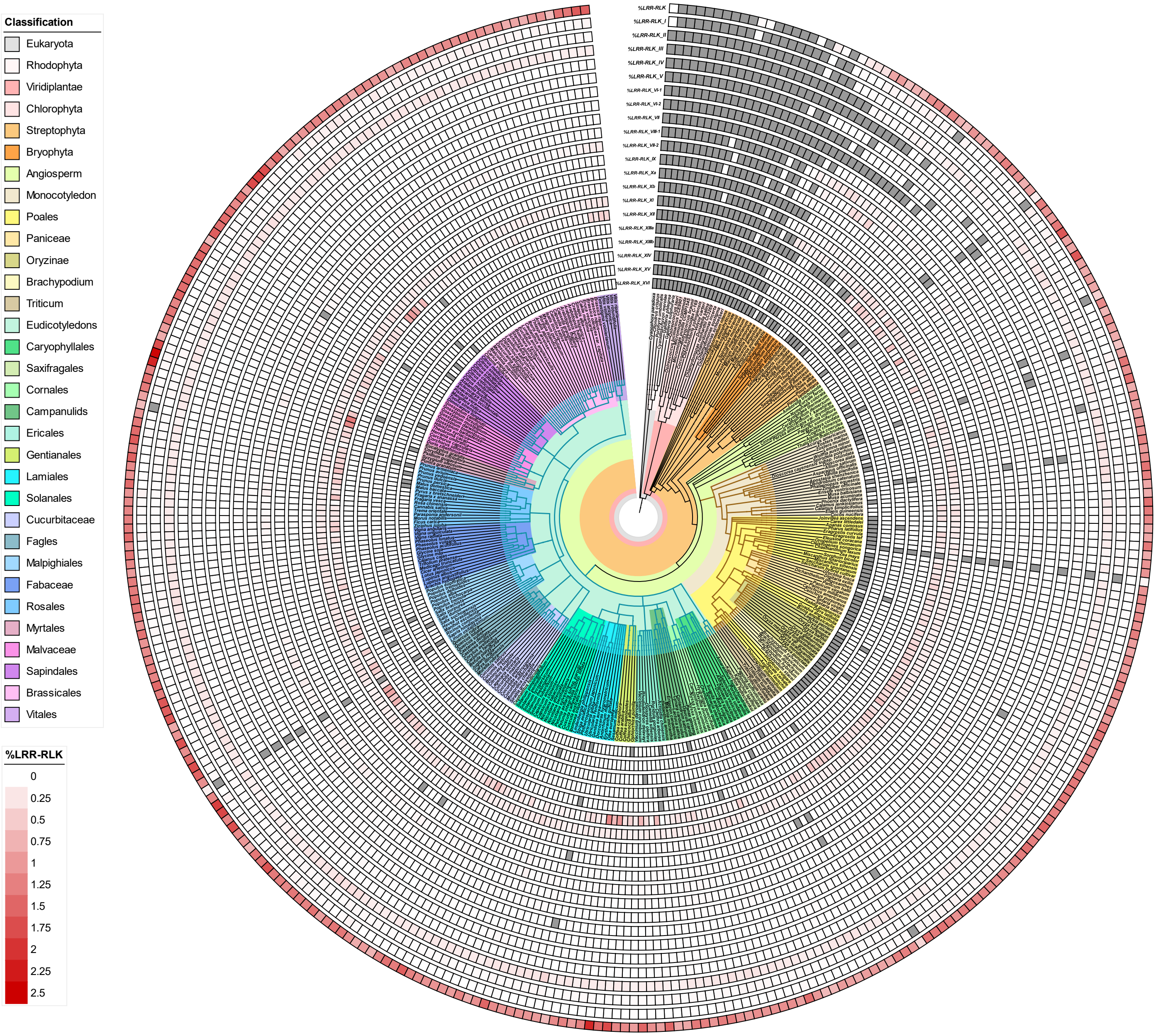

Supplementary figure 2. Size of the LRR-RLK subgroup gene families in 350 genomes. Heatmaps represent the percentage (%) of genes from LRR-RLK subgroups (I-XVI and total) in the proteome of each species. The maximum values of the subgroup heatmaps are adjusted to 2.5%.

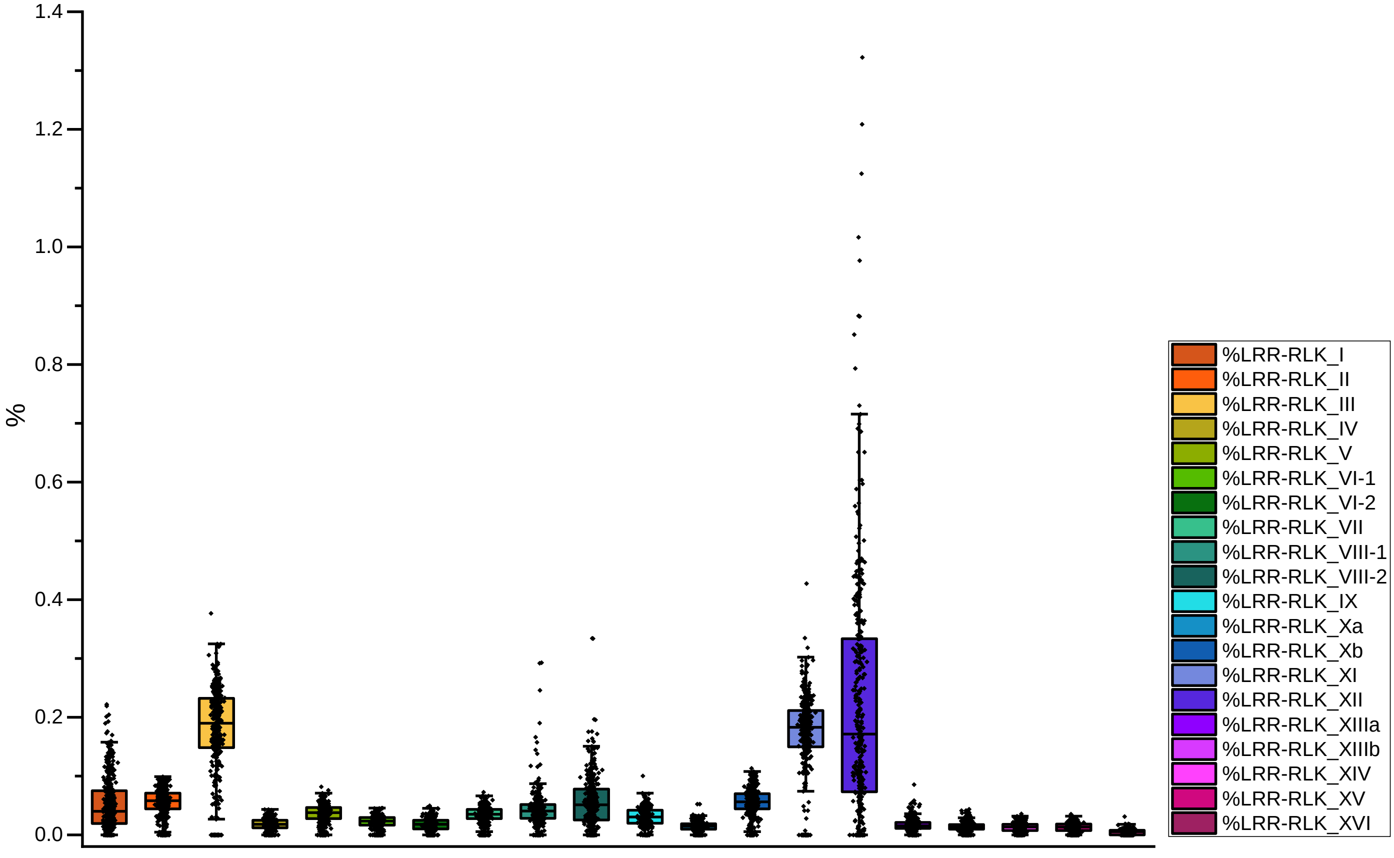

Supplementary figure 3. Percentage of the LRR-RLK subgroup gene families in 350 genomes. Boxplots represent the percentage (%) of genes from LRR-RLK subgroups (I-XVI) in the genome from 350 species (n=350 species for each LRR-RLK subgroup). Box-plot elements: centre line, median; bounds of box, 25th and 75th percentiles; whiskers, 1.5 × IQR from 25th and 75th percentiles.

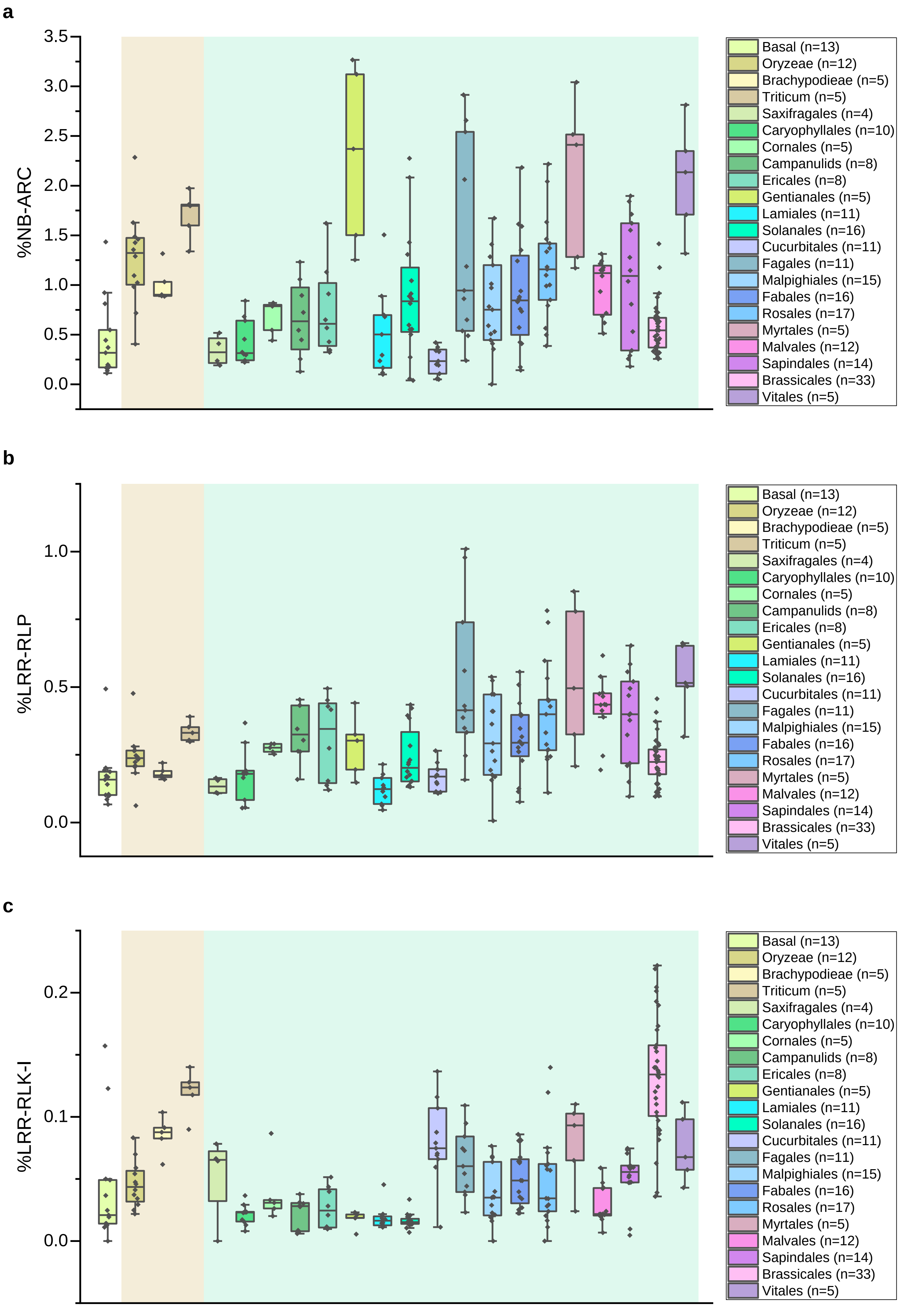
a
%NB-ARC
3.5
3.0
2.5
2.0
1.5
1.0
0.5
0.0
Basal (n=13)
Oryzeae (n=12)
Brachypodieae (n=5)
Triticum (n=5)
Saxifragales (n=4)
Caryophyllales (n=10)
Cornales (n=5)
Campanulids (n=8)
Ericales (n=8)
Gentianales (n=5)
Lamiales (n=11)
Solanales (n=16)
Cucurbitales (n=11)
Fagales (n=11)
Malpighiales (n=15)
Fabales (n=16)
Rosales (n=17)
Myrtales (n=5)
Malvales (n=12)
Sapindales (n=14)
Brassicales (n=33)
Vitales (n=5)
b
%LRR-RLP
1.0
0.5
0.0
c
%LRR-RLK-I
0.2
0.1
0.0

d

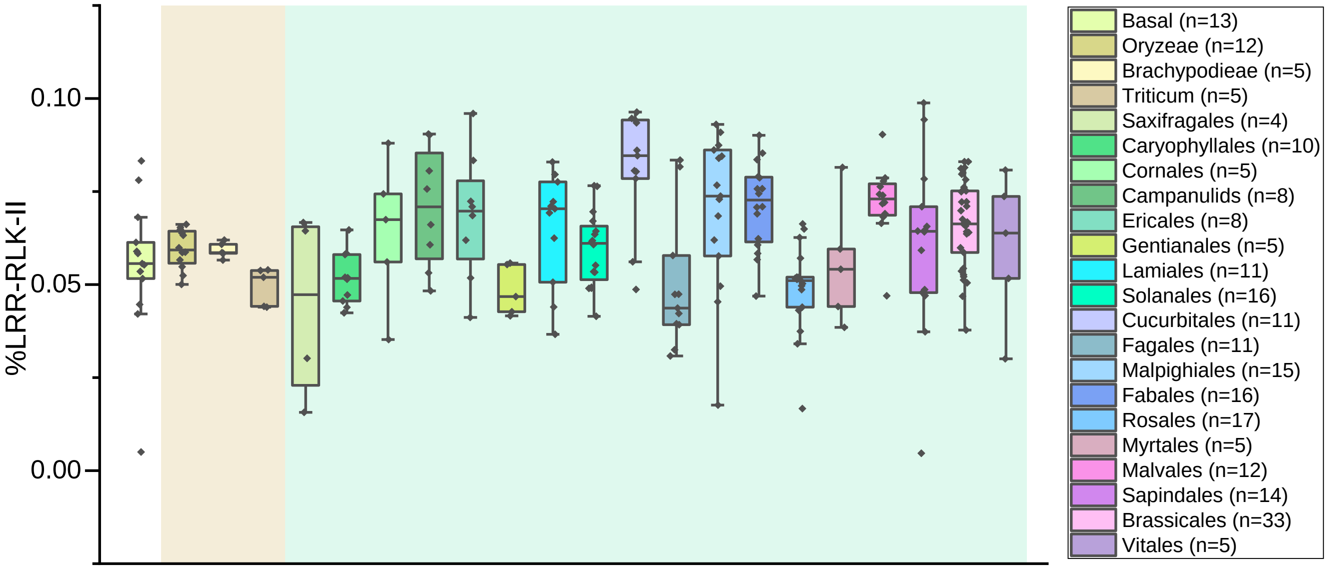
%LRR-RLK-II
0.10
0.05
0.00
Basal (n=13)
Oryzeae (n=12)
Brachypodieae (n=5)
Triticum (n=5)
Saxifragales (n=4)
Caryophyllales (n=10)
Cornales (n=5)
Campanulids (n=8)
Ericales (n=8)
Gentianales (n=5)
Lamiales (n=11)
Solanales (n=16)
Cucurbitales (n=11)
Fagales (n=11)
Malpighiales (n=15)
Fabales (n=16)
Rosales (n=17)
Myrtales (n=5)
Malvales (n=12)
Sapindales (n=14)
Brassicales (n=33)
Vitales (n=5)

e

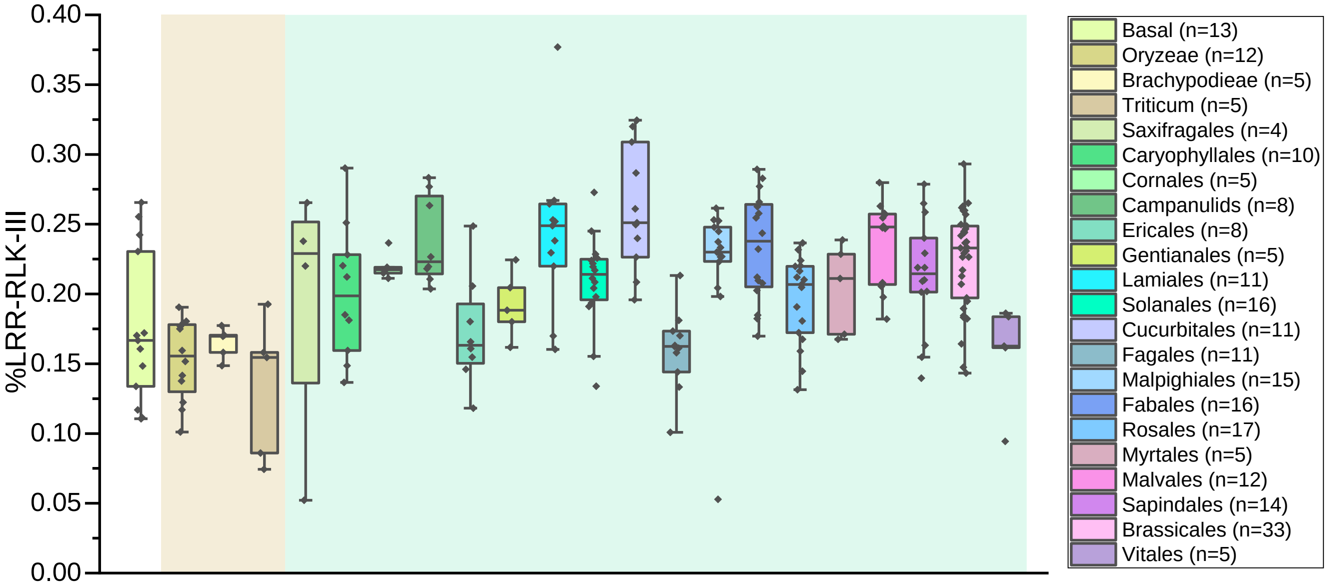
%LRR-RLK-III
0.40
0.35
0.30
0.25
0.20
0.15
0.10
0.05
0.00
Basal (n=13)
Oryzeae (n=12)
Brachypodieae (n=5)
Triticum (n=5)
Saxifragales (n=4)
Caryophyllales (n=10)
Cornales (n=5)
Campanulids (n=8)
Ericales (n=8)
Gentianales (n=5)
Lamiales (n=11)
Solanales (n=16)
Cucurbitales (n=11)
Fagales (n=11)
Malpighiales (n=15)
Fabales (n=16)
Rosales (n=17)
Myrtales (n=5)
Malvales (n=12)
Sapindales (n=14)
Brassicales (n=33)
Vitales (n=5)

f

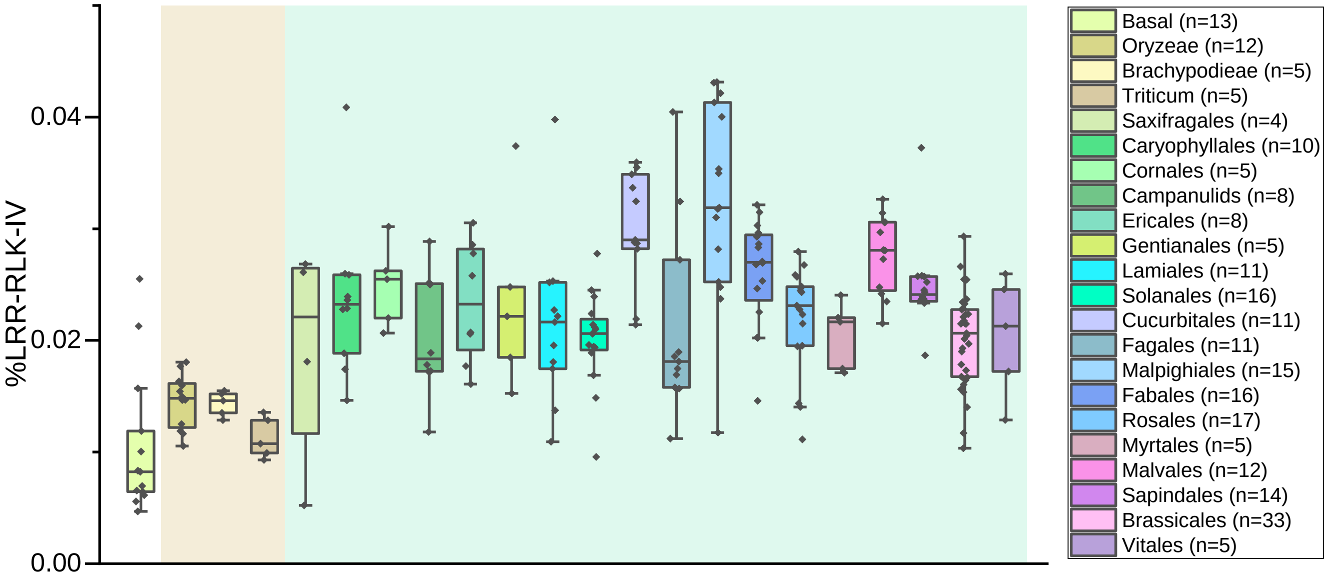
%LRR-RLK-IV
0.04
0.02
0.00
Basal (n=13)
Oryzeae (n=12)
Brachypodieae (n=5)
Triticum (n=5)
Saxifragales (n=4)
Caryophyllales (n=10)
Cornales (n=5)
Campanulids (n=8)
Ericales (n=8)
Gentianales (n=5)
Lamiales (n=11)
Solanales (n=16)
Cucurbitales (n=11)
Fagales (n=11)
Malpighiales (n=15)
Fabales (n=16)
Rosales (n=17)
Myrtales (n=5)
Malvales (n=12)
Sapindales (n=14)
Brassicales (n=33)
Vitales (n=5)

g

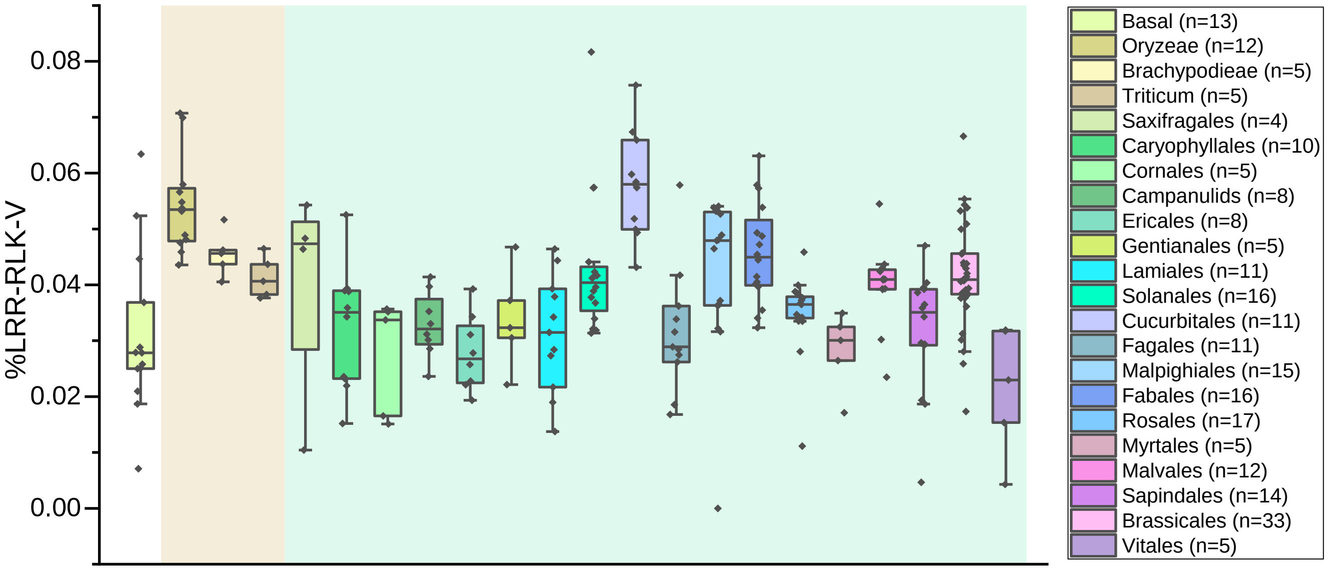
%LRR-RLK-V
0.00
0.02
0.04
0.06
0.08
Basal (n=13)
Oryzeae (n=12)
Brachypodieae (n=5)
Triticum (n=5)
Saxifragales (n=4)
Caryophyllales (n=10)
Cornales (n=5)
Campanulids (n=8)
Ericales (n=8)
Gentianales (n=5)
Lamiales (n=11)
Solanales (n=16)
Cucurbitales (n=11)
Fagales (n=11)
Malpighiales (n=15)
Fabales (n=16)
Rosales (n=17)
Myrtales (n=5)
Malvales (n=12)
Sapindales (n=14)
Brassicales (n=33)
Vitales (n=5)

h

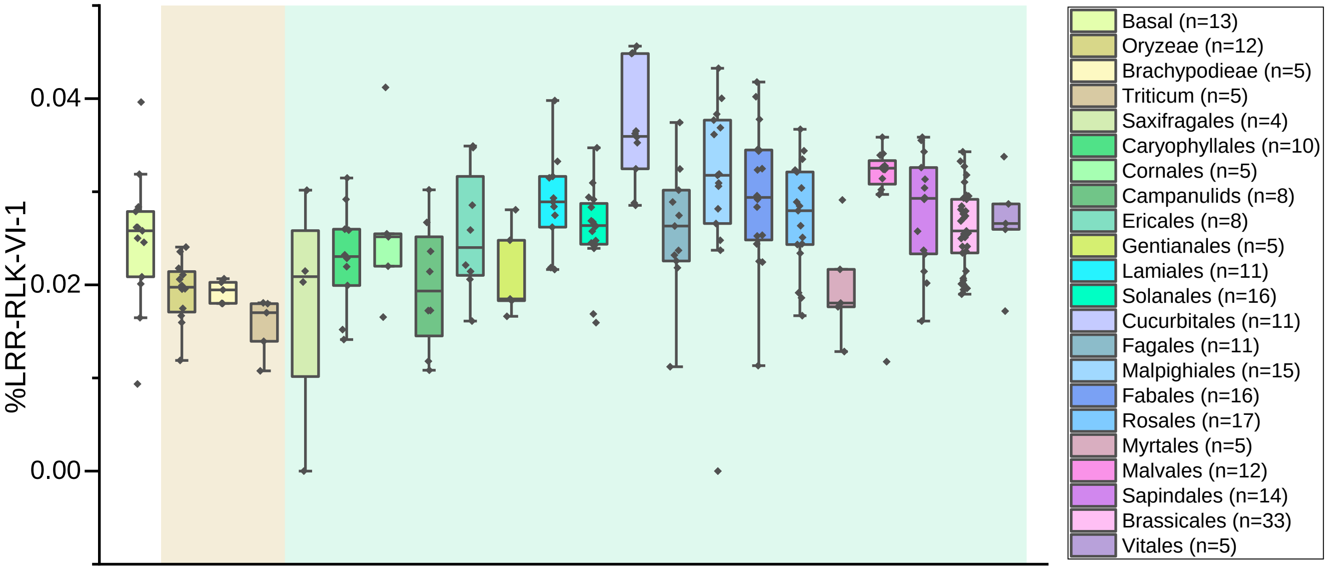
%LRR-RLK-VI-1
0.00
0.02
0.04
Basal (n=13)
Oryzeae (n=12)
Brachypodieae (n=5)
Triticum (n=5)
Saxifragales (n=4)
Caryophyllales (n=10)
Cornales (n=5)
Campanulids (n=8)
Ericales (n=8)
Gentianales (n=5)
Lamiales (n=11)
Solanales (n=16)
Cucurbitales (n=11)
Fagales (n=11)
Malpighiales (n=15)
Fabales (n=16)
Rosales (n=17)
Myrtales (n=5)
Malvales (n=12)
Sapindales (n=14)
Brassicales (n=33)
Vitales (n=5)

i

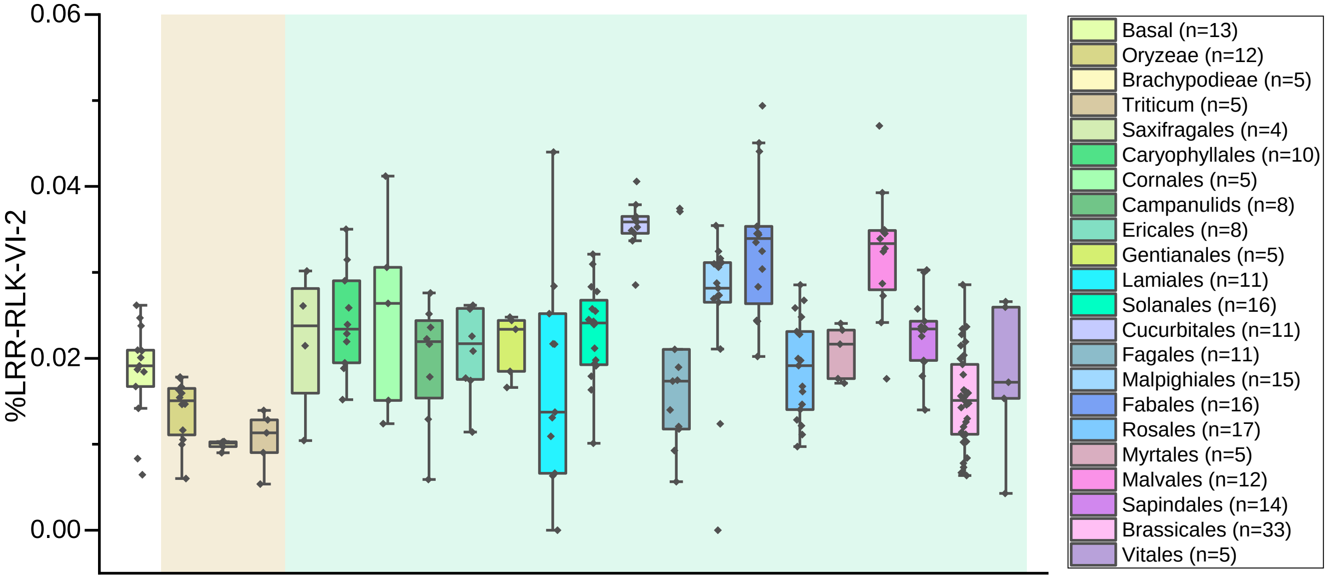
%LRR-RLK-VI-2
0.00
0.02
0.04
0.06
Basal (n=13)
Oryzeae (n=12)
Brachypodieae (n=5)
Triticum (n=5)
Saxifragales (n=4)
Caryophyllales (n=10)
Cornales (n=5)
Campanulids (n=8)
Ericales (n=8)
Gentianales (n=5)
Lamiales (n=11)
Solanales (n=16)
Cucurbitales (n=11)
Fagales (n=11)
Malpighiales (n=15)
Fabales (n=16)
Rosales (n=17)
Myrtales (n=5)
Malvales (n=12)
Sapindales (n=14)
Brassicales (n=33)
Vitales (n=5)

j

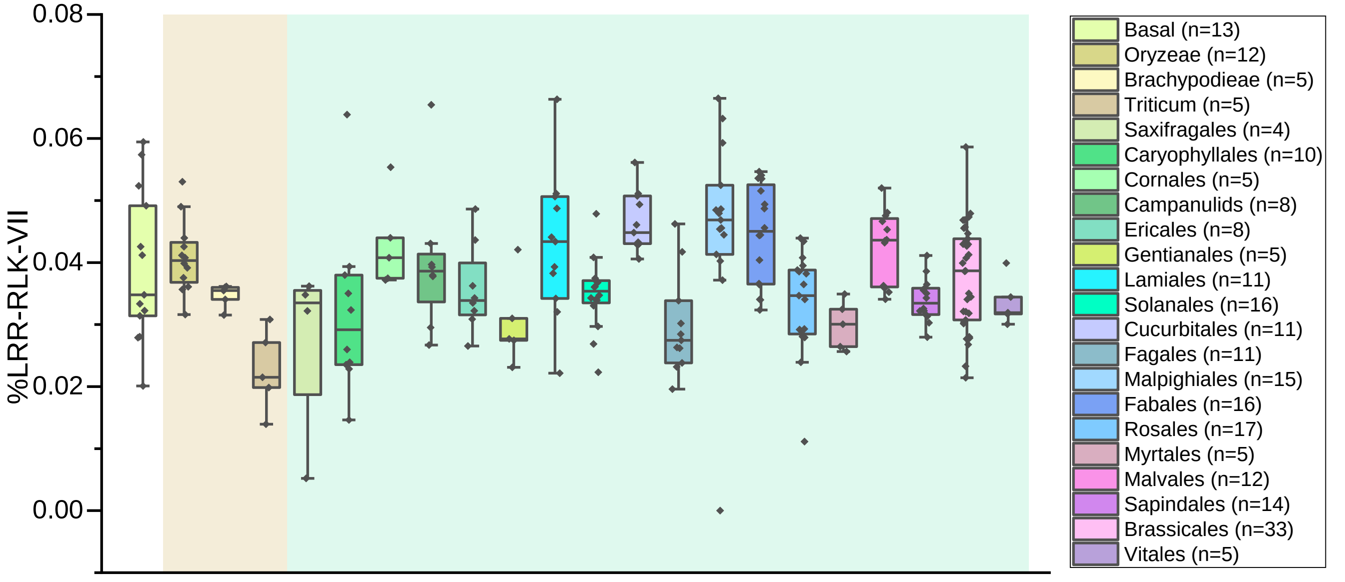
0.08
0.06
0.04
0.02
0.00
%LRR-RLK-VII
Basal (n=13)
Oryzeae (n=12)
Brachypodieae (n=5)
Triticum (n=5)
Saxifragales (n=4)
Caryophyllales (n=10)
Cornales (n=5)
Campanulids (n=8)
Ericales (n=8)
Gentianales (n=5)
Lamiales (n=11)
Solanales (n=16)
Cucurbitales (n=11)
Fagales (n=11)
Malpighiales (n=15)
Fabales (n=16)
Rosales (n=17)
Myrtales (n=5)
Malvales (n=12)
Sapindales (n=14)
Brassicales (n=33)
Vitales (n=5)

k

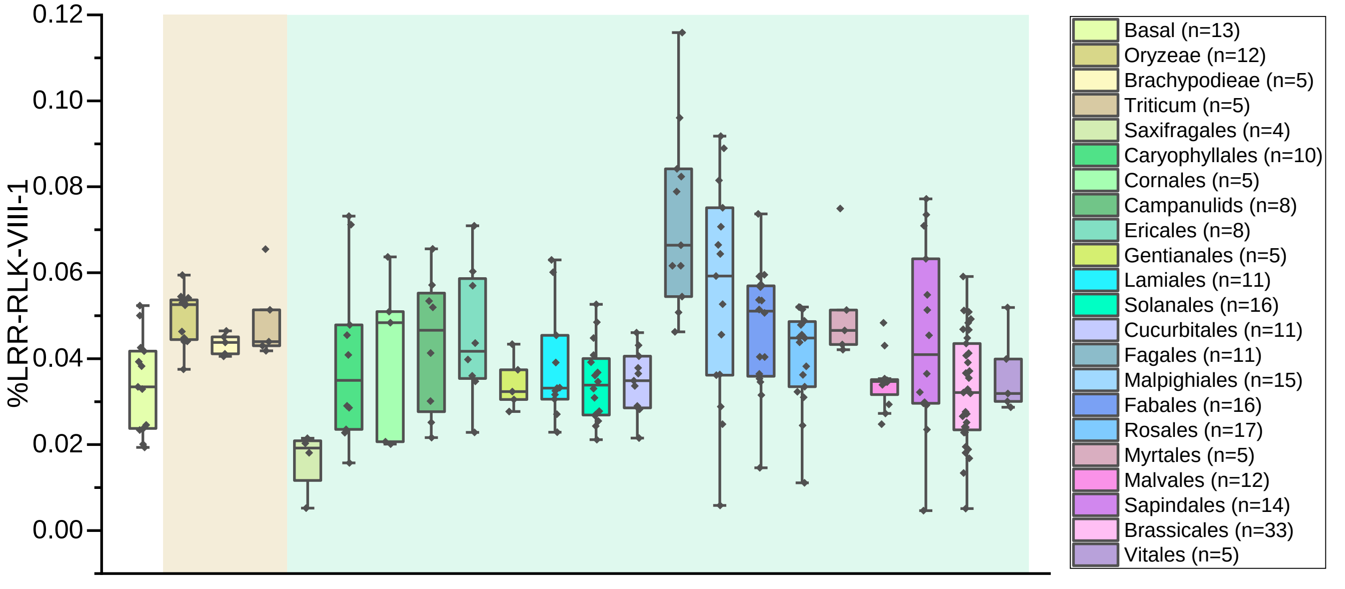
0.12
0.10
0.08
0.06
0.04
0.02
0.00
%LRR-RLK-VIII-1
Basal (n=13)
Oryzeae (n=12)
Brachypodieae (n=5)
Triticum (n=5)
Saxifragales (n=4)
Caryophyllales (n=10)
Cornales (n=5)
Campanulids (n=8)
Ericales (n=8)
Gentianales (n=5)
Lamiales (n=11)
Solanales (n=16)
Cucurbitales (n=11)
Fagales (n=11)
Malpighiales (n=15)
Fabales (n=16)
Rosales (n=17)
Myrtales (n=5)
Malvales (n=12)
Sapindales (n=14)
Brassicales (n=33)
Vitales (n=5)

l

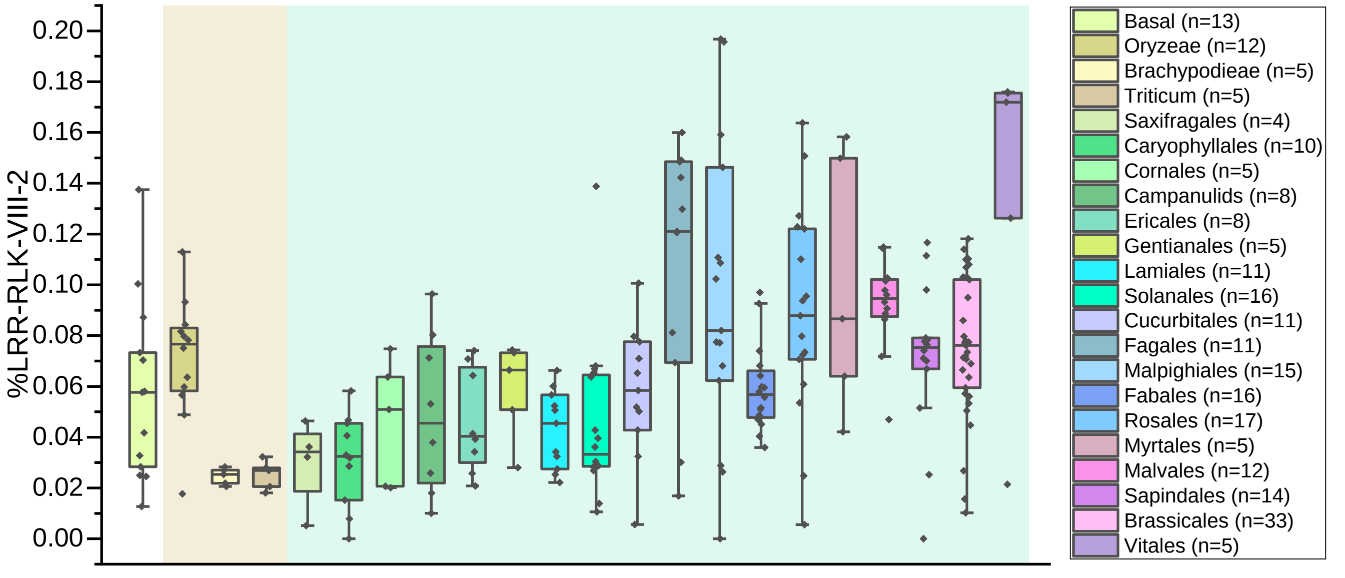
0.20
0.18
0.16
0.14
0.12
0.10
0.08
0.06
0.04
0.02
0.00
%LRR-RLK-VIII-2
Basal (n=13)
Oryzeae (n=12)
Brachypodieae (n=5)
Triticum (n=5)
Saxifragales (n=4)
Caryophyllales (n=10)
Cornales (n=5)
Campanulids (n=8)
Ericales (n=8)
Gentianales (n=5)
Lamiales (n=11)
Solanales (n=16)
Cucurbitales (n=11)
Fagales (n=11)
Malpighiales (n=15)
Fabales (n=16)
Rosales (n=17)
Myrtales (n=5)
Malvales (n=12)
Sapindales (n=14)
Brassicales (n=33)
Vitales (n=5)

m

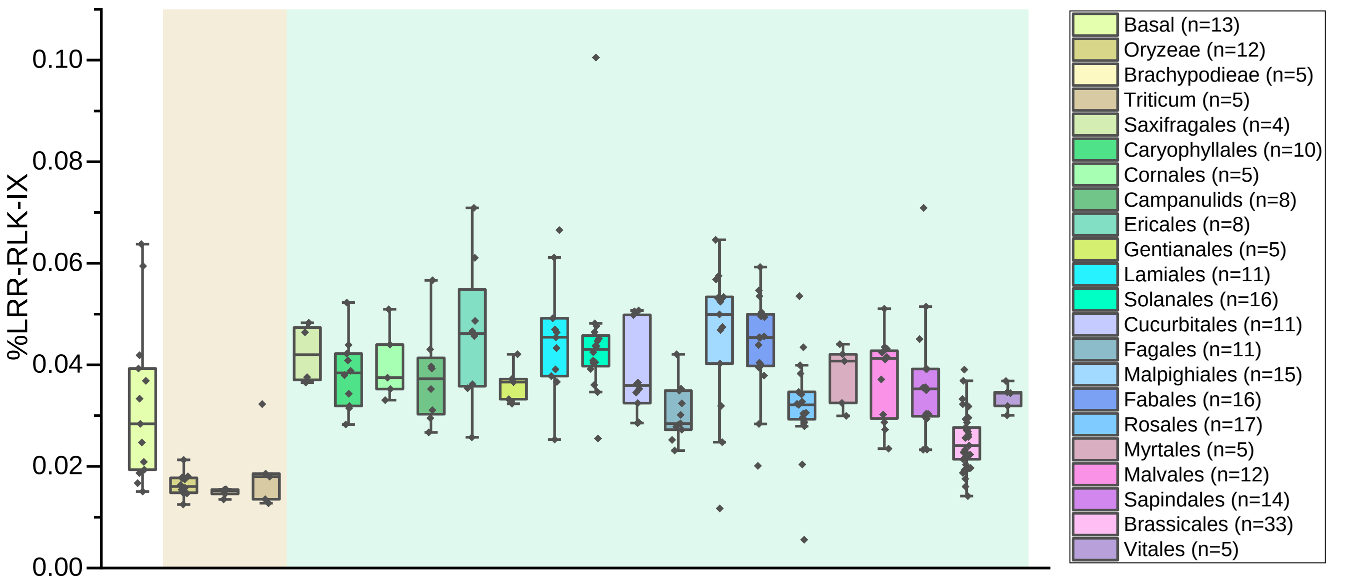
%LRR-RLK-IX
0.00
0.02
0.04
0.06
0.08
0.10
Basal (n=13)
Oryzeae (n=12)
Brachypodieae (n=5)
Triticum (n=5)
Saxifragales (n=4)
Caryophyllales (n=10)
Cornales (n=5)
Campanulids (n=8)
Ericales (n=8)
Gentianales (n=5)
Lamiales (n=11)
Solanales (n=16)
Cucurbitales (n=11)
Fagales (n=11)
Malpighiales (n=15)
Fabales (n=16)
Rosales (n=17)
Myrtales (n=5)
Malvales (n=12)
Sapindales (n=14)
Brassicales (n=33)
Vitales (n=5)

n

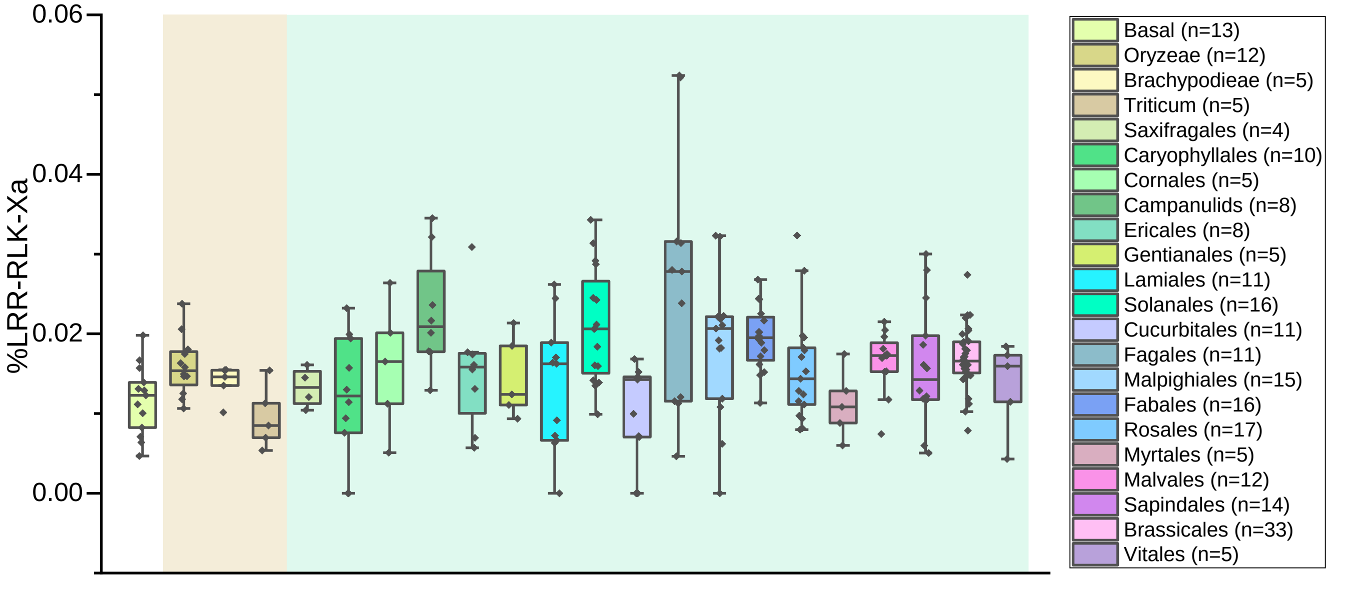
%LRR-RLK-Xa
0.00
0.02
0.04
0.06
Basal (n=13)
Oryzeae (n=12)
Brachypodieae (n=5)
Triticum (n=5)
Saxifragales (n=4)
Caryophyllales (n=10)
Cornales (n=5)
Campanulids (n=8)
Ericales (n=8)
Gentianales (n=5)
Lamiales (n=11)
Solanales (n=16)
Cucurbitales (n=11)
Fagales (n=11)
Malpighiales (n=15)
Fabales (n=16)
Rosales (n=17)
Myrtales (n=5)
Malvales (n=12)
Sapindales (n=14)
Brassicales (n=33)
Vitales (n=5)

o

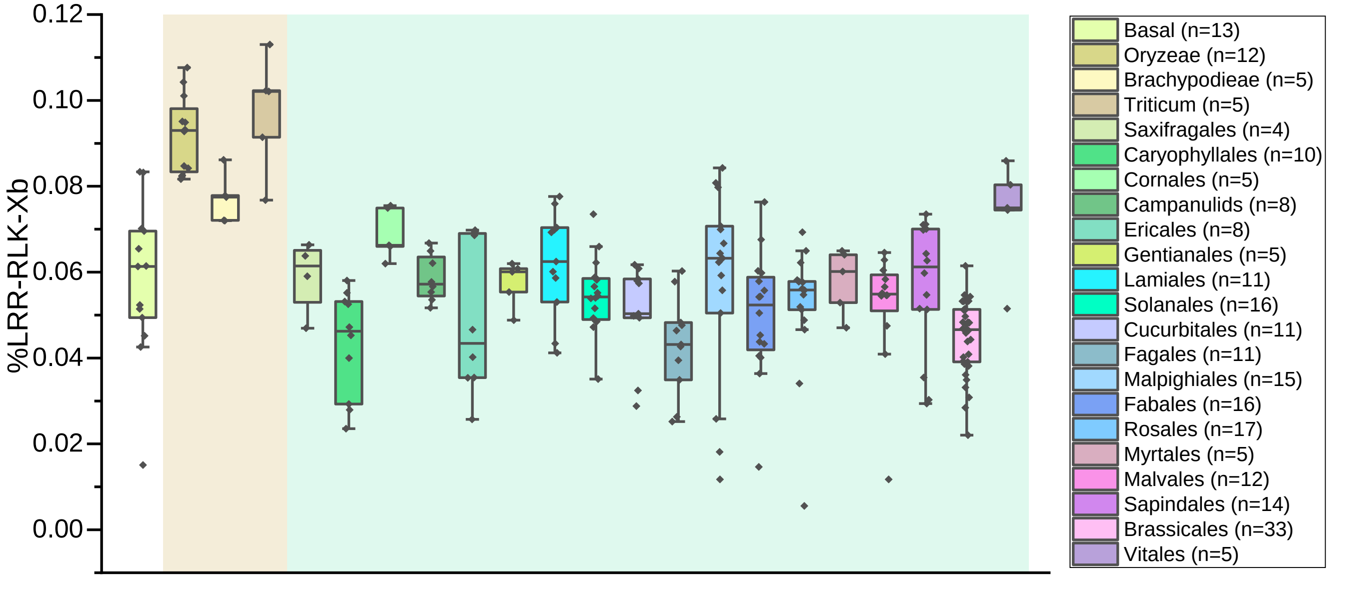
%LRR-RLK-Xb
0.00
0.02
0.04
0.06
0.08
0.10
0.12
Basal (n=13)
Oryzeae (n=12)
Brachypodieae (n=5)
Triticum (n=5)
Saxifragales (n=4)
Caryophyllales (n=10)
Cornales (n=5)
Campanulids (n=8)
Ericales (n=8)
Gentianales (n=5)
Lamiales (n=11)
Solanales (n=16)
Cucurbitales (n=11)
Fagales (n=11)
Malpighiales (n=15)
Fabales (n=16)
Rosales (n=17)
Myrtales (n=5)
Malvales (n=12)
Sapindales (n=14)
Brassicales (n=33)
Vitales (n=5)

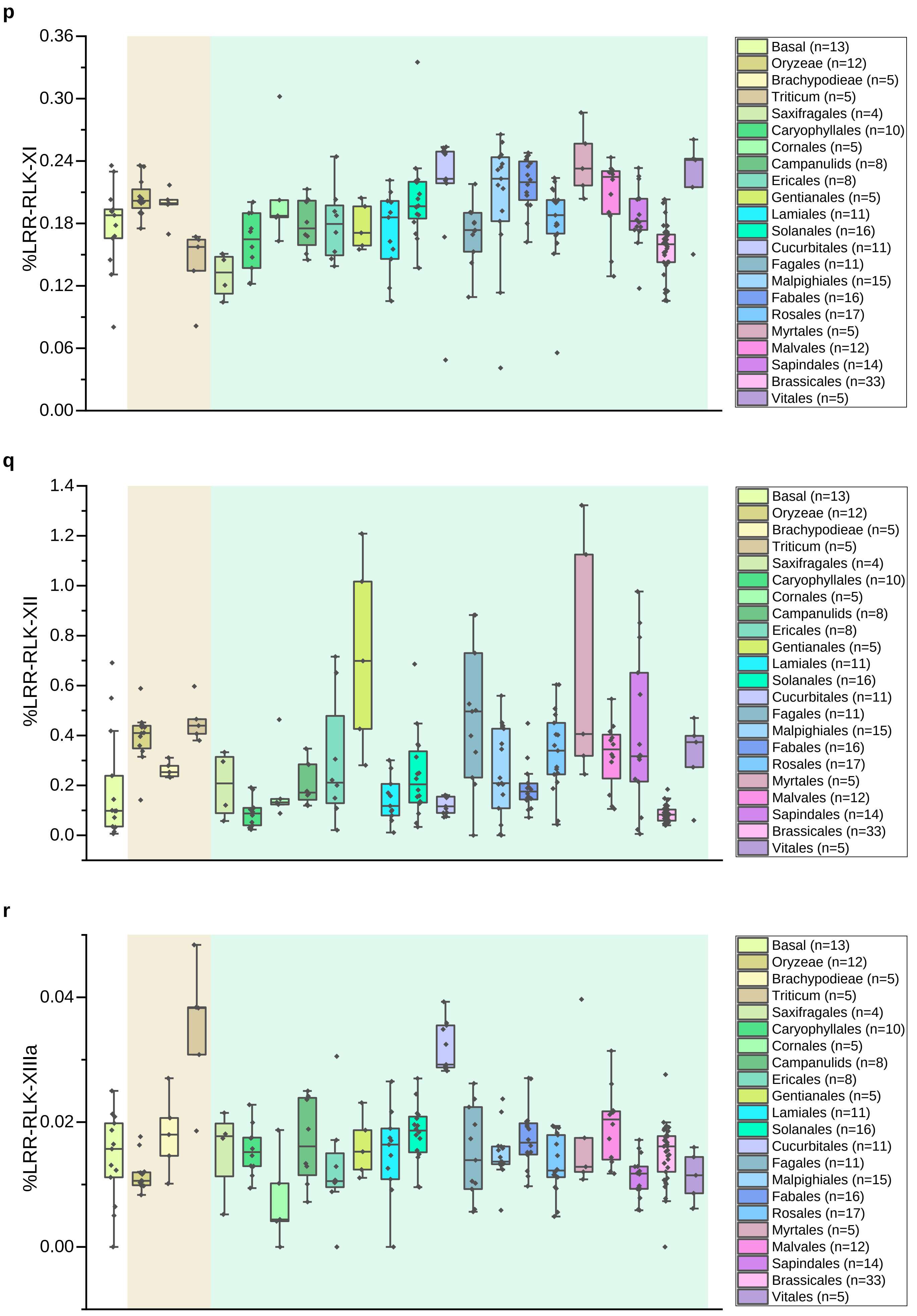
p
%LRR-RLK-XI
0.36
0.30
0.24
0.18
0.12
0.06
0.00
Basal (n=13)
Oryzeae (n=12)
Brachypodieae (n=5)
Triticum (n=5)
Saxifragales (n=4)
Caryophyllales (n=10)
Cornales (n=5)
Campanulids (n=8)
Ericales (n=8)
Gentianales (n=5)
Lamiales (n=11)
Solanales (n=16)
Cucurbitales (n=11)
Fagales (n=11)
Malpighiales (n=15)
Fabales (n=16)
Rosales (n=17)
Myrtales (n=5)
Malvales (n=12)
Sapindales (n=14)
Brassicales (n=33)
Vitales (n=5)
q
%LRR-RLK-XII
1.4
1.2
1.0
0.8
0.6
0.4
0.2
0.0
r
%LRR-RLK-XIIIa
0.04
0.02
0.00

s

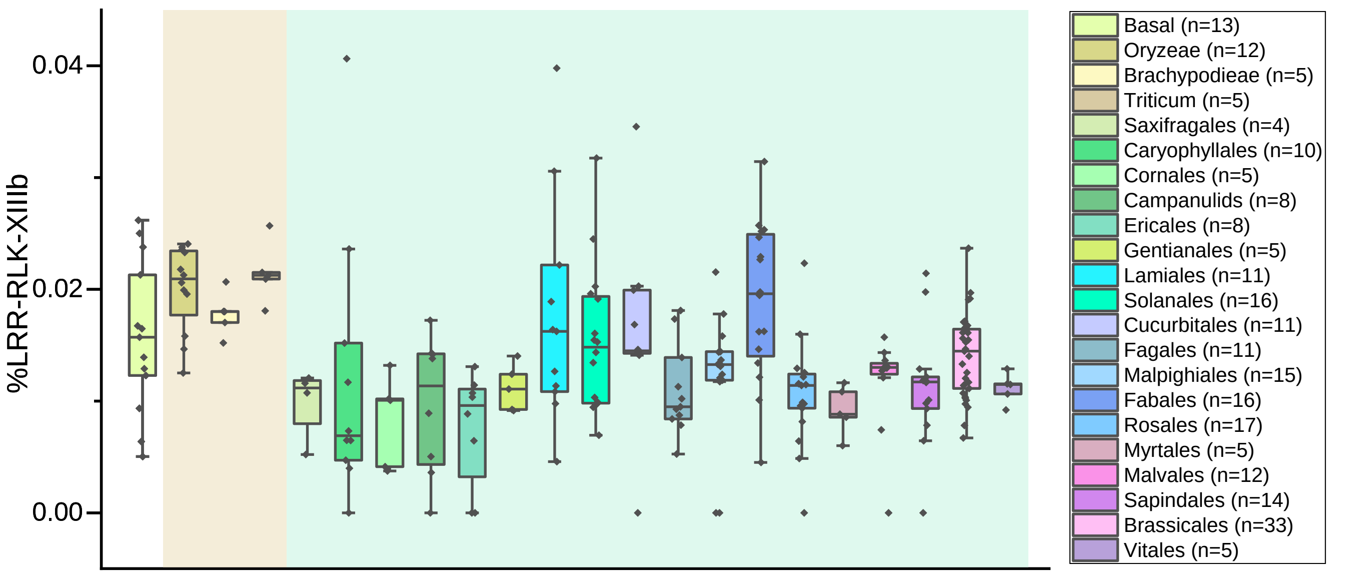

%LRR-RLK-XIIIb
0.04
0.02
0.00
Basal (n=13)
Oryzeae (n=12)
Brachypodieae (n=5)
Triticum (n=5)
Saxifragales (n=4)
Caryophyllales (n=10)
Cornales (n=5)
Campanulids (n=8)
Ericales (n=8)
Gentianales (n=5)
Lamiales (n=11)
Solanales (n=16)
Cucurbitales (n=11)
Fagales (n=11)
Malpighiales (n=15)
Fabales (n=16)
Rosales (n=17)
Myrtales (n=5)
Malvales (n=12)
Sapindales (n=14)
Brassicales (n=33)
Vitales (n=5)

t

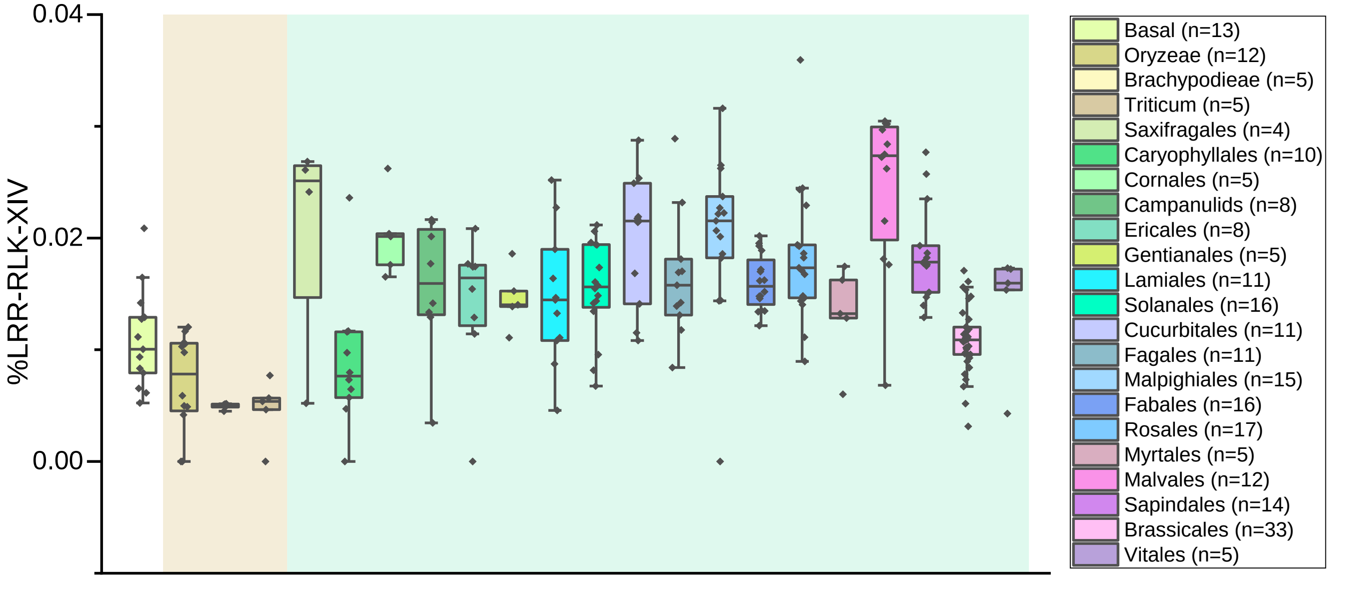

%LRR-RLK-XIV
0.04
0.02
0.00
Basal (n=13)
Oryzeae (n=12)
Brachypodieae (n=5)
Triticum (n=5)
Saxifragales (n=4)
Caryophyllales (n=10)
Cornales (n=5)
Campanulids (n=8)
Ericales (n=8)
Gentianales (n=5)
Lamiales (n=11)
Solanales (n=16)
Cucurbitales (n=11)
Fagales (n=11)
Malpighiales (n=15)
Fabales (n=16)
Rosales (n=17)
Myrtales (n=5)
Malvales (n=12)
Sapindales (n=14)
Brassicales (n=33)
Vitales (n=5)

u

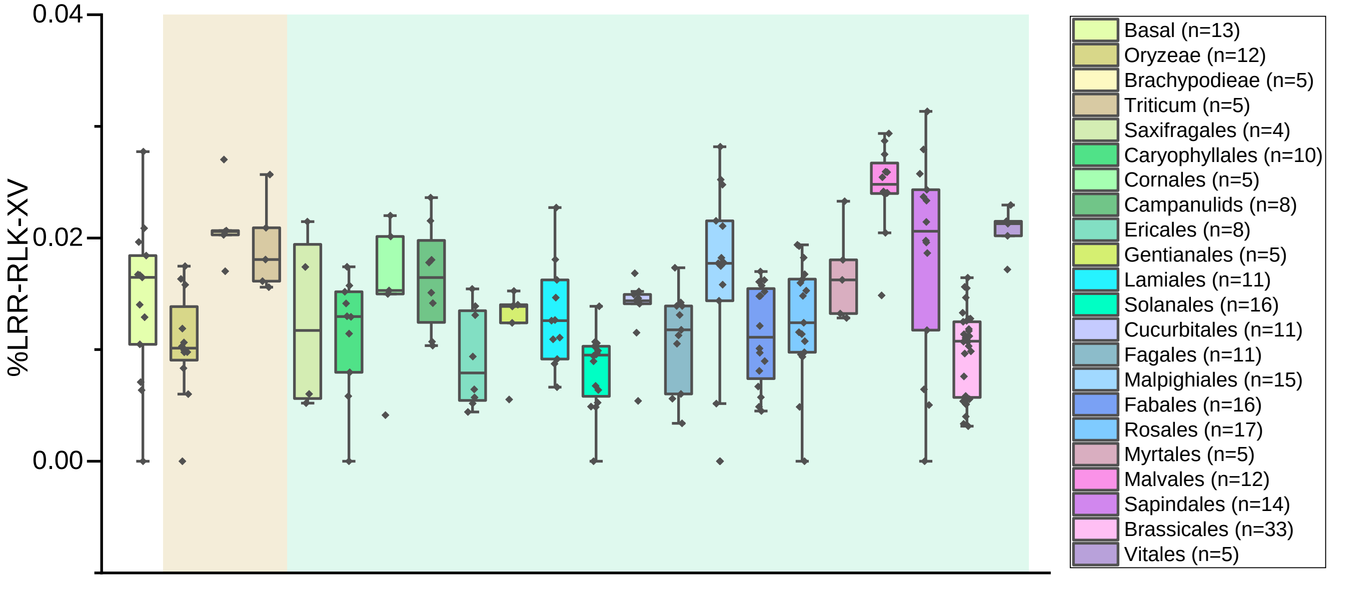

%LRR-RLK-XV
0.04
0.02
0.00
Basal (n=13)
Oryzeae (n=12)
Brachypodieae (n=5)
Triticum (n=5)
Saxifragales (n=4)
Caryophyllales (n=10)
Cornales (n=5)
Campanulids (n=8)
Ericales (n=8)
Gentianales (n=5)
Lamiales (n=11)
Solanales (n=16)
Cucurbitales (n=11)
Fagales (n=11)
Malpighiales (n=15)
Fabales (n=16)
Rosales (n=17)
Myrtales (n=5)
Malvales (n=12)
Sapindales (n=14)
Brassicales (n=33)
Vitales (n=5)

v

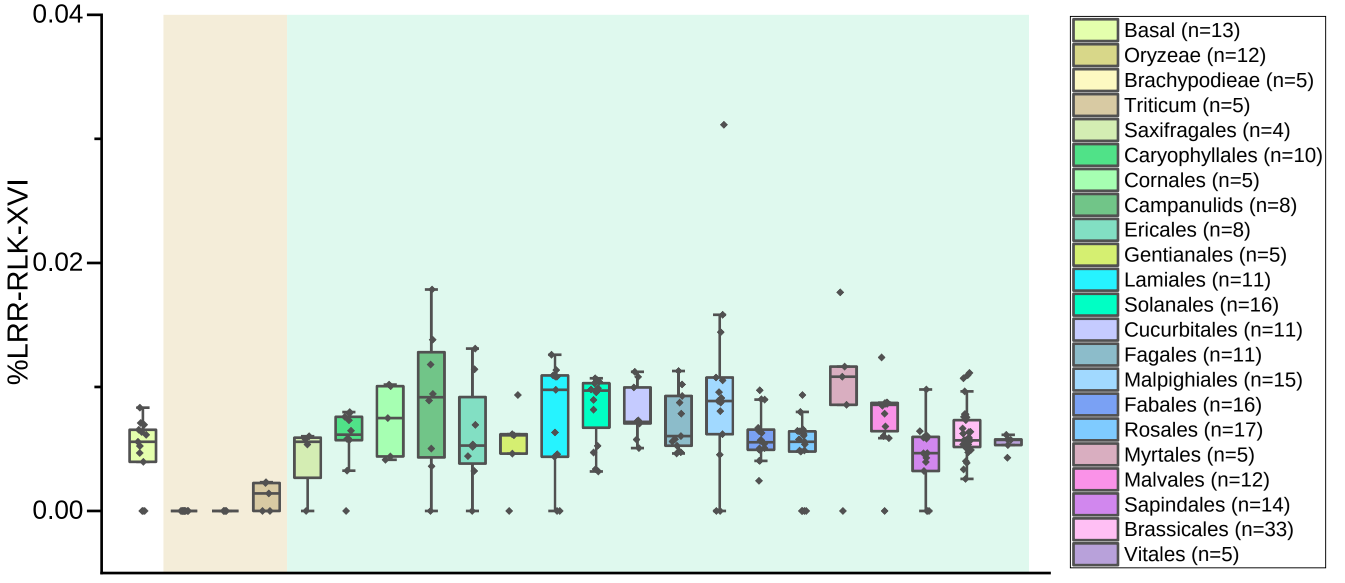
0.04
0.02
0.00
%LRR-RLK-XVI
Basal (n=13)
Oryzeae (n=12)
Brachypodieae (n=5)
Triticum (n=5)
Saxifragales (n=4)
Caryophyllales (n=10)
Cornales (n=5)
Campanulids (n=8)
Ericales (n=8)
Gentianales (n=5)
Lamiales (n=11)
Solanales (n=16)
Cucurbitales (n=11)
Fagales (n=11)
Malpighiales (n=15)
Fabales (n=16)
Rosales (n=17)
Myrtales (n=5)
Malvales (n=12)
Sapindales (n=14)
Brassicales (n=33)
Vitales (n=5)

w

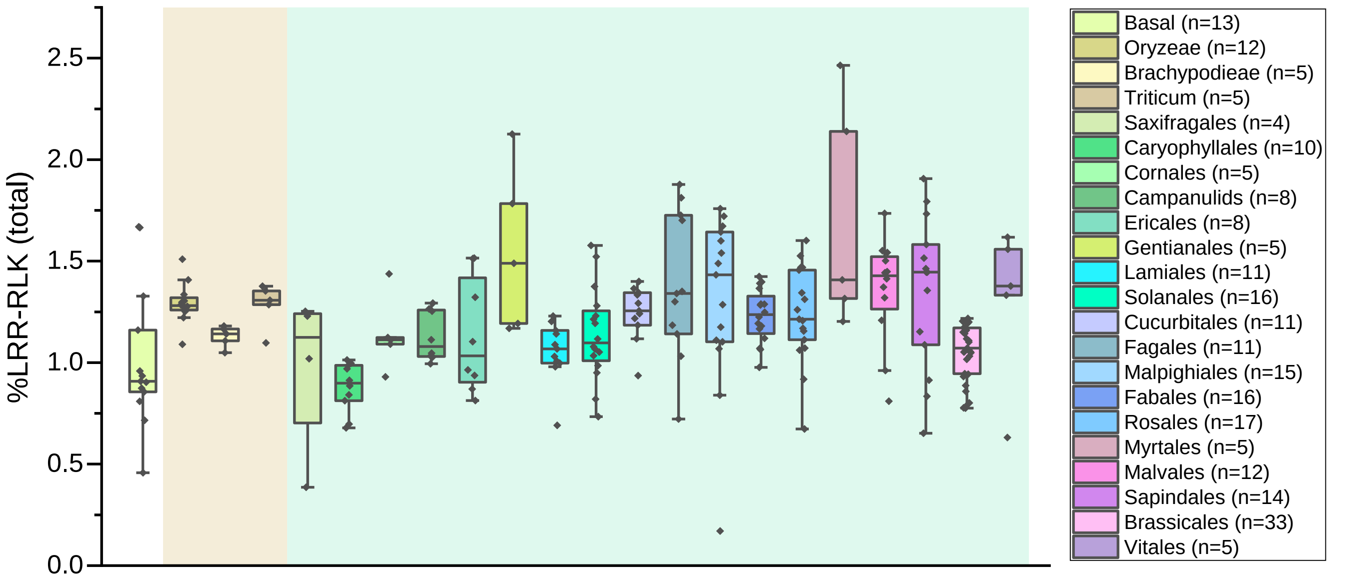
2.5
2.0
1.5
1.0
0.5
0.0
%LRR-RLK (total)
Basal (n=13)
Oryzeae (n=12)
Brachypodieae (n=5)
Triticum (n=5)
Saxifragales (n=4)
Caryophyllales (n=10)
Cornales (n=5)
Campanulids (n=8)
Ericales (n=8)
Gentianales (n=5)
Lamiales (n=11)
Solanales (n=16)
Cucurbitales (n=11)
Fagales (n=11)
Malpighiales (n=15)
Fabales (n=16)
Rosales (n=17)
Myrtales (n=5)
Malvales (n=12)
Sapindales (n=14)
Brassicales (n=33)
Vitales (n=5)

x

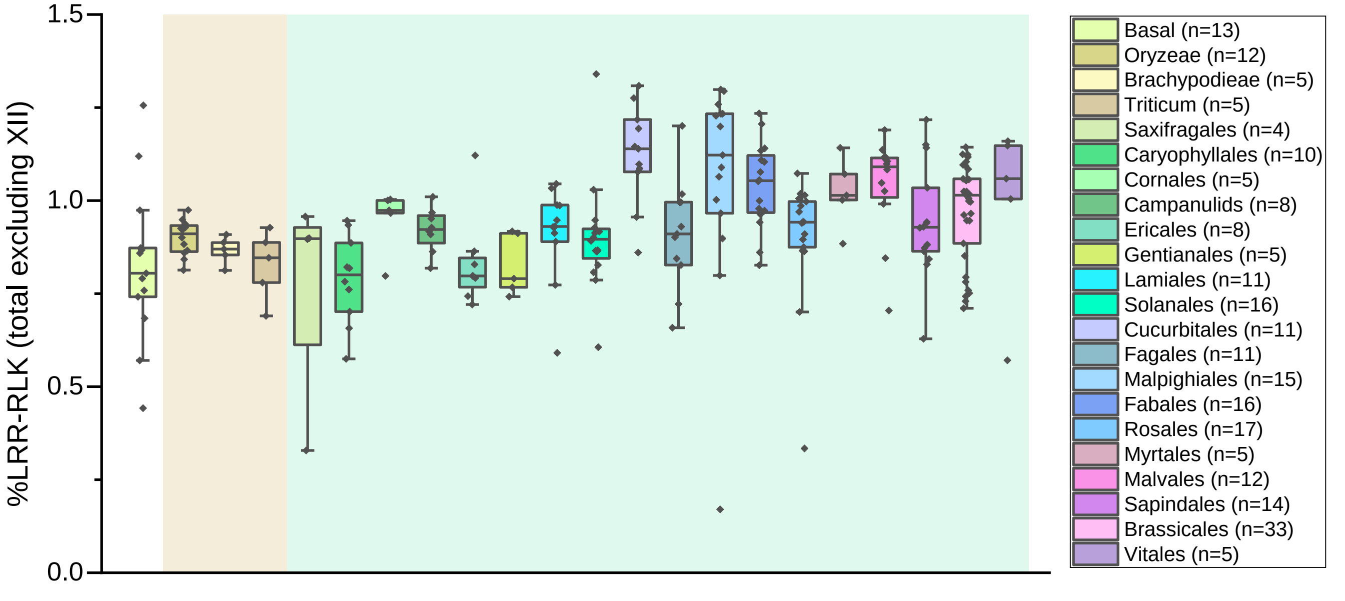
1.5
1.0
0.5
0.0
%LRR-RLK (total excluding XII)
Basal (n=13)
Oryzeae (n=12)
Brachypodieae (n=5)
Triticum (n=5)
Saxifragales (n=4)
Caryophyllales (n=10)
Cornales (n=5)
Campanulids (n=8)
Ericales (n=8)
Gentianales (n=5)
Lamiales (n=11)
Solanales (n=16)
Cucurbitales (n=11)
Fagales (n=11)
Malpighiales (n=15)
Fabales (n=16)
Rosales (n=17)
Myrtales (n=5)
Malvales (n=12)
Sapindales (n=14)
Brassicales (n=33)
Vitales (n=5)

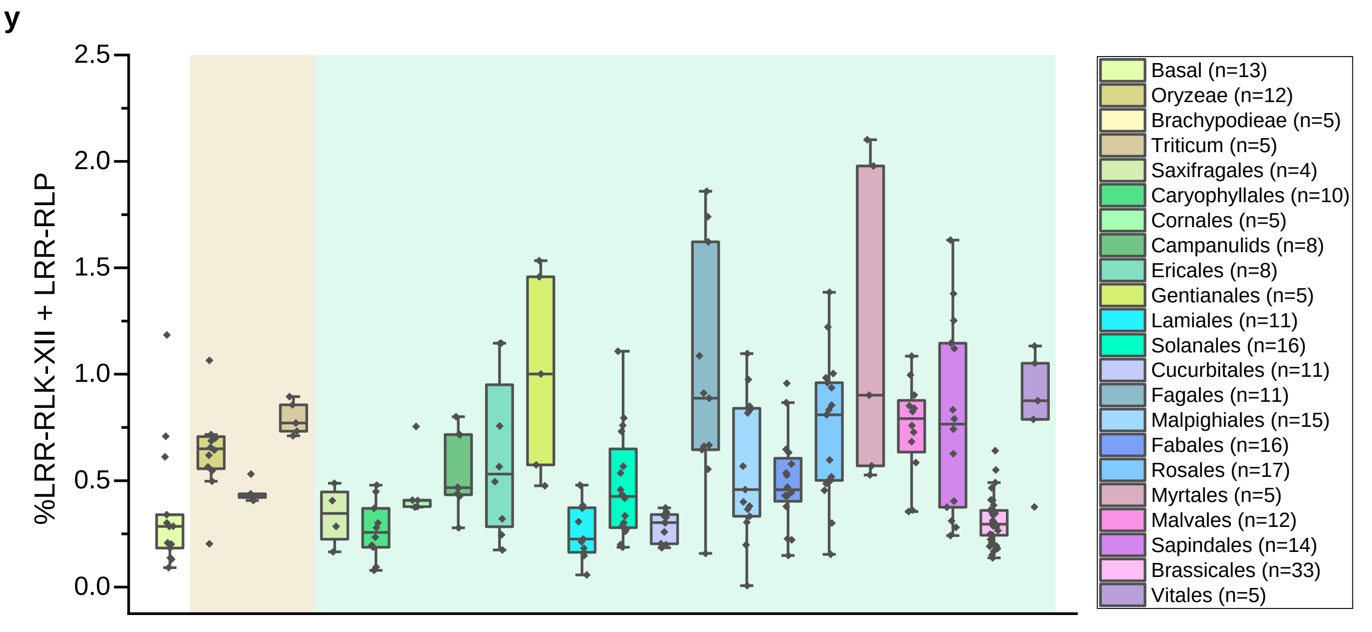
y
2.5
2.0
1.5
1.0
0.5
0.0
%LRR-RLK-XII + LRR-RLP
Basal (n=13)
Oryzeae (n=12)
Brachypodieae (n=5)
Triticum (n=5)
Saxifragales (n=4)
Caryophyllales (n=10)
Cornales (n=5)
Campanulids (n=8)
Ericales (n=8)
Gentianales (n=5)
Lamiales (n=11)
Solanales (n=16)
Cucurbitales (n=11)
Fagales (n=11)
Malpighiales (n=15)
Fabales (n=16)
Rosales (n=17)
Myrtales (n=5)
Malvales (n=12)
Sapindales (n=14)
Brassicales (n=33)
Vitales (n=5)

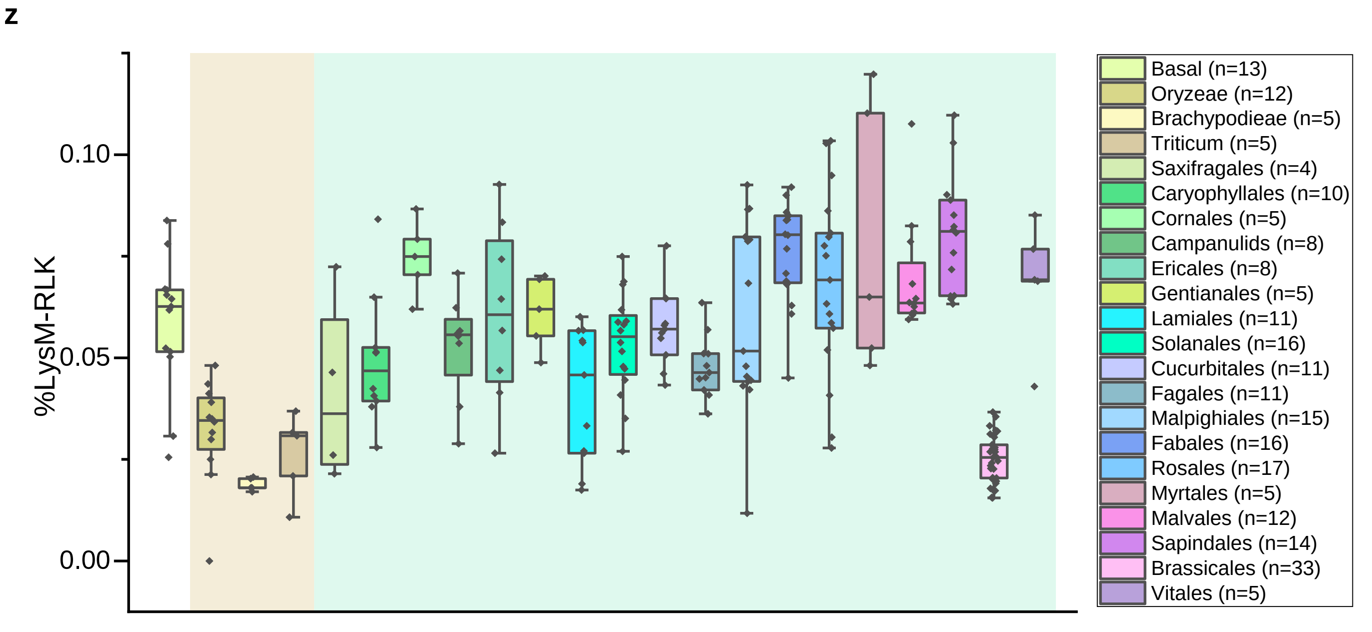
z
0.10
0.05
0.00
%LysM-RLK
Basal (n=13)
Oryzeae (n=12)
Brachypodieae (n=5)
Triticum (n=5)
Saxifragales (n=4)
Caryophyllales (n=10)
Cornales (n=5)
Campanulids (n=8)
Ericales (n=8)
Gentianales (n=5)
Lamiales (n=11)
Solanales (n=16)
Cucurbitales (n=11)
Fagales (n=11)
Malpighiales (n=15)
Fabales (n=16)
Rosales (n=17)
Myrtales (n=5)
Malvales (n=12)
Sapindales (n=14)
Brassicales (n=33)
Vitales (n=5)

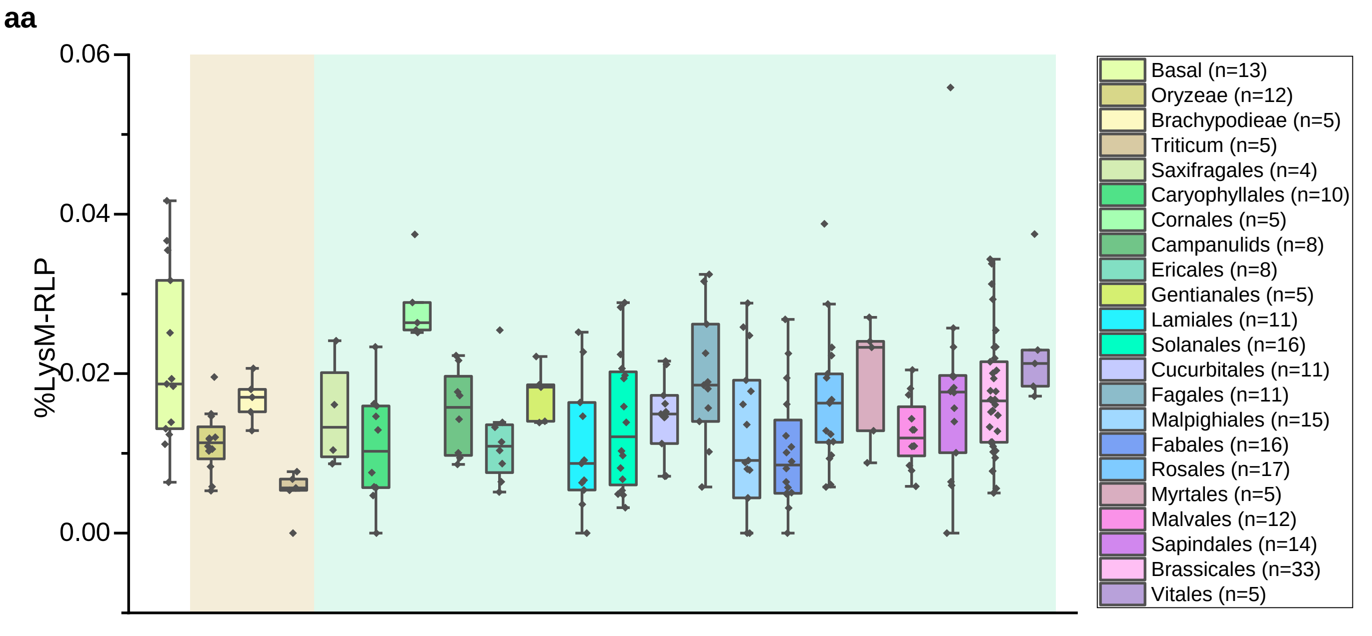
aa
0.06
0.04
0.02
0.00
%LysM-RLP
Basal (n=13)
Oryzeae (n=12)
Brachypodieae (n=5)
Triticum (n=5)
Saxifragales (n=4)
Caryophyllales (n=10)
Cornales (n=5)
Campanulids (n=8)
Ericales (n=8)
Gentianales (n=5)
Lamiales (n=11)
Solanales (n=16)
Cucurbitales (n=11)
Fagales (n=11)
Malpighiales (n=15)
Fabales (n=16)
Rosales (n=17)
Myrtales (n=5)
Malvales (n=12)
Sapindales (n=14)
Brassicales (n=33)
Vitales (n=5)

Supplementary figure 4. Number of receptor gene families in each plant groups. Boxplots represent the percentage (%) of receptor genes (a, NB-ARC; b, LRR-RLP; c, LRR-RLK-I; d, LRR-RLK-II; e, LRR-RLK-III; f, LRR-RLK-IV; g, LRR-RLK-V; h, LRR-RLK-VI_1; i, LRR-RLK-VI_2; j, LRR-RLK-VII; k, LRR-RLK-VIII_1; l, LRR-RLK-VIII_2; m, LRR-RLK-IX; n, LRR-RLK-Xa; o, LRR-RLK-Xb; p, LRR-RLK-XI; q, LRR-RLK-XII; r, LRR-RLK-XIIIa; s, LRR-RLK-XIIIb; t, LRR-RLK-XIV; u, LRR-RLK-XV; v, LRR-RLK-XVI; w, LRR-RLK (total); x, LRR-RLK (total except LRR-RLK-XII); y, LRR-RLK-XII + LRR-RLP; z, LysM-RLK; aa, LysM-RLP) in different plant groups. Brown shade represents monocots and teal shade represents eudicots. Box-plot elements: centre line, median; bounds of box, 25th and 75th percentiles; whiskers, 1.5 × IQR from 25th and 75th percentiles.

Supplementary figure 5

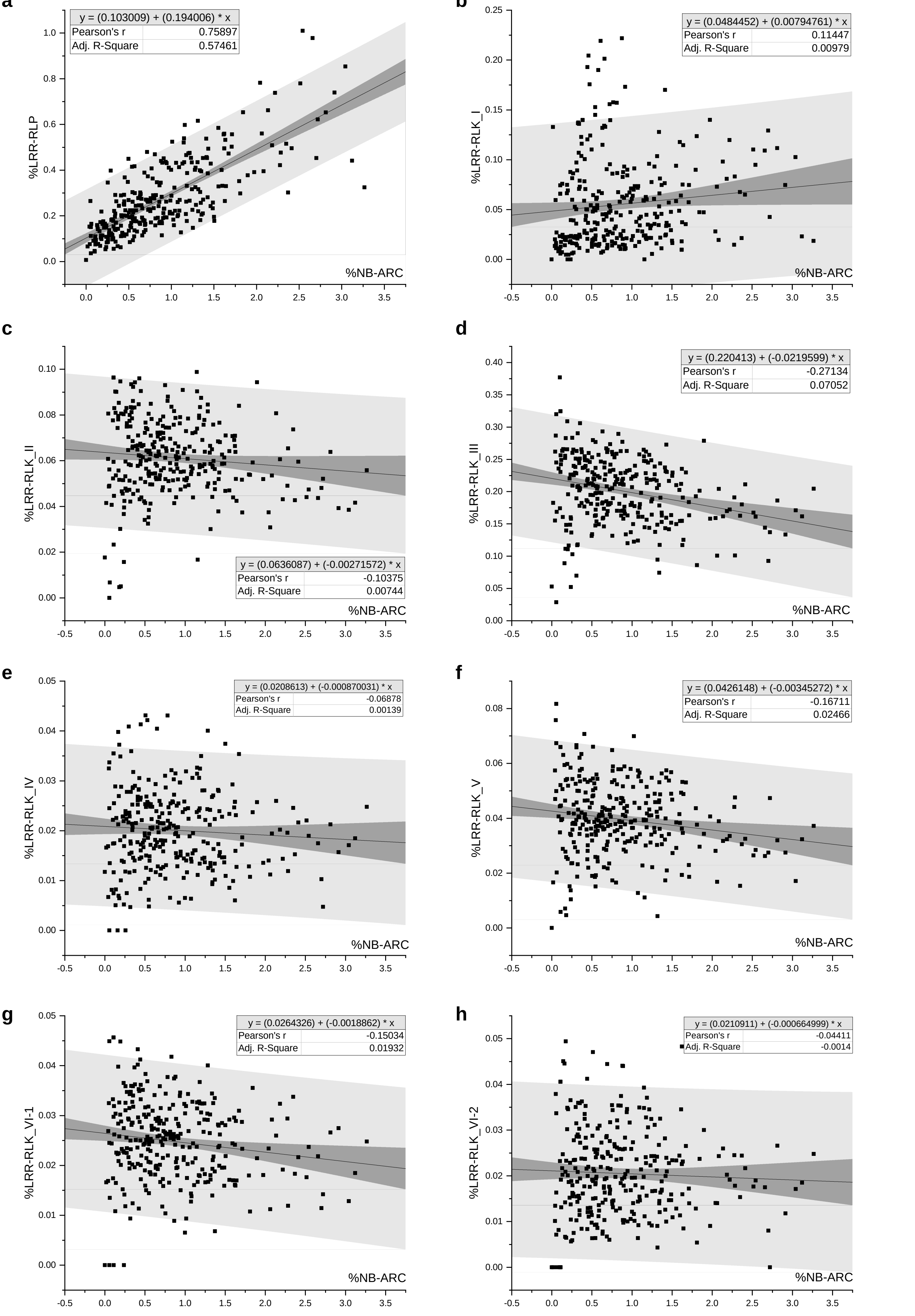

Supplementary figure 5

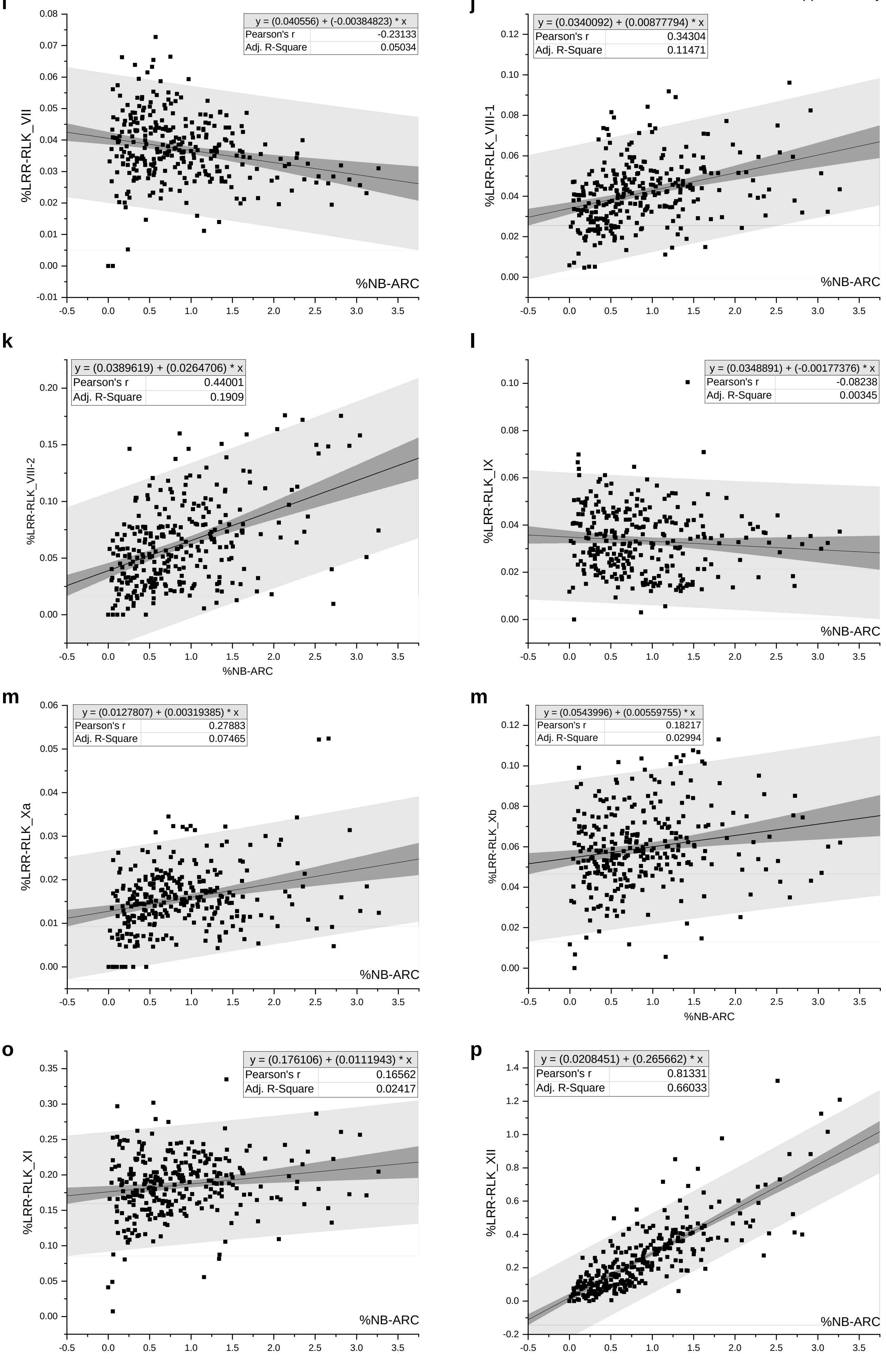

Supplementary figure 5

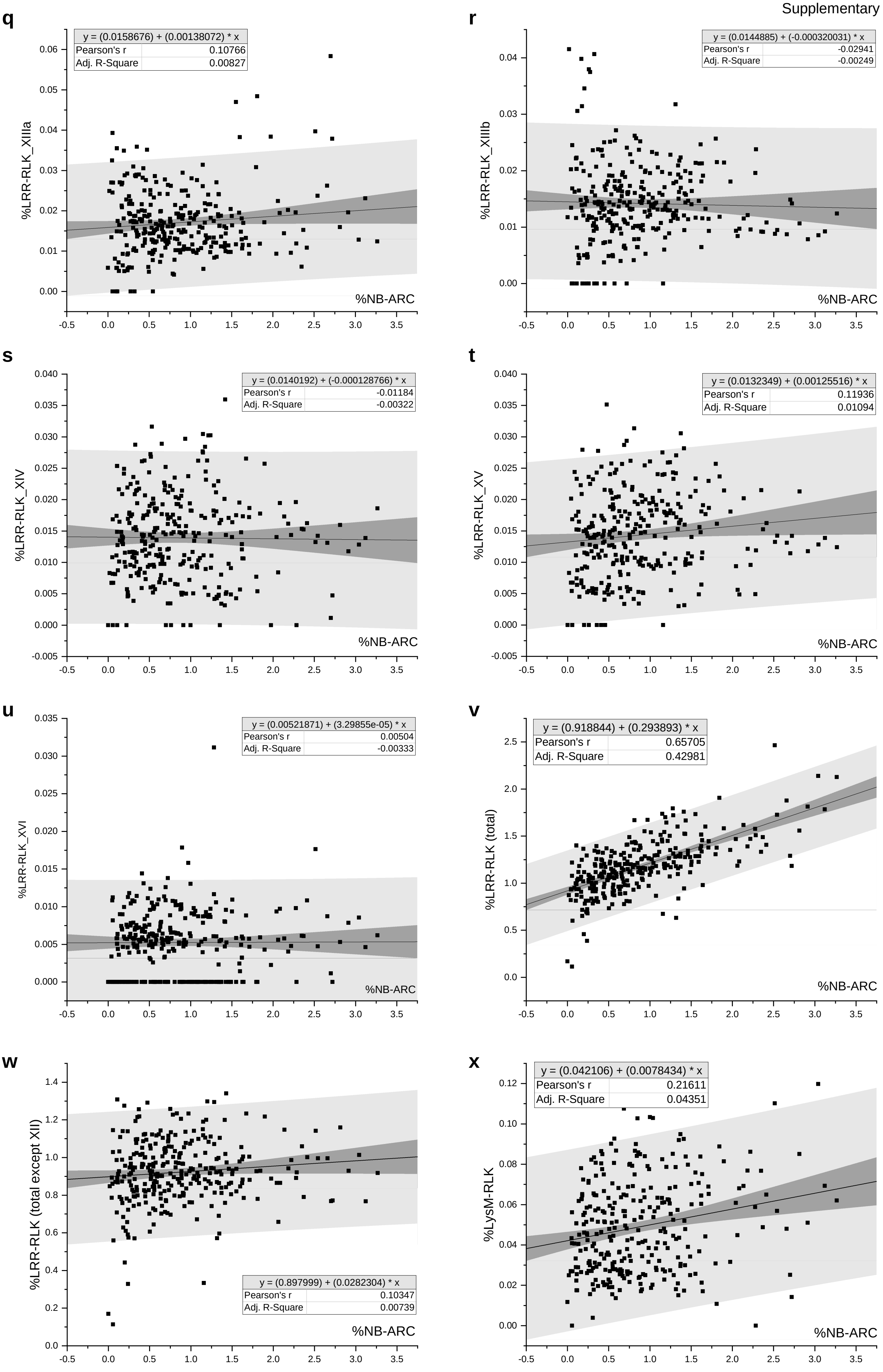

y

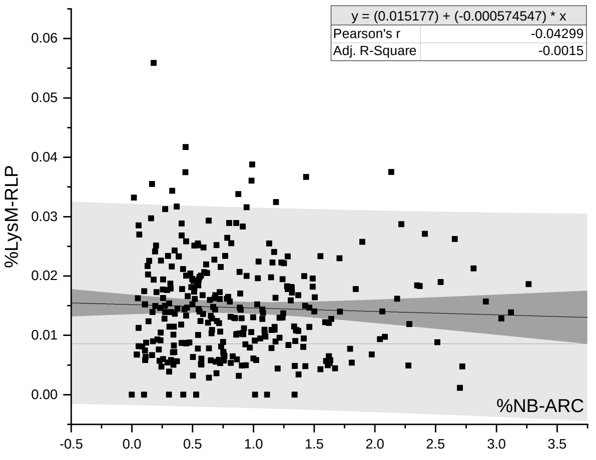

Supplementary figure 5. Correlation between percentage (%) NB-ARC and other receptor gene families in 300 angiosperm genomes. Scatter plot of % receptor gene families (a, LRR-RLP; b, LRR-RLK-I; c, LRR-RLK-II; d, LRR-RLK-III; e, LRR-RLK-IV; f, LRR-RLK-V; g, LRR-RLK-VI_1; h, LRR-RLK-VI_2; i, LRR-RLK-VII; j, LRR-RLK-VIII_1; k, LRR-RLK-VIII_2; l, LRR-RLK-IX; m, LRR-RLK-Xa; n, LRR-RLK-Xb; o, LRR-RLK-XI; p, LRR-RLK-XII; q, LRR-RLK-XIIIa; r, LRR-RLK-XIIIb; s, LRR-RLK-XIV; t, LRR-RLK-XV; u, LRR-RLK-XVI; v, LRR-RLK (total); w, LRR-RLK (excluding LRR-RLK-XII), x, LysM-RLK; y, LysM-RLP against % NB-ARC. Pearson correlation coefficient and adjusted r-square values are indicated. Black line represents the linear trend. Dark grey shade represents 95% confidence band and light grey shade represents 95% prediction band.

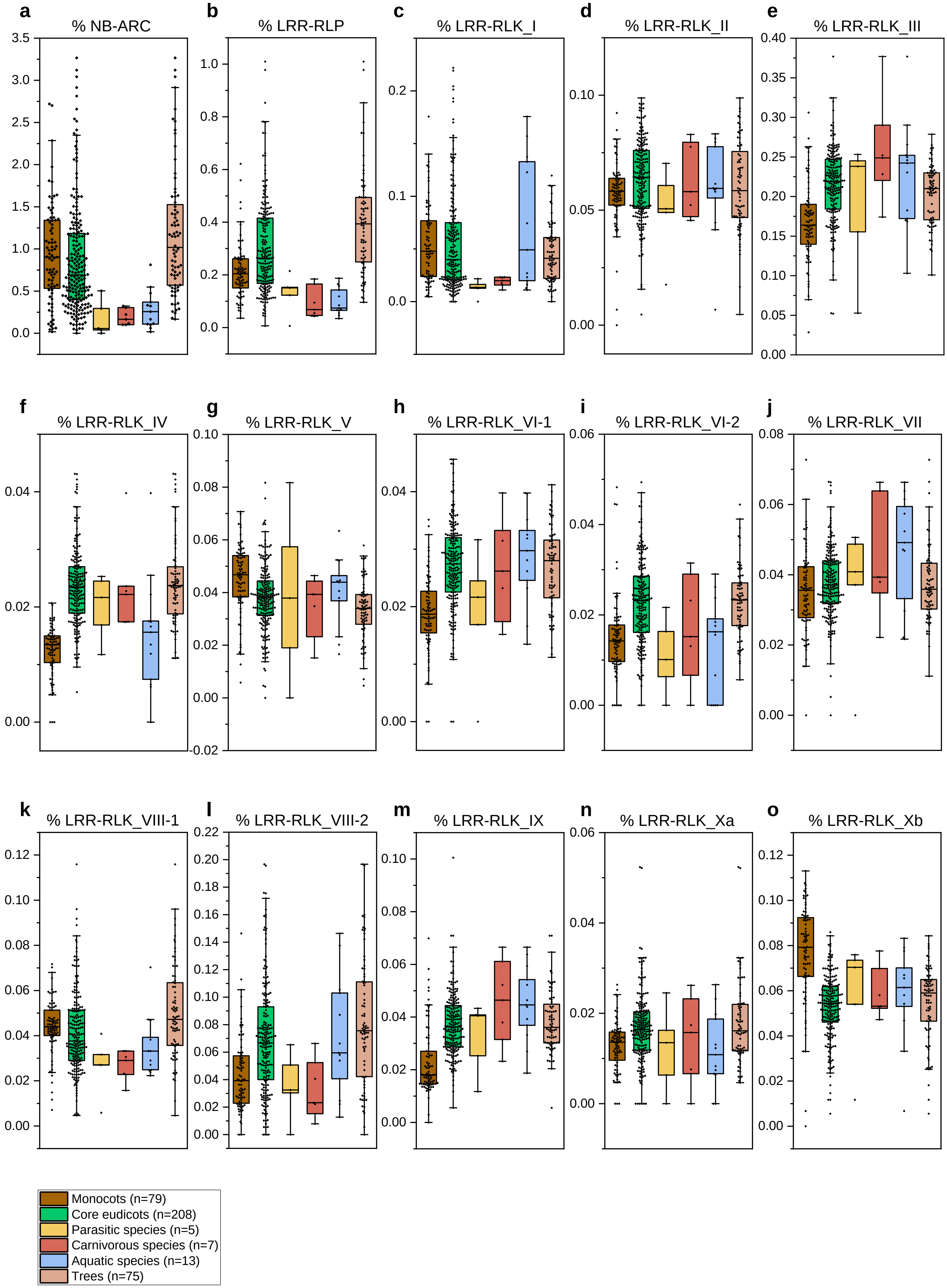
a
% NB-ARC
b
% LRR-RLP
c
% LRR-RLK_I
d
% LRR-RLK_II
e
% LRR-RLK_III
f
% LRR-RLK_IV
g
% LRR-RLK_V
h
% LRR-RLK_VI-1
i
% LRR-RLK_VI-2
j
% LRR-RLK_VII
k
% LRR-RLK_VIII-1
l
% LRR-RLK_VIII-2
m
% LRR-RLK_IX
n
% LRR-RLK_Xa
o
% LRR-RLK_Xb
Monocots (n=79)
Core eudicots (n=208)
Parasitic species (n=5)
Carnivorous species (n=7)
Aquatic species (n=13)
Trees (n=75)

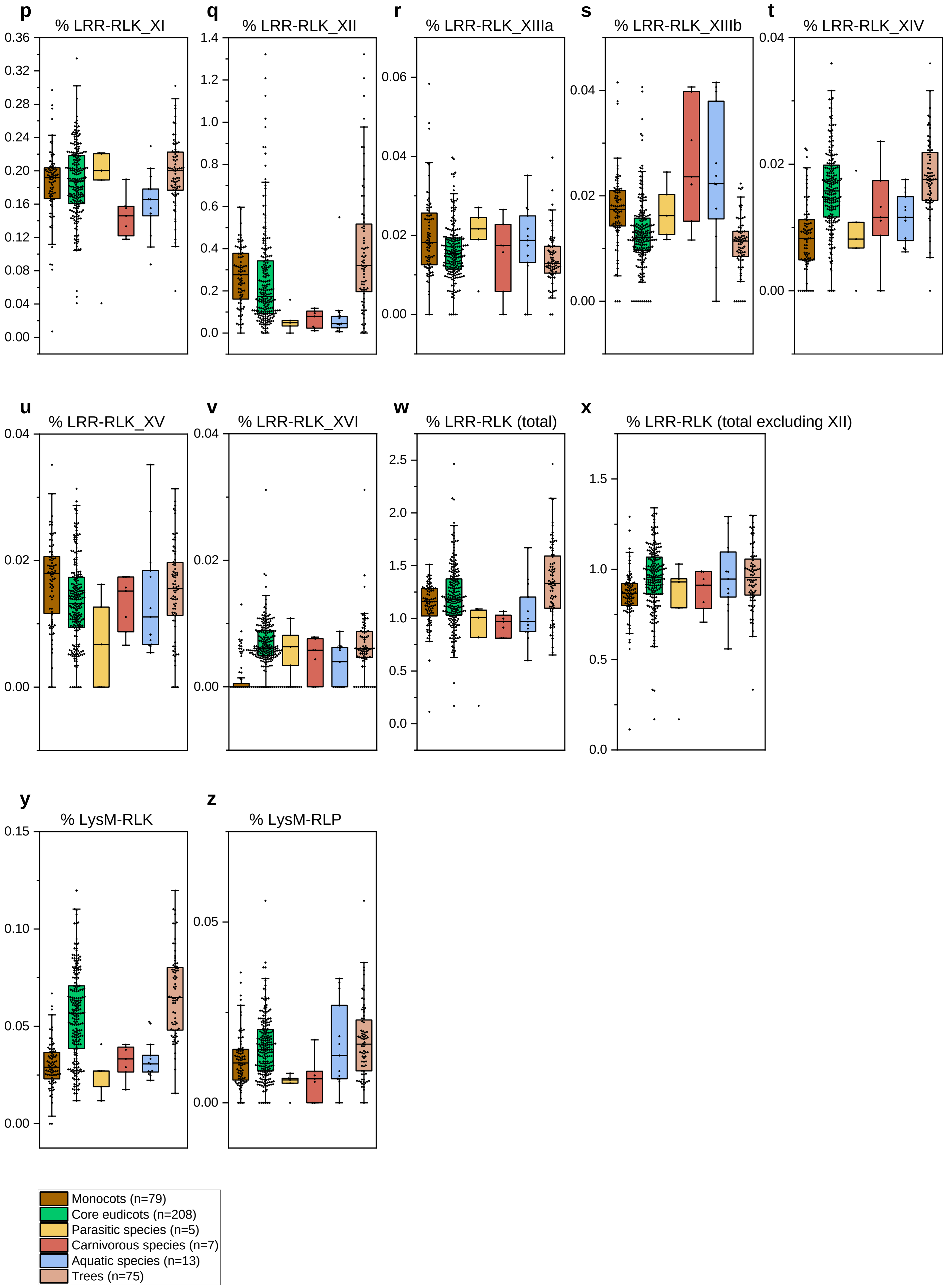
p
% LRR-RLK_XI
0.36
0.32
0.28
0.24
0.20
0.16
0.12
0.08
0.04
0.00
q
% LRR-RLK_XII
1.4
1.2
1.0
0.8
0.6
0.4
0.2
0.0
r
% LRR-RLK_XIIIa
0.06
0.04
0.02
0.00
s
% LRR-RLK_XIIIb
0.04
0.02
0.00
t
% LRR-RLK_XIV
0.04
0.02
0.00
u
% LRR-RLK_XV
0.04
0.02
0.00
v
% LRR-RLK_XVI
0.04
0.02
0.00
w
% LRR-RLK (total)
2.5
2.0
1.5
1.0
0.5
0.0
x
% LRR-RLK (total excluding XII)
1.5
1.0
0.5
0.0
y
% LysM-RLK
0.15
0.10
0.05
0.00
z
% LysM-RLP
0.05
0.00
Monocots (n=79)
Core eudicots (n=208)
Parasitic species (n=5)
Carnivorous species (n=7)
Aquatic species (n=13)
Trees (n=75)

Supplementary figure 6. Number of receptor gene families in monocots, eudicots, parasitic species, carnivorous species, aquatic species and trees. Boxplots represent the percentage (%) of receptor genes (a, NB-ARC; b, LRR-RLP; c, LRR-RLK-I; d, LRR-RLK-II; e, LRR-RLK-III; f, LRR-RLK-IV; g, LRR-RLK-V; h, LRR-RLK-VI_1; i, LRR-RLK-VI_2; j, LRR-RLK-VII; k, LRR-RLK-VIII_1; l, LRR-RLK-VIII_2; m, LRR-RLK-IX; n, LRR-RLK-Xa; o, LRR-RLK-Xb; p, LRR-RLK-XI; q, LRR-RLK-XII; r, LRR-RLK-XIIIa; s, LRR-RLK-XIIIb; t, LRR-RLK-XIV; u, LRR-RLK-XV; v, LRR-RLK-XVI; w, LRR-RLK (total); x, LRR-RLKs (total excluding LRR-RLK-XII); y, LysM-RLK; z, LysM-RLP) in monocots, eudicots, parasitic species, carnivorous species, aquatic species and trees. Box-plot elements: centre line, median; bounds of box, 25th and 75th percentiles; whiskers, 1.5 × IQR from 25th and 75th percentiles.

a

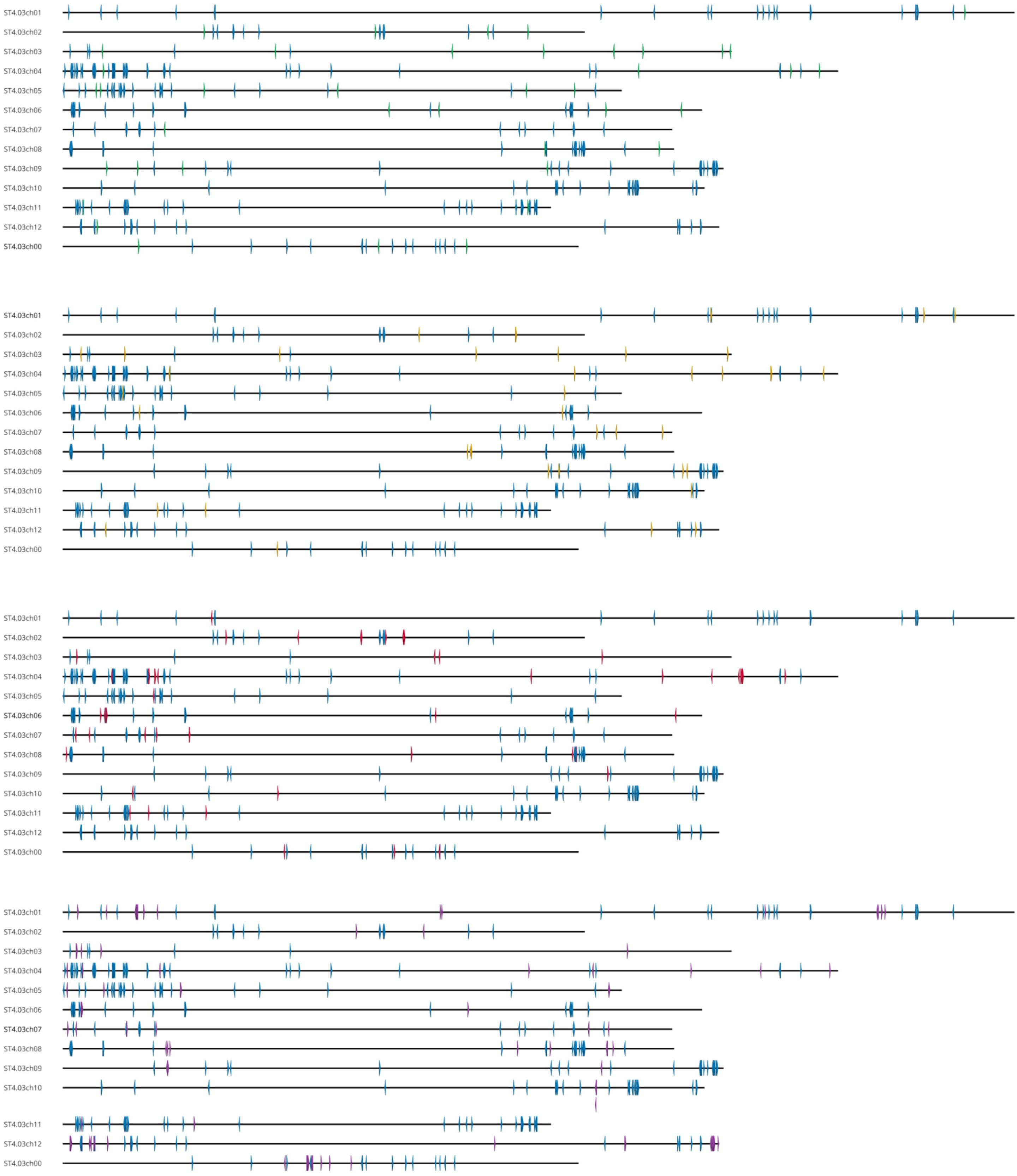
ST4.03ch01
ST4.03ch02
ST4.03ch03
ST4.03ch04
ST4.03ch05
ST4.03ch06
ST4.03ch07
ST4.03ch08
ST4.03ch09
ST4.03ch10
ST4.03ch11
ST4.03ch12
ST4.03ch00

b

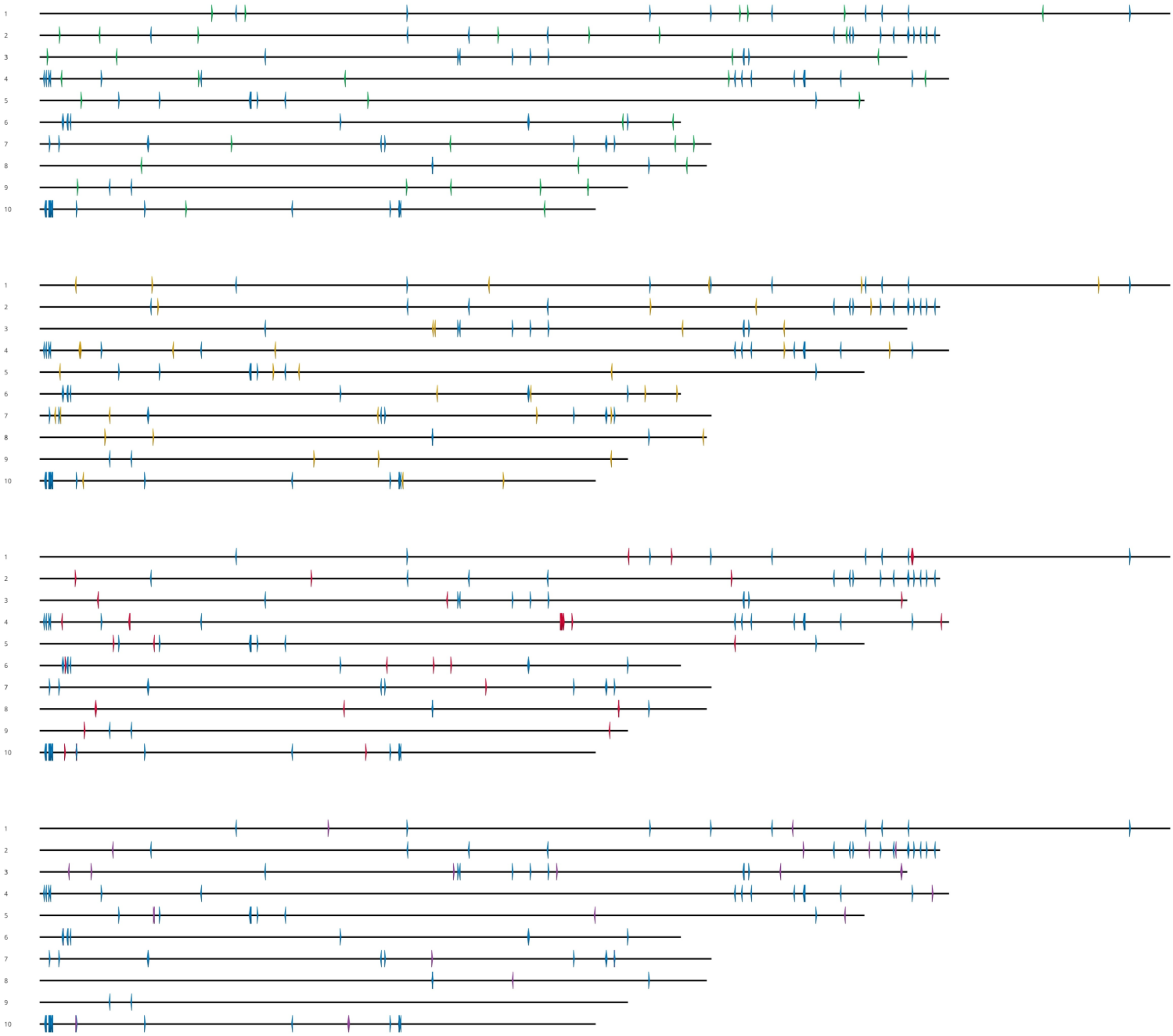

c

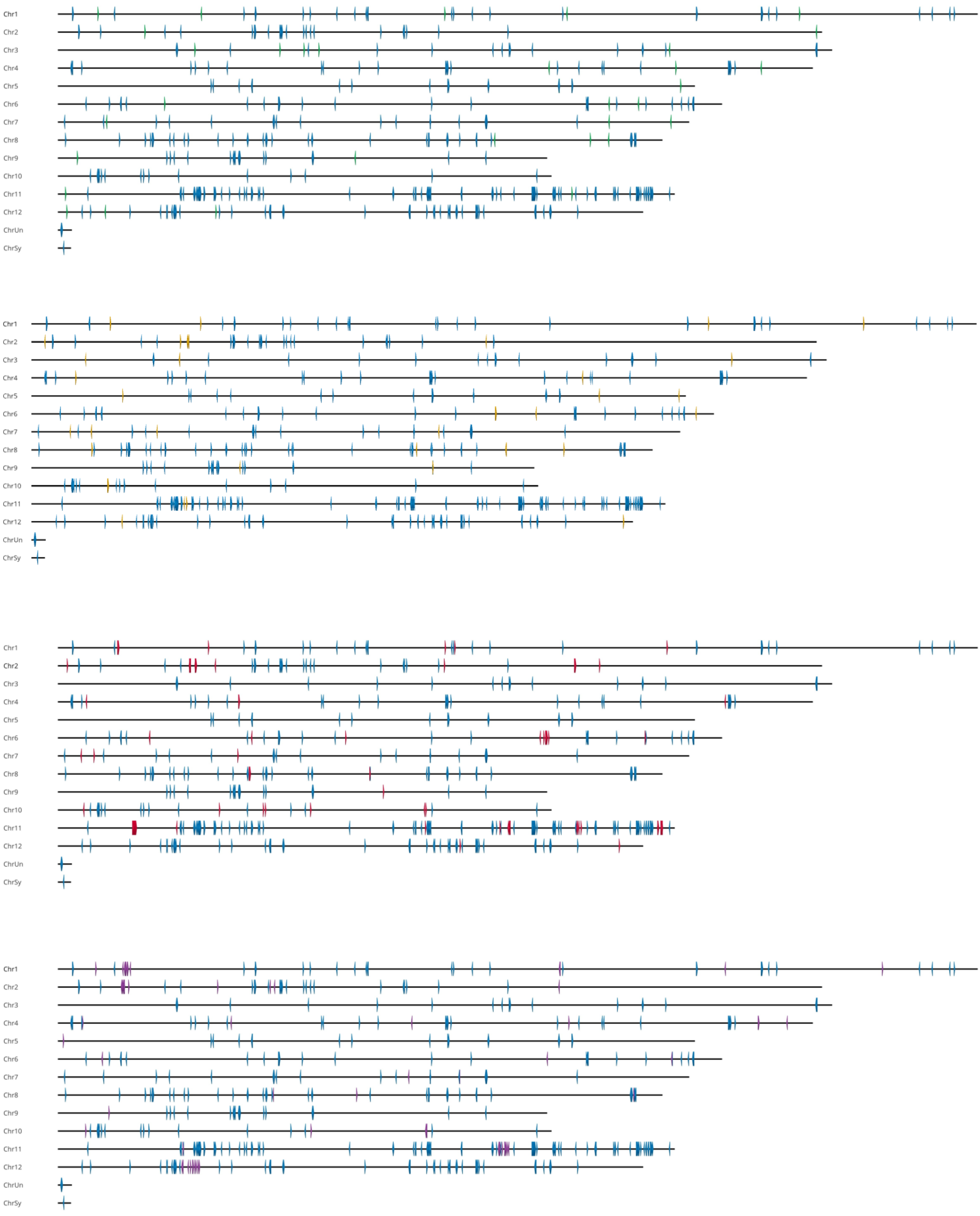
Chr1
Chr2
Chr3
Chr4
Chr5
Chr6
Chr7
Chr8
Chr9
Chr10
Chr11
Chr12
ChrUn
ChrSy
Chr1
Chr2
Chr3
Chr4
Chr5
Chr6
Chr7
Chr8
Chr9
Chr10
Chr11
Chr12
ChrUn
ChrSy
Chr1
Chr2
Chr3
Chr4
Chr5
Chr6
Chr7
Chr8
Chr9
Chr10
Chr11
Chr12
ChrUn
ChrSy
Chr1
Chr2
Chr3
Chr4
Chr5
Chr6
Chr7
Chr8
Chr9
Chr10
Chr11
Chr12
ChrUn
ChrSy

Supplementary figure 7. Genes encoding NB-ARCs, LRR-RLKs and LRR-RLPs co-localise in the tomato, potato and rice genomes. Maps of a) *S. tuberosum* , b) *Z.mays* and c) *O. sativa* genomes showing the location of genes encoding NB-ARC proteins (in blue) and their co-localisation with LRR-RLKs (subgroup III in green; subgroup XI in yellow and subgroup XII in red) and LRR-RLP (in purple) encoding proteins.
