# Supplementary material for: Concerted expansion and contraction of immune receptor gene repertoires in plant genomes
Source: Nat Plants. 2022 Oct 14;8(10):1146–52. doi: 10.1038/s41477-022-01260-5 (PMC9579050; doi:10.1038/s41477-022-01260-5)
Supplement: Supplementary file 2 — Reporting Summary [file 41477_2022_1260_MOESM2_ESM.pdf]

nature portfolio

Corresponding author(s): Jonathan DG Jones

Last updated by author(s): Aug 26, 2022

Reporting Summary

Nature Portfolio wishes to improve the reproducibility of the work that we publish. This form provides structure for consistency and transparency in reporting. For further information on Nature Portfolio policies, see our Editorial Policies and the Editorial Policy Checklist.

Statistics

For all statistical analyses, confirm that the following items are present in the figure legend, table legend, main text, or Methods section.

n/a	Confirmed	
☐	☒	The exact sample size (*n*) for each experimental group/condition, given as a discrete number and unit of measurement
☐	☒	A statement on whether measurements were taken from distinct samples or whether the same sample was measured repeatedly
☐	☒	The statistical test(s) used AND whether they are one- or two-sided<br>*Only common tests should be described solely by name; describe more complex techniques in the Methods section.*
☒	☐	A description of all covariates tested
☐	☒	A description of any assumptions or corrections, such as tests of normality and adjustment for multiple comparisons
☐	☒	A full description of the statistical parameters including central tendency (e.g. means) or other basic estimates (e.g. regression coefficient) AND variation (e.g. standard deviation) or associated estimates of uncertainty (e.g. confidence intervals)
☐	☒	For null hypothesis testing, the test statistic (e.g. *F*, *t*, *r*) with confidence intervals, effect sizes, degrees of freedom and *P* value noted<br>*Give P values as exact values whenever suitable.*
☒	☐	For Bayesian analysis, information on the choice of priors and Markov chain Monte Carlo settings
☒	☐	For hierarchical and complex designs, identification of the appropriate level for tests and full reporting of outcomes
☐	☒	Estimates of effect sizes (e.g. Cohen's *d*, Pearson's *r*), indicating how they were calculated

Our web collection on statistics for biologists contains articles on many of the points above.

Software and code

Policy information about availability of computer code

Data collection	No software was used for data collection.
Data analysis	Pearson's correlation analyses were performed with OriginPro (version 2022, https://www.originlab.com/).<br><br>The taxonomic tree was obtained from NCBI (https://www.ncbi.nlm.nih.gov/Taxonomy/CommonTree/wwwcmt.cgi). Phylogenetic tree of the 350 species are generated by phyloT (https://phylot.biobyte.de/) based on NCBI taxonomy database. Phylogenetics trees are visualised and figures are generated by iTOL.<br><br>All other analyses were done as described with publicly available tools (hmmer, tmhmm , diamond, FastTree, FAMSA, gotree, R).<br>hmmer, version 3.1b2<br>tmhmm, version 2.0<br>diamond, version 0.9.26<br>FastTree, version 2.1.11 SSE3<br>FAMSA, version 2.1.11<br>R, version 3.4.4<br>gotree, v0.4.2<br><br>Scripts will be available on github.com/MWSchmid/Ngou-et-al.-2022

For manuscripts utilizing custom algorithms or software that are central to the research but not yet described in published literature, software must be made available to editors and reviewers. We strongly encourage code deposition in a community repository (e.g. GitHub). See the Nature Portfolio guidelines for submitting code & software for further information.

Data

Policy information about availability of data

All manuscripts must include a data availability statement. This statement should provide the following information, where applicable:

- Accession codes, unique identifiers, or web links for publicly available datasets
- A description of any restrictions on data availability
- For clinical datasets or third party data, please ensure that the statement adheres to our policy

All data generated or analysed during this study are included in the article or supplementary information files. Proteomes of 350 species used in this study are downloaded from either NCBI, Phyozome13, ensemblplants, JGI, Fernbase, Penium Genome Database or directly from the publications. A complete list of the proteomes and associated data used in this study are provided in Supplementary Table 1. Sequences of the identified receptors and phylogenetic analyses are available on Zenodo (https://doi.org/10.1101/2022.01.01.474684).

Field-specific reporting

Please select the one below that is the best fit for your research. If you are not sure, read the appropriate sections before making your selection.

- [x] Life sciences
- [ ] Behavioural & social sciences
- [ ] Ecological, evolutionary & environmental sciences

For a reference copy of the document with all sections, see nature.com/documents/nr-reporting-summary-flat.pdf

Life sciences study design

All studies must disclose on these points even when the disclosure is negative.

Sample size	The sample size was determined by the number of publicly available plant genome assemblies. 350 species with a representative genome assembly were included.
Data exclusions	No data were excluded from the analyses.
Replication	Does not apply as this study does not comprise a planned experiment with experimental groups. We selected all species for which a high-quality proteome was publicly available at the time of the study. Only bioinformatic approach was used in this study, thus we believe that replicability does not apply in this context.
Randomization	Does not apply as this study does not comprise a planned experiment with experimental groups. We selected all species for which a high-quality proteome was publicly available at the time of the study.
Blinding	We analyzed data from previously published studies and we did not include any blinding measures. We were not involved in the sample handling or sequencing of these genomes. Thus, blinding was not relevant to this study.

Reporting for specific materials, systems and methods

We require information from authors about some types of materials, experimental systems and methods used in many studies. Here, indicate whether each material, system or method listed is relevant to your study. If you are not sure if a list item applies to your research, read the appropriate section before selecting a response.

Materials & experimental systems

n/a	Involved in the study	
☒	☐	Antibodies
☒	☐	Eukaryotic cell lines
☒	☐	Palaeontology and archaeology
☒	☐	Animals and other organisms
☒	☐	Human research participants
☒	☐	Clinical data
☒	☐	Dual use research of concern

Methods

n/a	Involved in the study	
☒	☐	ChIP-seq
☒	☐	Flow cytometry
☒	☐	MRI-based neuroimaging
